# Supplementary material for: Recycled melanoma-secreted melanosomes regulate tumor-associated macrophage diversification
Source: EMBO J. 2024 May 8;43(17):3. doi: 10.1038/s44318-024-00103-7 (PMC11377571; doi:10.1038/s44318-024-00103-7)
Supplement: Supplementary file 1 — Appendix [file 44318_2024_103_MOESM1_ESM.pdf]

Recycled melanoma-secreted melanosomes regulate tumor-associated macrophage diversification

Appendix Materials

Content

Content	Page No.
Appendix Figure S1	2
Appendix Figure S2	3
Appendix Table S1	4
Appendix Figure S3	5
Appendix Figure S4	6
Appendix Figure S5	7

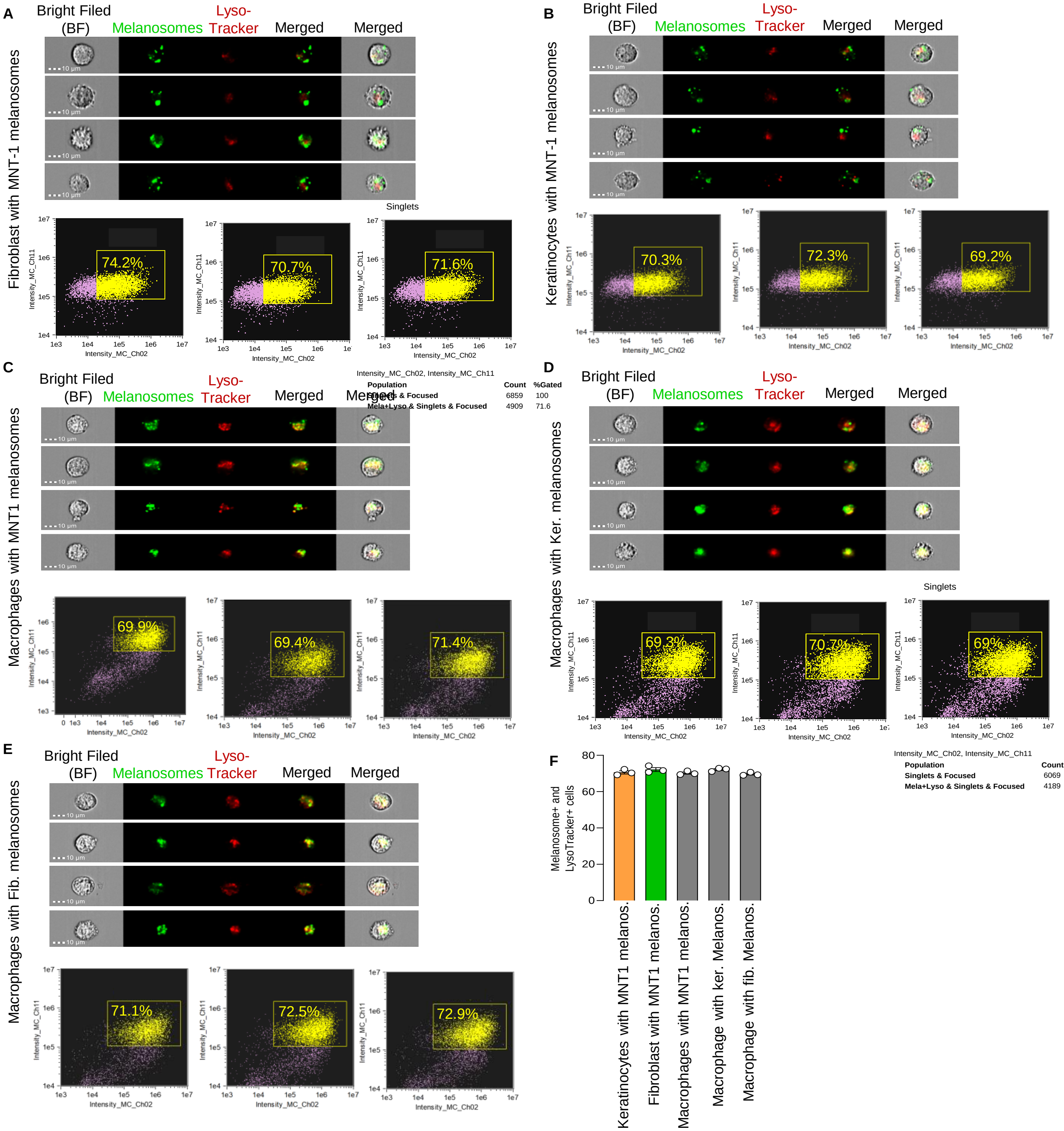

Appendix Figure S1, associated with Figure 2: Cell-to-cell transfer of melanoma cell-derived melanosomes occurs in the tumor microenvironment. (A-E) Representative ImageStream cytometry images of A) fibroblast cultured with Pkh67-labeled melanosomes from MNT1 melanoma cells, B) keratinocyte cultured with Pkh67-labeled melanosomes from MNT1 melanoma cells, C) macrophage cultured with Pkh67-labeled melanosomes from MNT1 melanoma cells, D) macrophage cultured with Pkh67-labeled keratinocyte-derived melanosomes, and E) macrophage cultured with Pkh67-labeled fibroblast-derived melanosomes. Cells were stained with the LysoTraker Deep red dye. Scale bar = 10 µm. **(F)** Percentage co-localization of the melanosomes (melanos.) with the LysoTracker within each cell type. Error bars represent ± S.E.M. n=3.

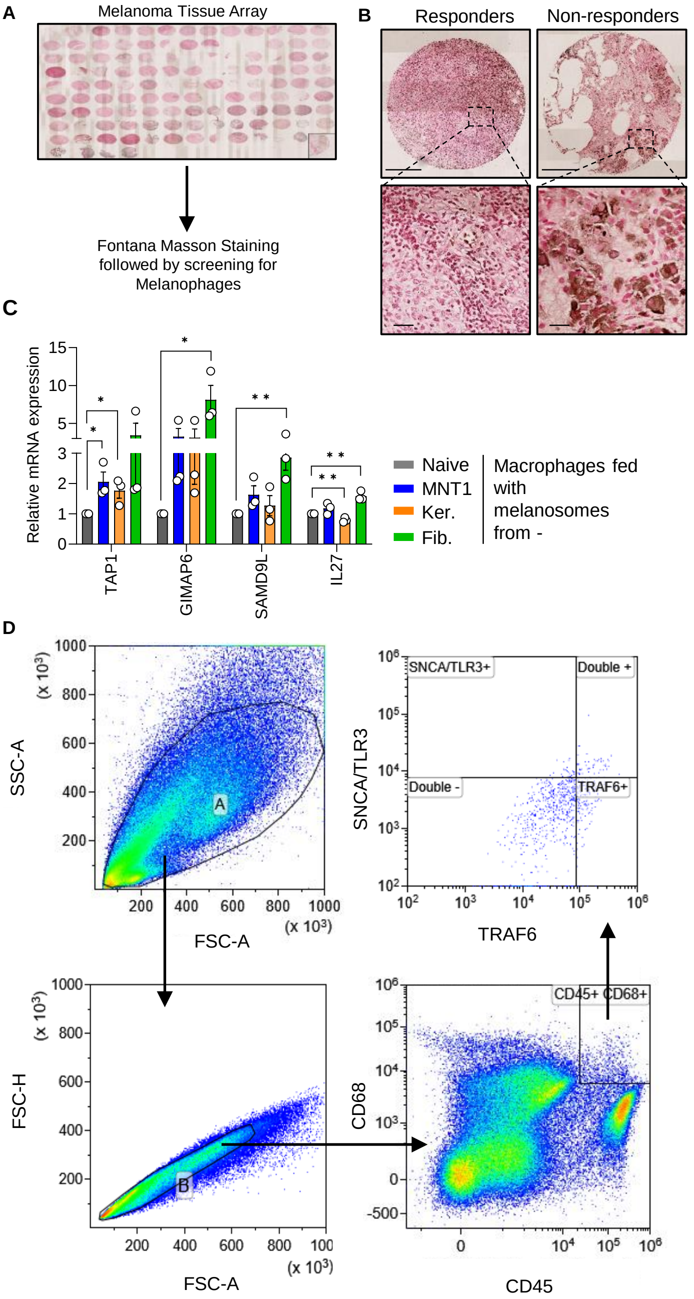

Appendix Figure S2, associated with Figure 6: The melanosome-induced macrophage polarization signature is detected in human melanoma specimens.

(A) Schematic workflow of screening the melanoma tissue array. **(B)** Images of Fontana Masson-stained representative patient specimens. Scale bars, 400 µm. Enlarged images show the area containing macrophages with pigment vesicles. Scale bars, 80 µm. **(C)** Relative mRNA expression of *TAP1, GIMAP6, SAMD9L, and IL27* in macrophages cultured with melanosomes from MNT1 melanoma cells, keratinocytes, and fibroblasts normalized to levels in naïve macrophages. n=3. **(D)** Workflow for the flow cytometry analysis shown in Figure 6F. Analysis was performed to determine the percentage of gated macrophages ($CD68^{+}$) of $CD45^{+}$ cells that express TLR3, TRAF6, and SNCA which are signature marker for fibroblast, keratinocyte, and melanoma-derived melanosomes cultured with macrophages within fresh melanoma harboring lymph node biopsies compared to tumor-free sentinel lymph node of the same patient; n=1 patient with 3 replicated.

Data information: In panel (C) error bars represent ± S.E.M. One-way ANOVA was performed for statistical analysis. **P* ≤ 0.05, ***P* ≤ 0.01 was considered significant.

Primer sequence

Mouse Primers		Nucleotide sequence
ANG1	Forward	AAACCACACGGCCACCAT
	Reverse	GTCAGCTTTCGGGTCTGCTC
ANG2	Forward	TCAAGGCCATCTGTGGAAAG
	Reverse	CTTCCTTTGTGTGTGCAAGTG
mTOR	Forward	GGAGCCTTGCTGATCCT
	Reverse	CTGCTGCTGGGTGATCT
Tie1	Forward	CAAGGTCACACACACGGT
	Reverse	TGTTCTTCCAGATCACGTCAG
CD31	Forward	CCAGTGCAGAGCGGATAAT
	Reverse	ACTCGACAGGATGGAAATCAC

Human Primers		Nucleotide sequence
IL27	Forward	GGACCAACATGGAGAGGATG
	Reverse	GGTTGAATCCTGCAGCCA
SAMD9L	Forward	ACAGCCATCGCTACATAGAAC
	Reverse	CGTGGCTGTTTCTGTGTTTG
TAP1	Forward	CAATATGAGCACCGCTACCT
	Reverse	GGTCAGGCCATAGGCAATA
GIMAP6	Forward	GGAGCTGTCAGGAGGTCTAA
	Reverse	CTGTTGCACTCTTCCCACT
IL1B	Forward	ATGCACCTGTACGATCACTG
	Reverse	ACAAAGGACATGGAGAACACC
TNF	Forward	CAAGCCTGTAGCCCATGTT
	Reverse	TCAGCTCCACGCCATTG
IL6	Forward	GTAGTGAGGAACAAGCCAGAG
	Reverse	GCATTTGTGGTTGGGTCAG

Appendix Table S1: Primer sequence used for qRT-PCR for gene expression analysis.

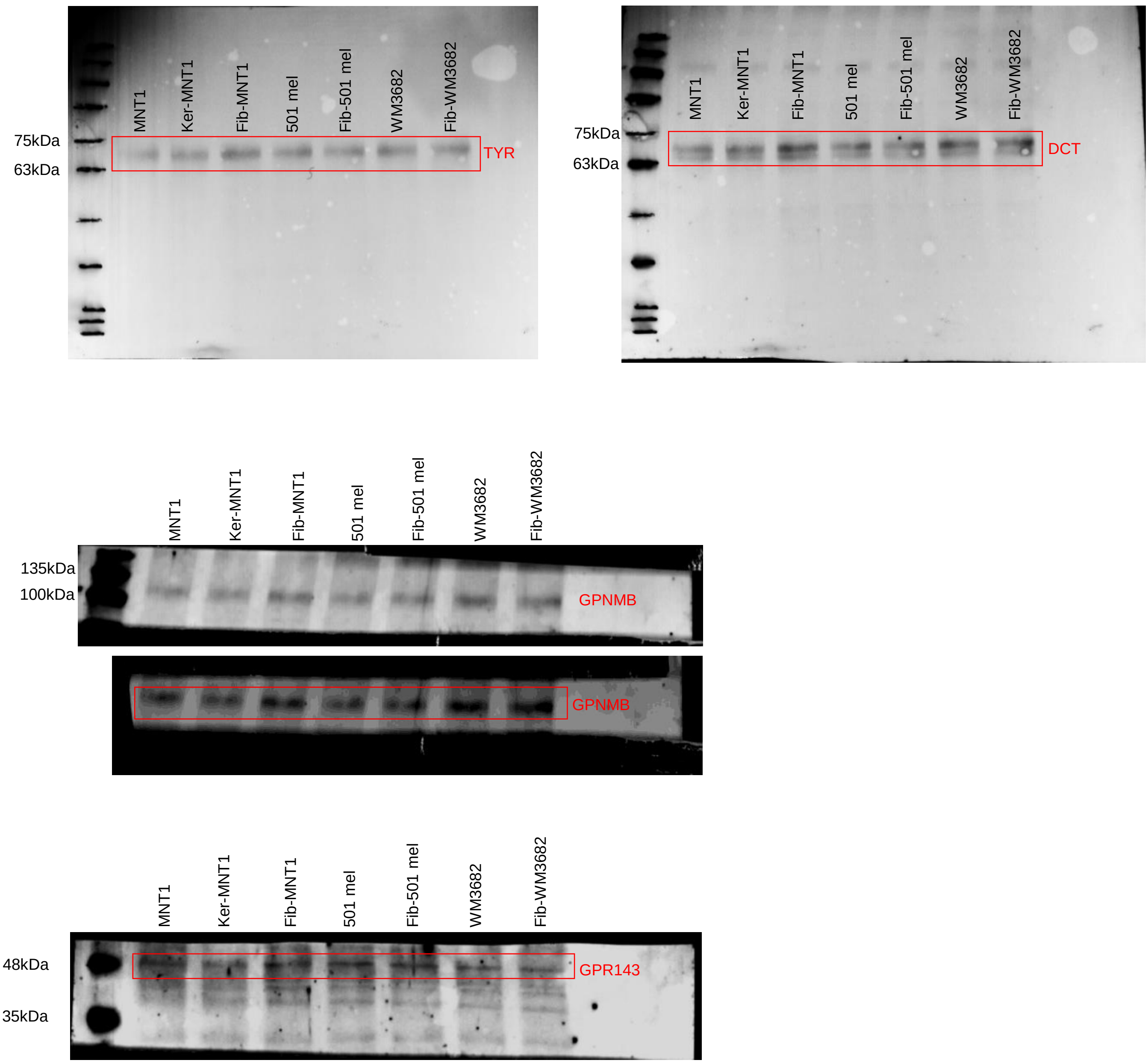

Appendix Figure S3: Unprocessed scans from the western blot experiments from Figure EV2I for the proteins TYR, DCT, GPNMB, and GPR143 as seen in the respective melanosomes fraction.

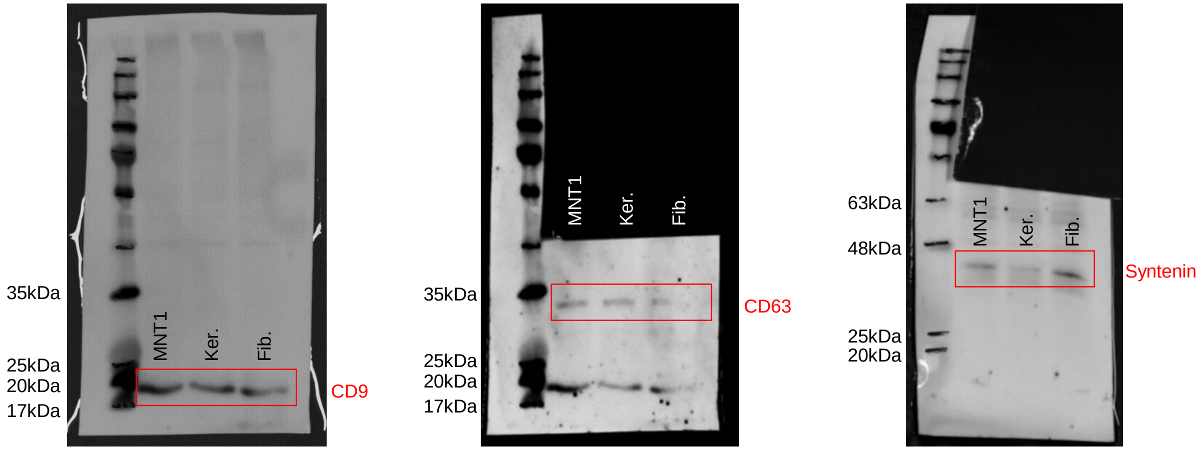

Appendix Figure S4: Unprocessed scans from the western blot experiments from Figure EV2J for the proteins CD9, CD63, and Syntenin as seen in the respective exosome fraction.

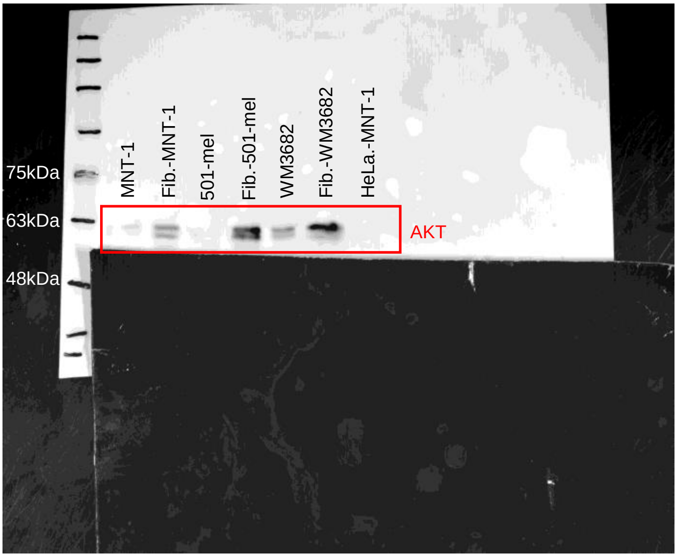

Appendix Figure S5: Unprocessed scans from the western blot experiment from Figure EV5E for the protein AKT as seen in the respective melanosomes fraction.
