# Supplementary material for: Recycled melanoma-secreted melanosomes regulate tumor-associated macrophage diversification
Source: EMBO J. 2024 May 8;43(17):3. doi: 10.1038/s44318-024-00103-7 (PMC11377571; doi:10.1038/s44318-024-00103-7)
Supplement: Supplementary file 2 — Movie EV1 [file 44318_2024_103_MOESM2_ESM.zip › Movie EV1.docx]

Movie EV1, related to the Main Figure 3F: Ultrasound imaging of the mouse with B16-F10 melanoma (control).
