# Supplementary material for: Recycled melanoma-secreted melanosomes regulate tumor-associated macrophage diversification
Source: EMBO J. 2024 May 8;43(17):3. doi: 10.1038/s44318-024-00103-7 (PMC11377571; doi:10.1038/s44318-024-00103-7)
Supplement: Supplementary file 3 — Movie EV2 [file 44318_2024_103_MOESM3_ESM.zip › Movie EV2.docx]

Movie EV2, related to the Main Figure 3F: Ultrasound imaging of the mouse co-grafted with B16-F10 melanoma and naïve BMDMs (control).
