# Supplementary figures and images for: Recycled melanoma-secreted melanosomes regulate tumor-associated macrophage diversification (part 1 of 2)
Source: EMBO J. 2024 May 8;43(17):3. doi: 10.1038/s44318-024-00103-7 (PMC11377571; doi:10.1038/s44318-024-00103-7)

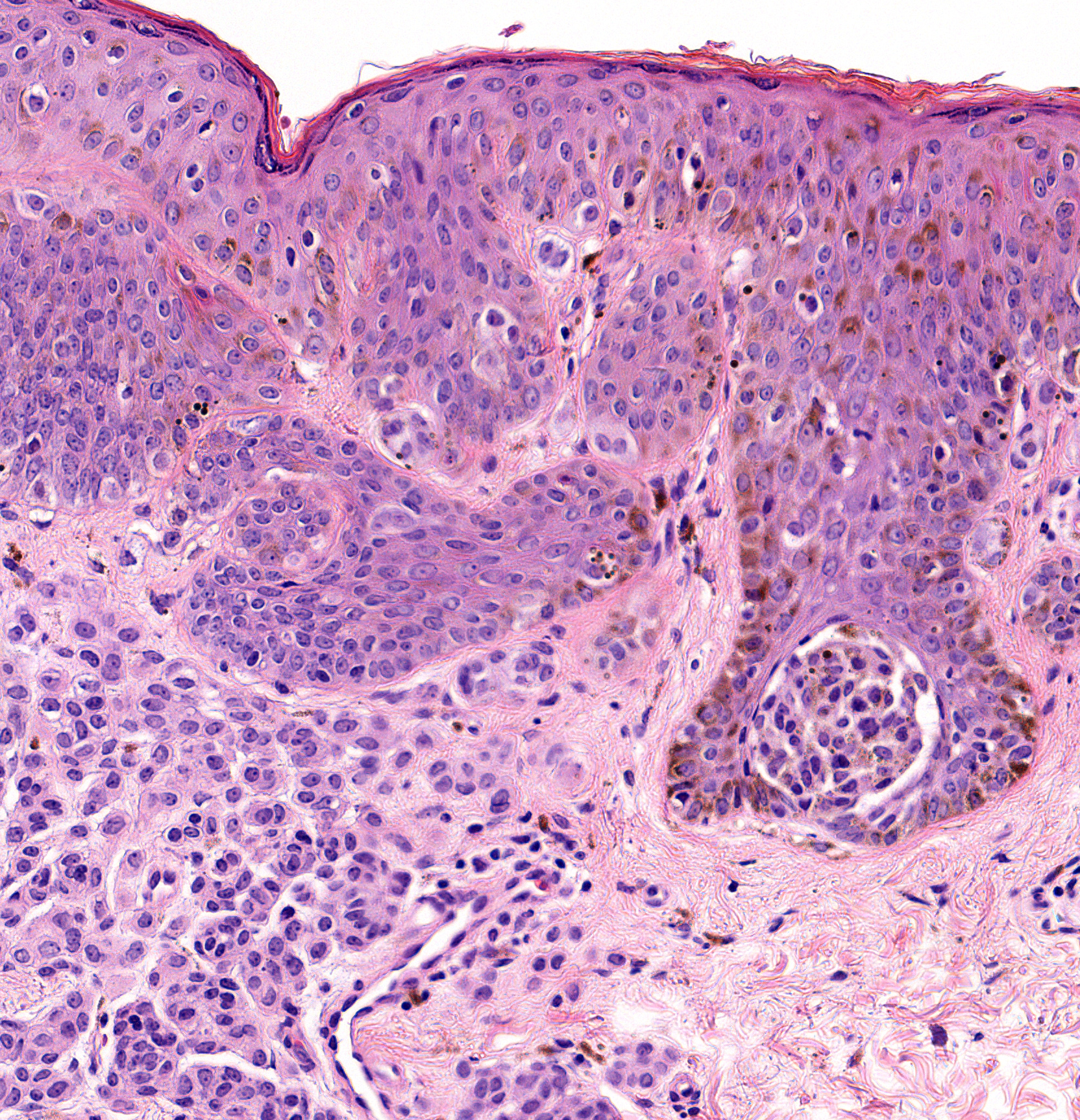

Supplement: Supplementary file 7 — Source data Fig. 1 [file 44318_2024_103_MOESM7_ESM.zip › Figure 1/1A/Compound Nevi/Compound nevi.tif]

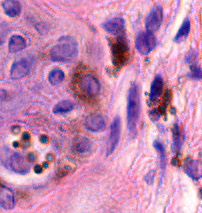

Supplement: Supplementary file 7 — Source data Fig. 1 [file 44318_2024_103_MOESM7_ESM.zip › Figure 1/1A/Compound Nevi/Inset image of Compound nevi.tif]

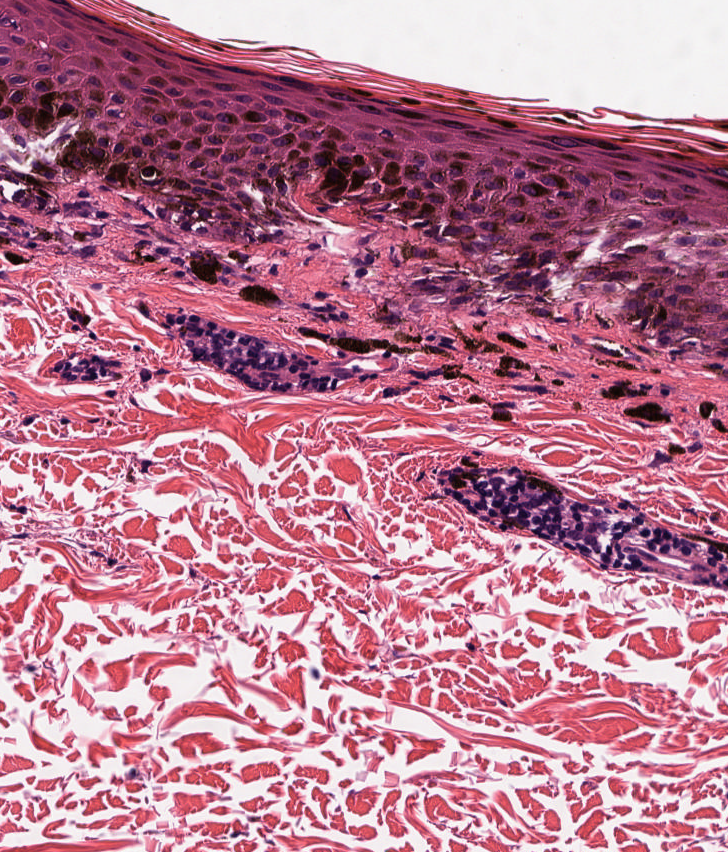

Supplement: Supplementary file 7 — Source data Fig. 1 [file 44318_2024_103_MOESM7_ESM.zip › Figure 1/1A/In Situ melanoma/In situ melanoma.tif]

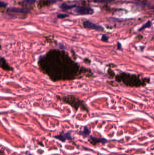

Supplement: Supplementary file 7 — Source data Fig. 1 [file 44318_2024_103_MOESM7_ESM.zip › Figure 1/1A/In Situ melanoma/Inset image of In Situ melanoma.tif]

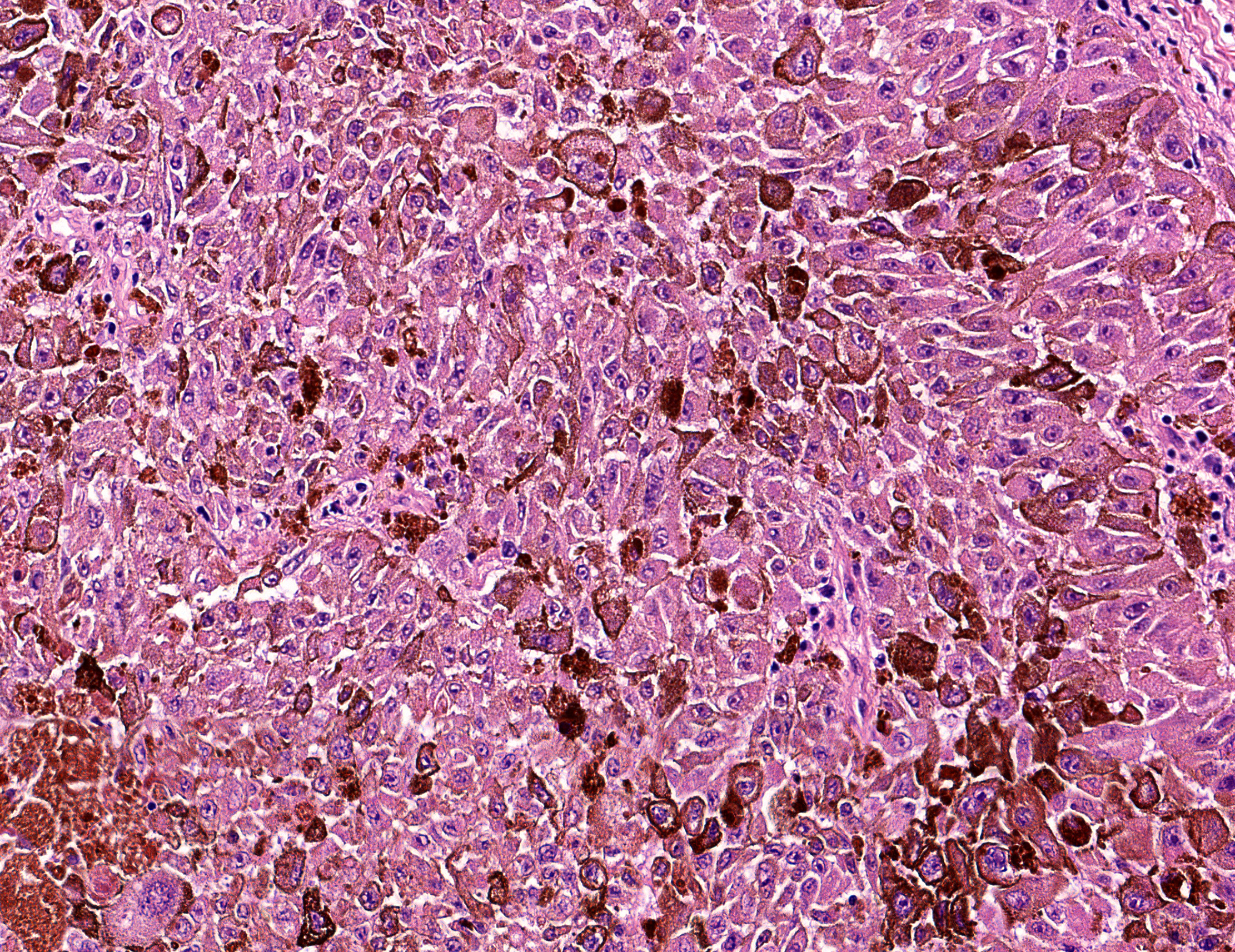

Supplement: Supplementary file 7 — Source data Fig. 1 [file 44318_2024_103_MOESM7_ESM.zip › Figure 1/1A/Lymph metastasis/H and E of Lymph metastasis.tif]

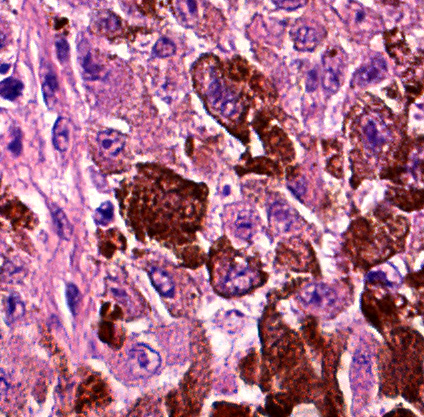

Supplement: Supplementary file 7 — Source data Fig. 1 [file 44318_2024_103_MOESM7_ESM.zip › Figure 1/1A/Lymph metastasis/Inset image of Lymph metastasis.tif]

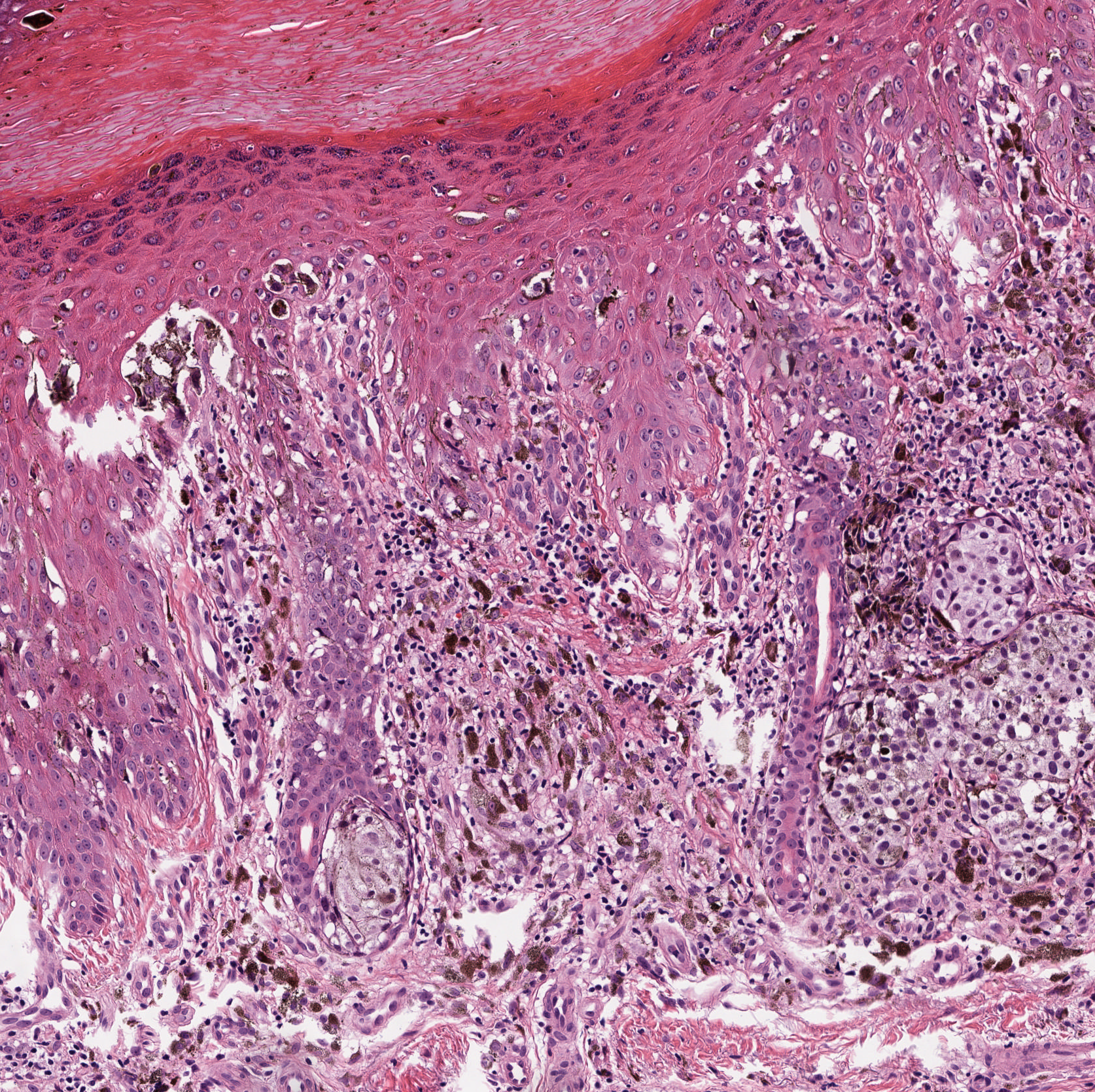

Supplement: Supplementary file 7 — Source data Fig. 1 [file 44318_2024_103_MOESM7_ESM.zip › Figure 1/1A/Vertical melanoma/H and E of Vertical melanoma.tif]

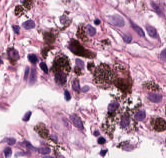

Supplement: Supplementary file 7 — Source data Fig. 1 [file 44318_2024_103_MOESM7_ESM.zip › Figure 1/1A/Vertical melanoma/Inset image of Vertical melanoma.tif]

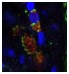

Supplement: Supplementary file 7 — Source data Fig. 1 [file 44318_2024_103_MOESM7_ESM.zip › Figure 1/1B/Compound Nevi/Crop Inset image.jpg]

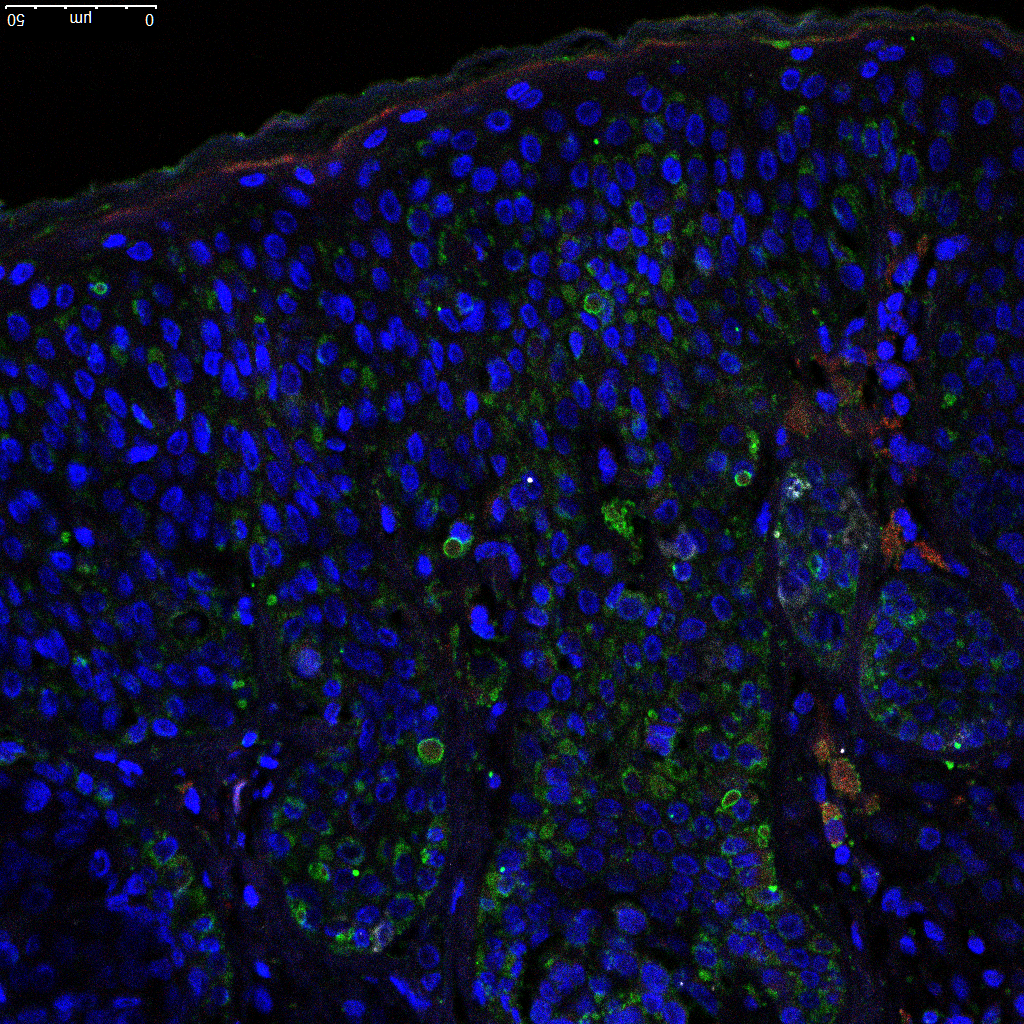

Supplement: Supplementary file 7 — Source data Fig. 1 [file 44318_2024_103_MOESM7_ESM.zip › Figure 1/1B/Compound Nevi/Image.tif]

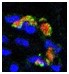

Supplement: Supplementary file 7 — Source data Fig. 1 [file 44318_2024_103_MOESM7_ESM.zip › Figure 1/1B/In Situ melanoma/Crop Inset image.jpg]

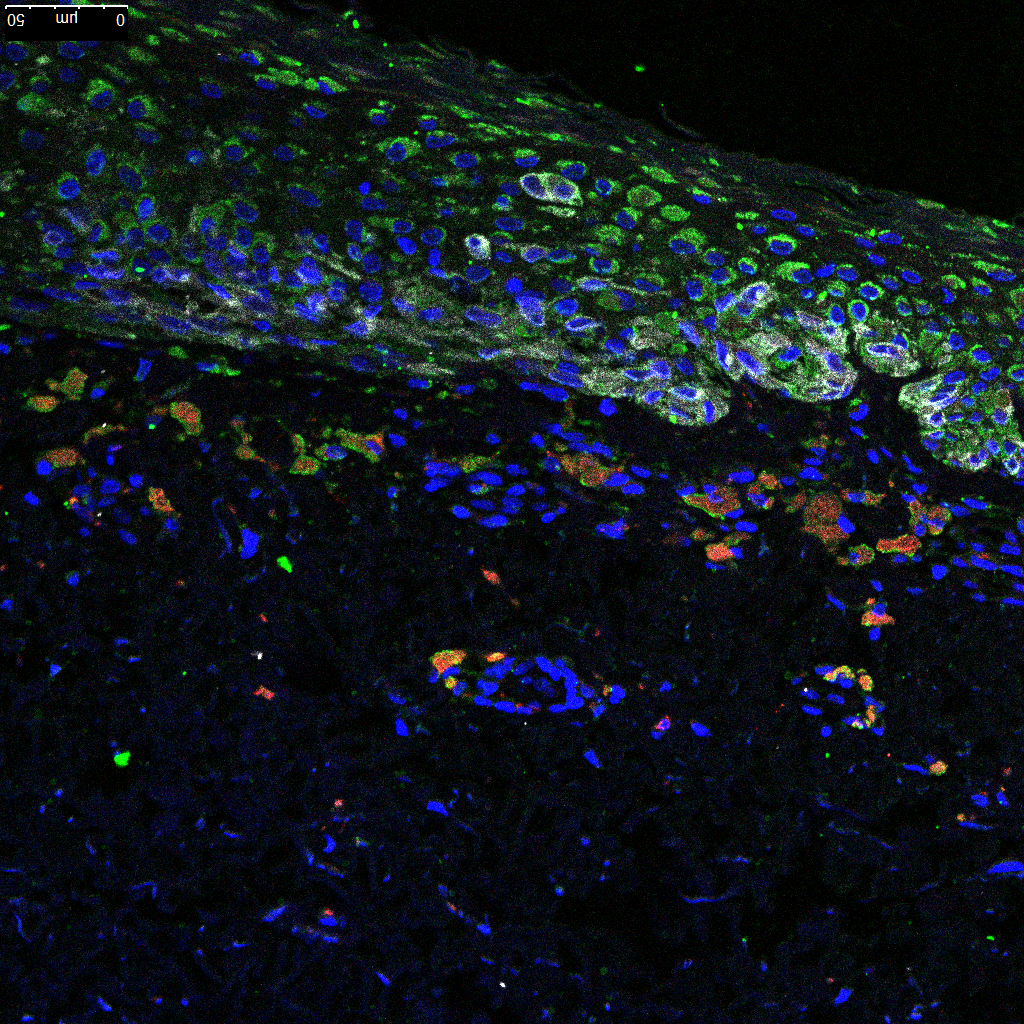

Supplement: Supplementary file 7 — Source data Fig. 1 [file 44318_2024_103_MOESM7_ESM.zip › Figure 1/1B/In Situ melanoma/Image.tif]

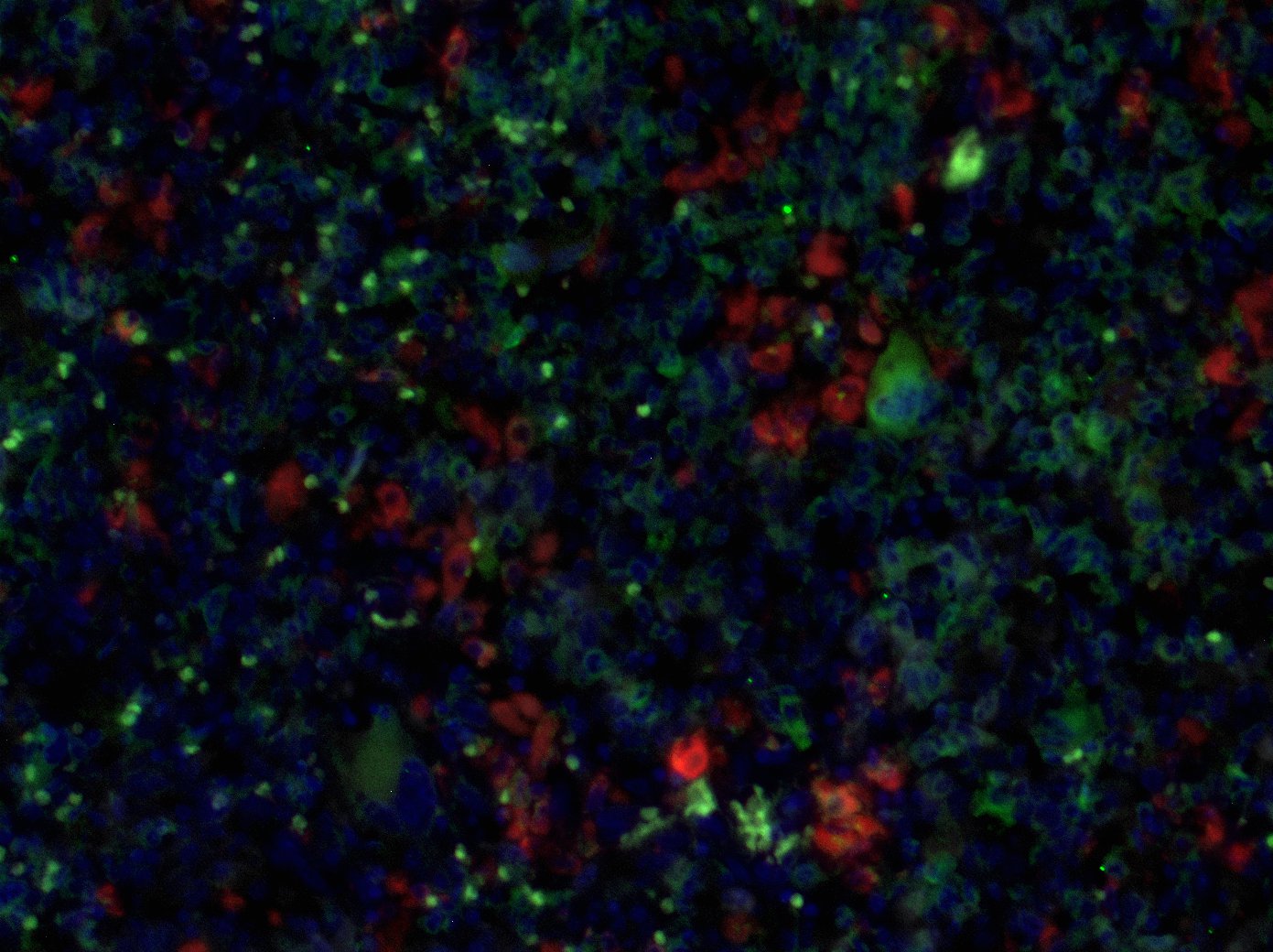

Supplement: Supplementary file 7 — Source data Fig. 1 [file 44318_2024_103_MOESM7_ESM.zip › Figure 1/1B/Lymph metastasis/Image.jpg]

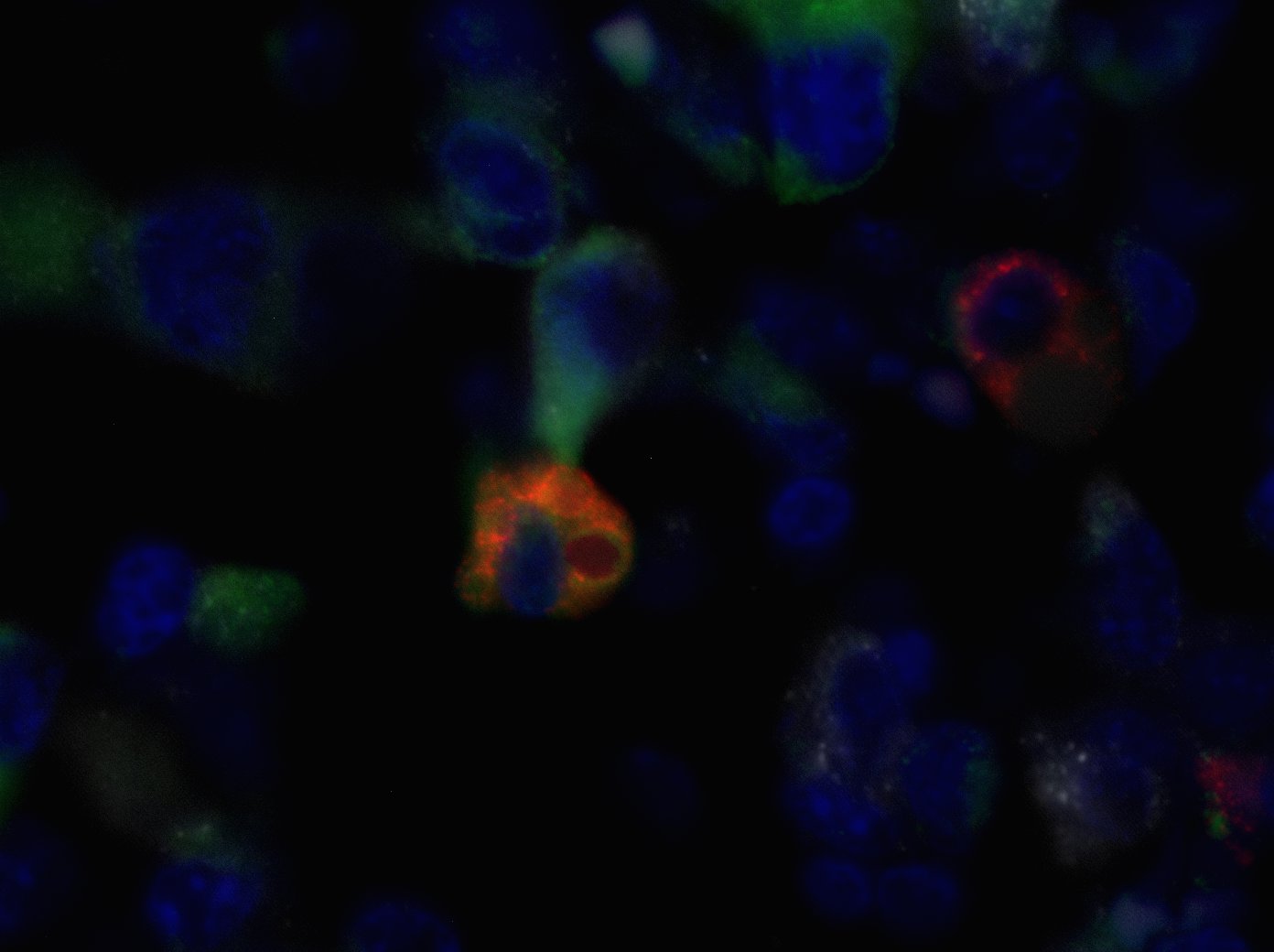

Supplement: Supplementary file 7 — Source data Fig. 1 [file 44318_2024_103_MOESM7_ESM.zip › Figure 1/1B/Lymph metastasis/Inset Image.jpg]

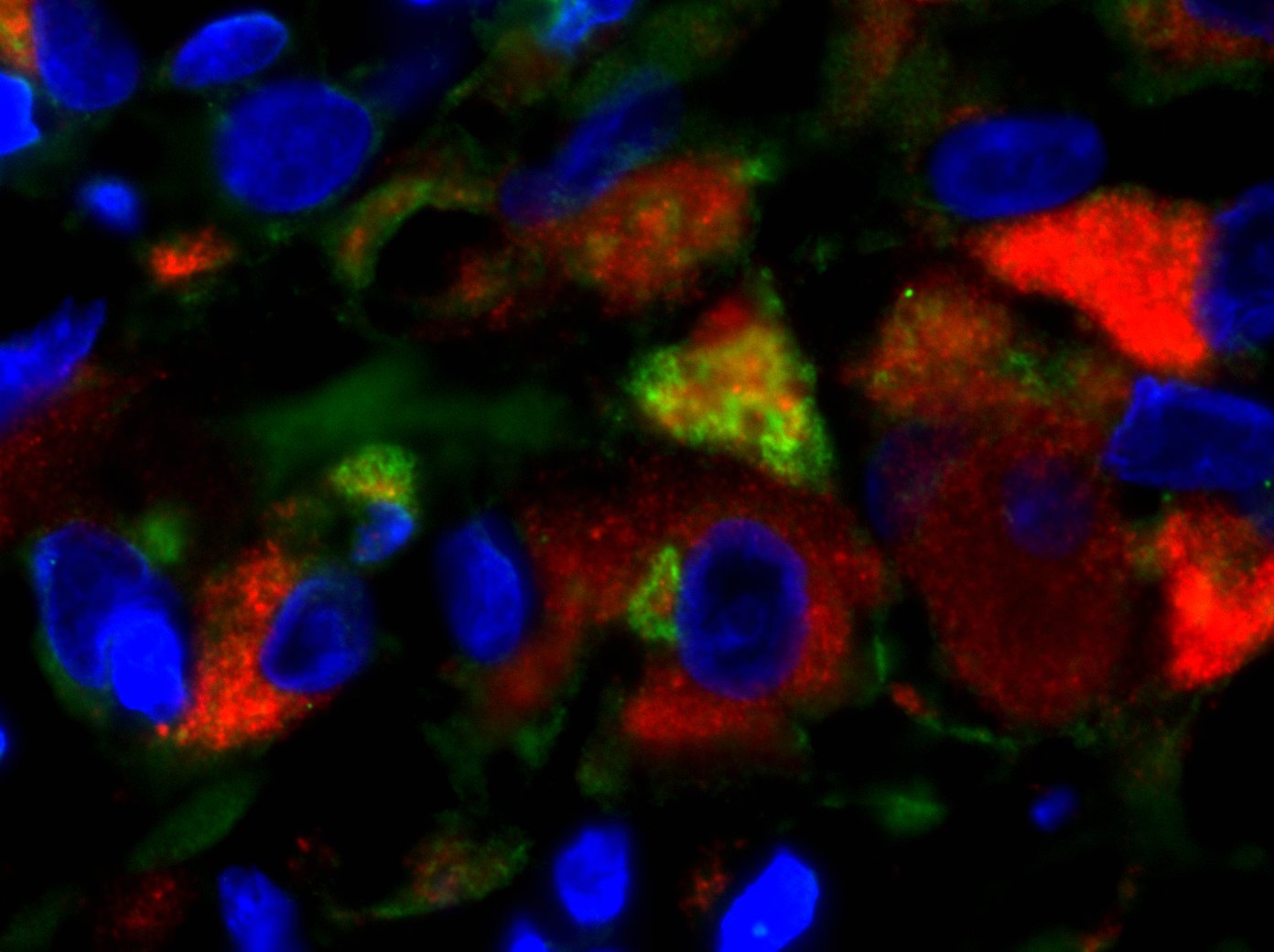

Supplement: Supplementary file 7 — Source data Fig. 1 [file 44318_2024_103_MOESM7_ESM.zip › Figure 1/1B/Vertical Melanoma/Image.jpg]

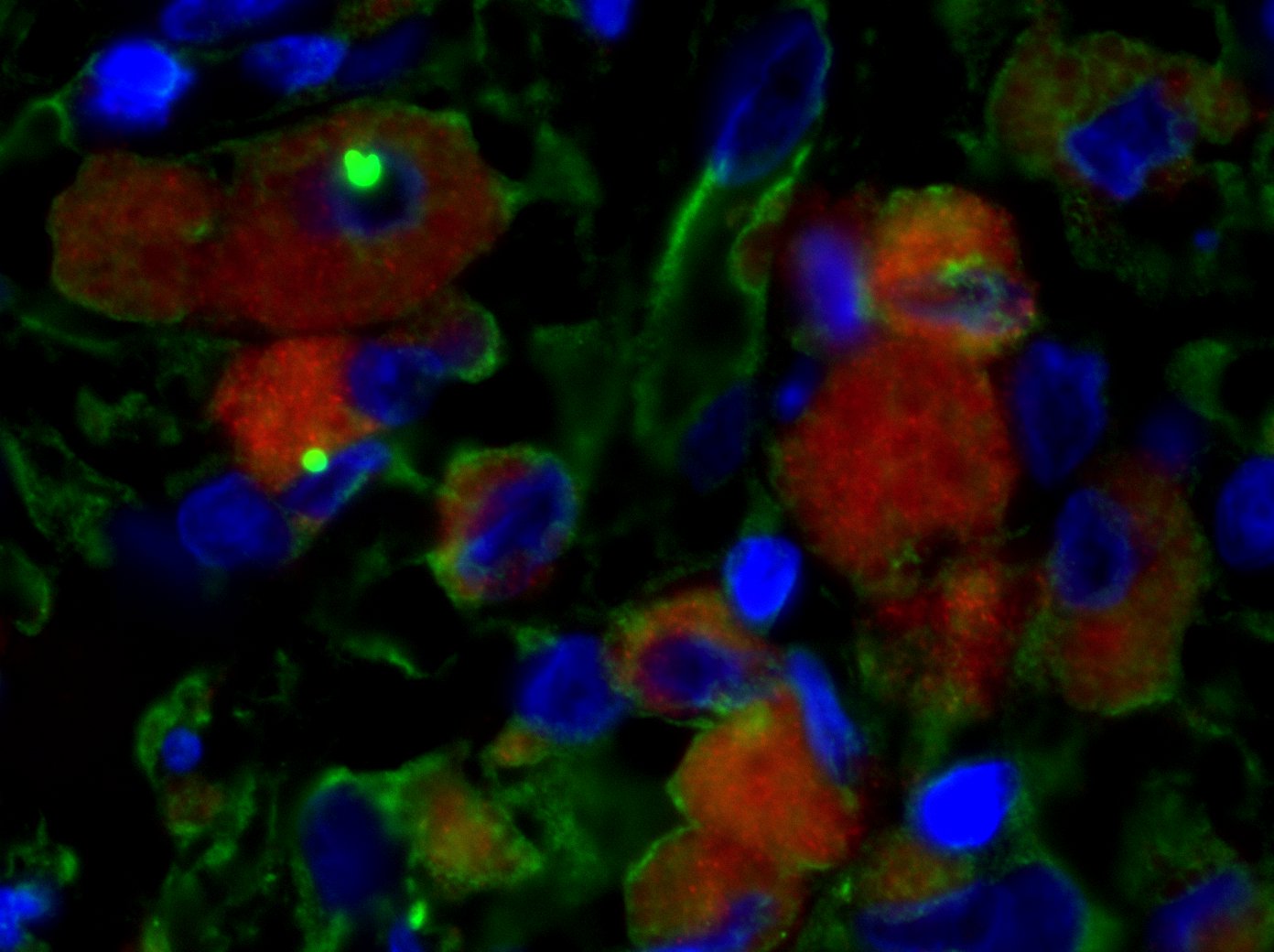

Supplement: Supplementary file 7 — Source data Fig. 1 [file 44318_2024_103_MOESM7_ESM.zip › Figure 1/1B/Vertical Melanoma/Inset image.jpg]

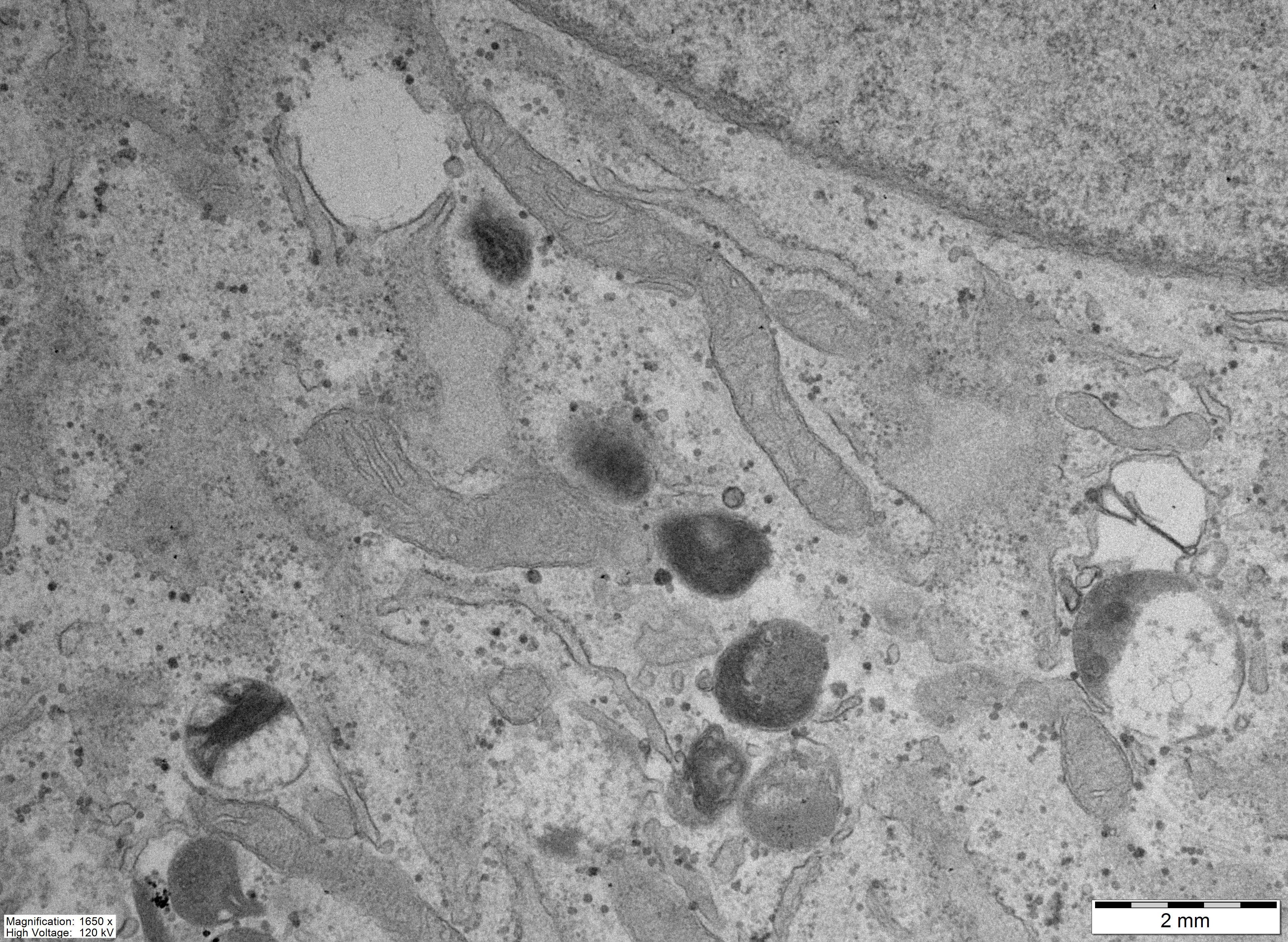

Supplement: Supplementary file 8 — Source data Fig. 2 [file 44318_2024_103_MOESM8_ESM.zip › Figure 2/2B/Fibroblast/Fibroblasts+melanosomes-88194-F2_005-18500.tif]

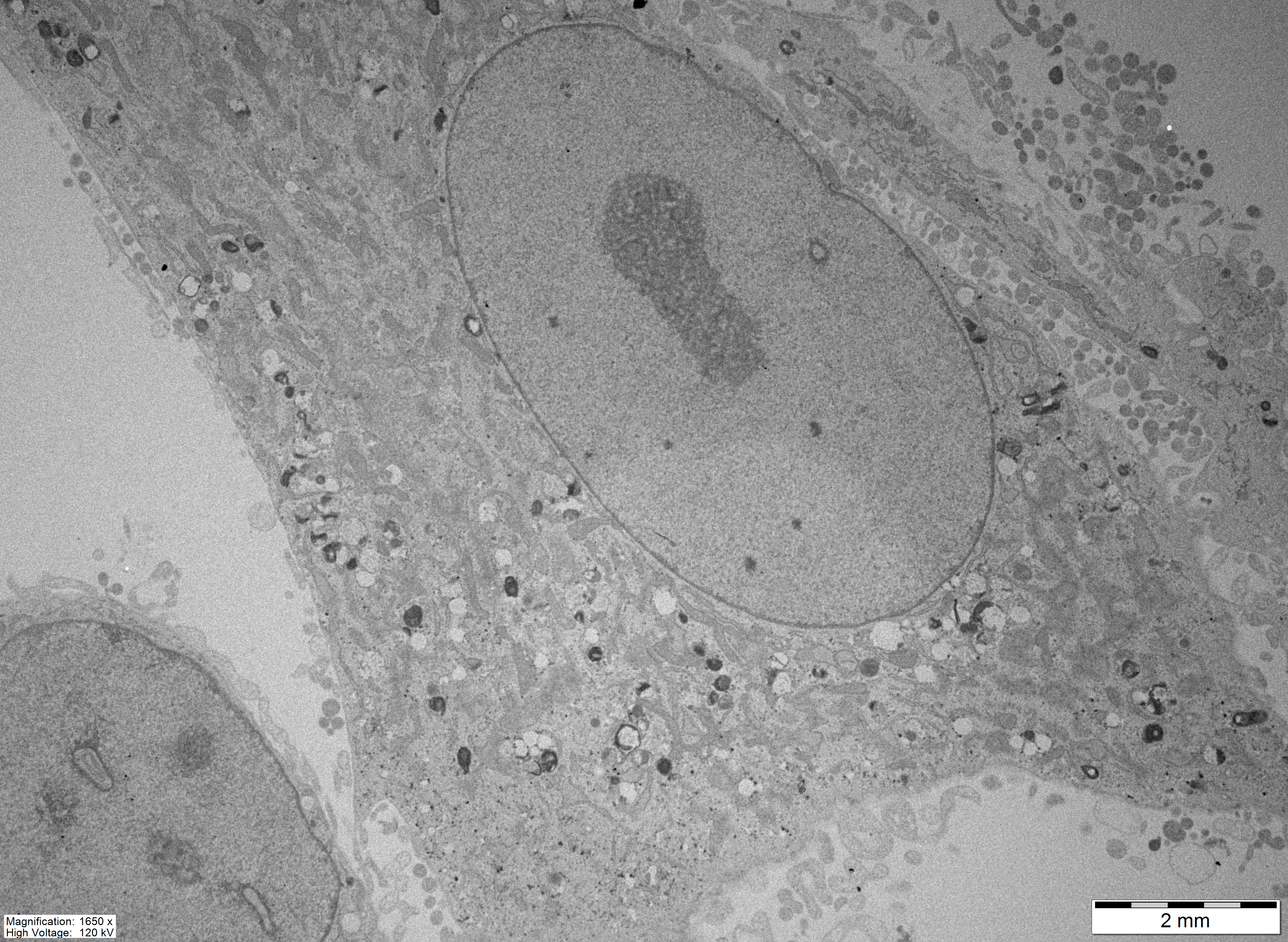

Supplement: Supplementary file 8 — Source data Fig. 2 [file 44318_2024_103_MOESM8_ESM.zip › Figure 2/2B/Fibroblast/Fibroblasts+melanosomes-88194-F2_006-2850.tif]

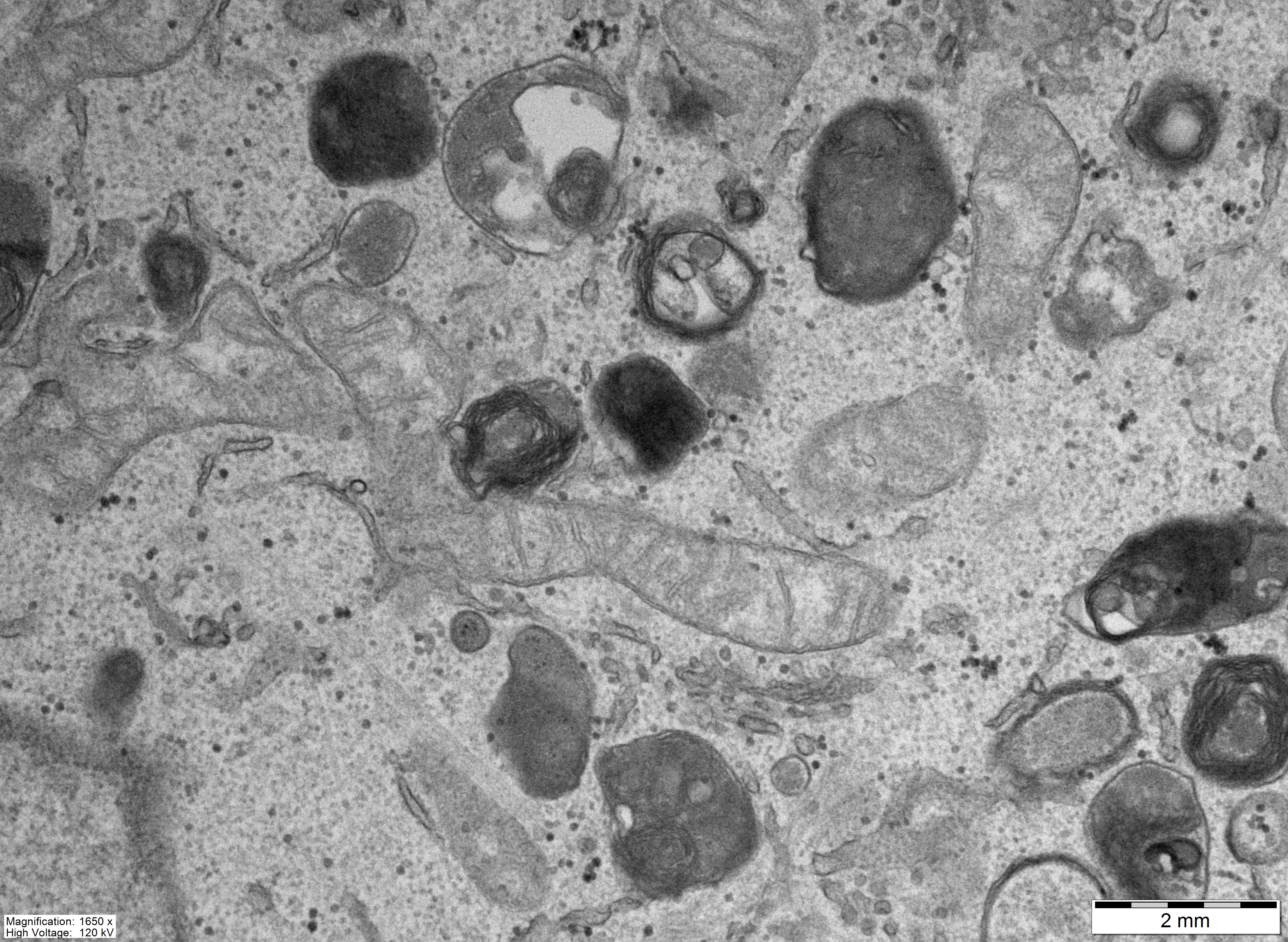

Supplement: Supplementary file 8 — Source data Fig. 2 [file 44318_2024_103_MOESM8_ESM.zip › Figure 2/2B/Keratinocytes/5-Keratinocytes+Melanosomes-84132-P1_005-18500.tif]

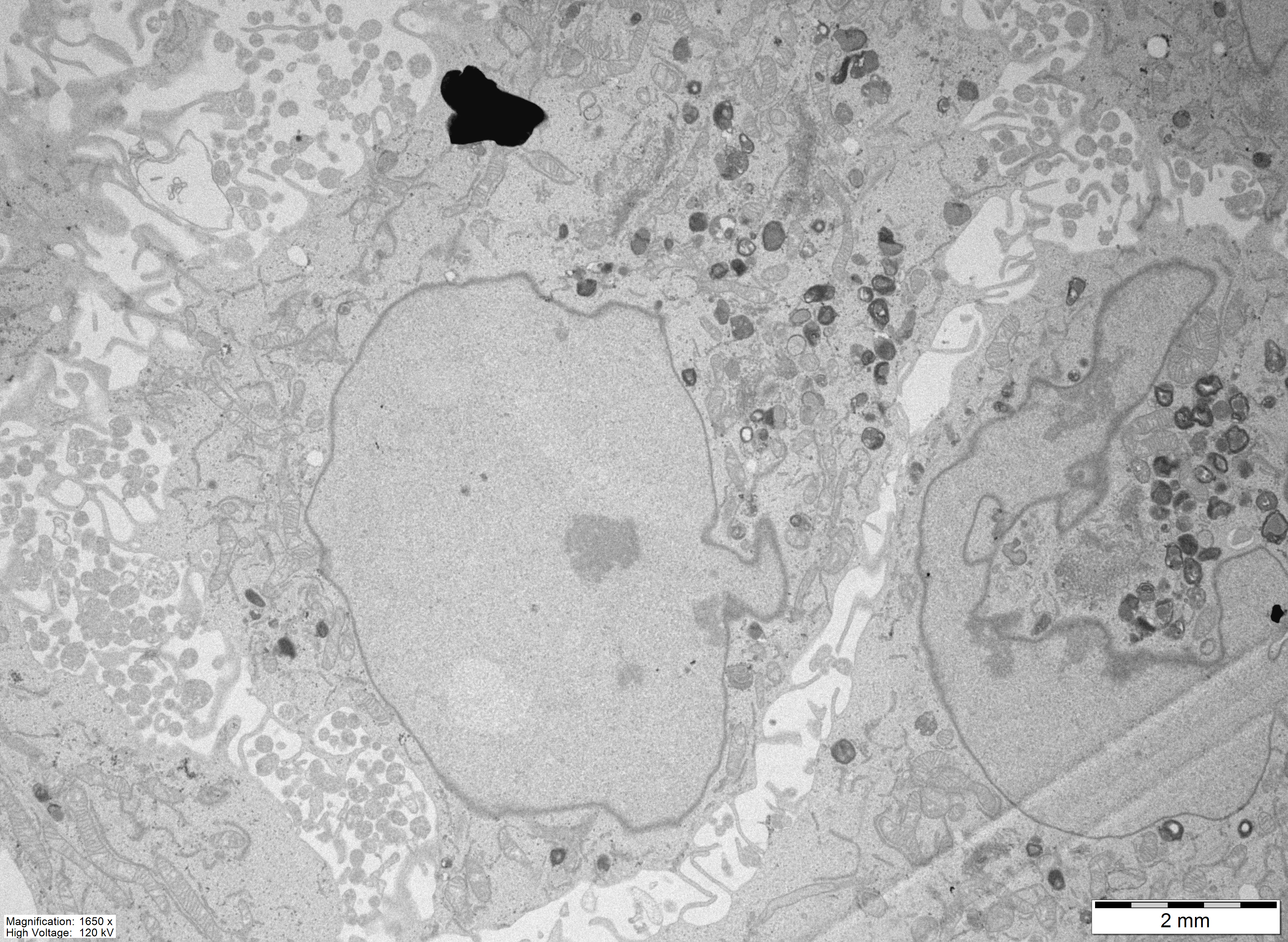

Supplement: Supplementary file 8 — Source data Fig. 2 [file 44318_2024_103_MOESM8_ESM.zip › Figure 2/2B/Keratinocytes/5-Keratinocytes+Melanosomes-84132-P1_006-2850.tif]

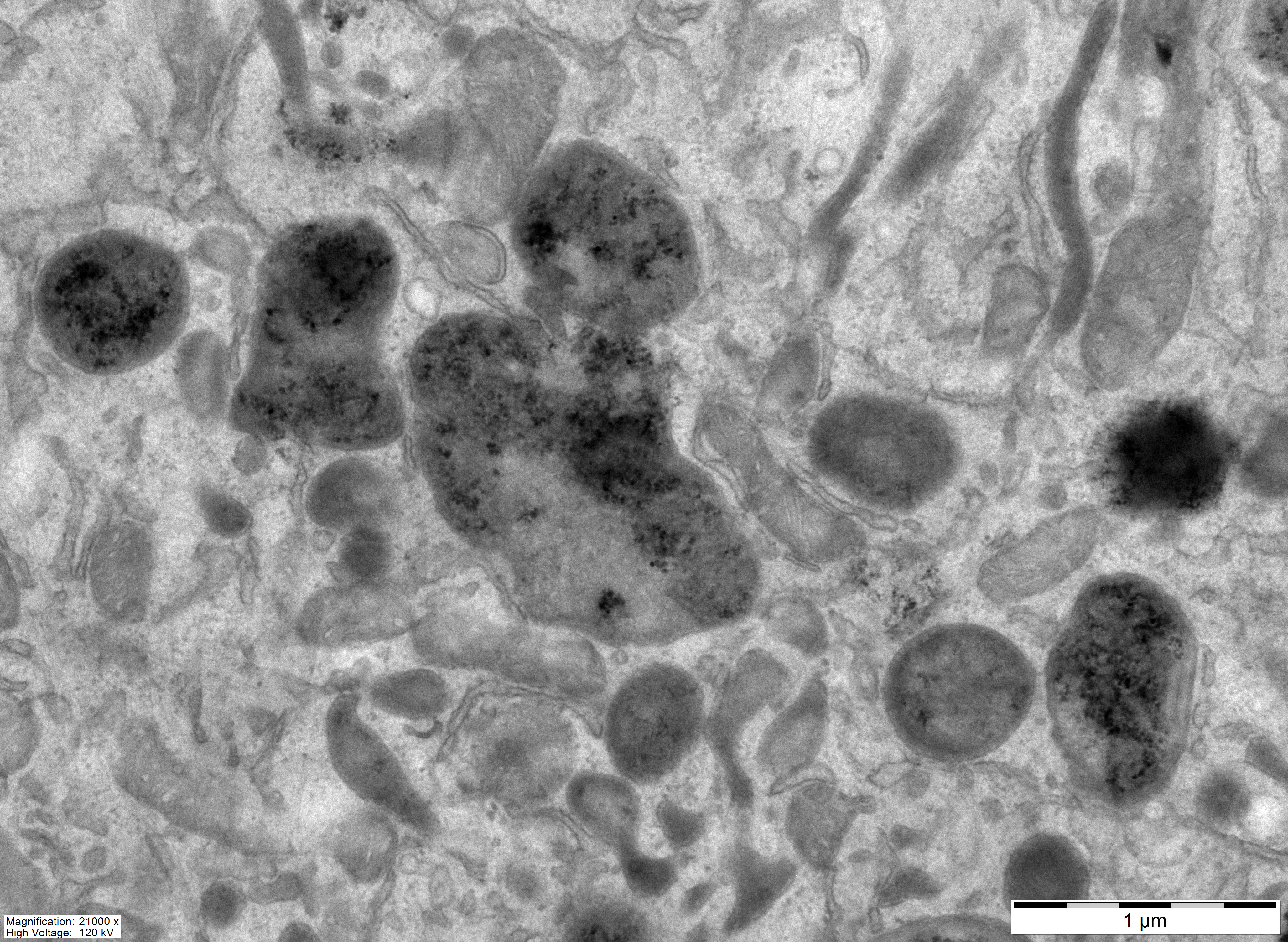

Supplement: Supplementary file 8 — Source data Fig. 2 [file 44318_2024_103_MOESM8_ESM.zip › Figure 2/2B/Macrophages/MDM+MNT-90004-C2_016-18500.tif]

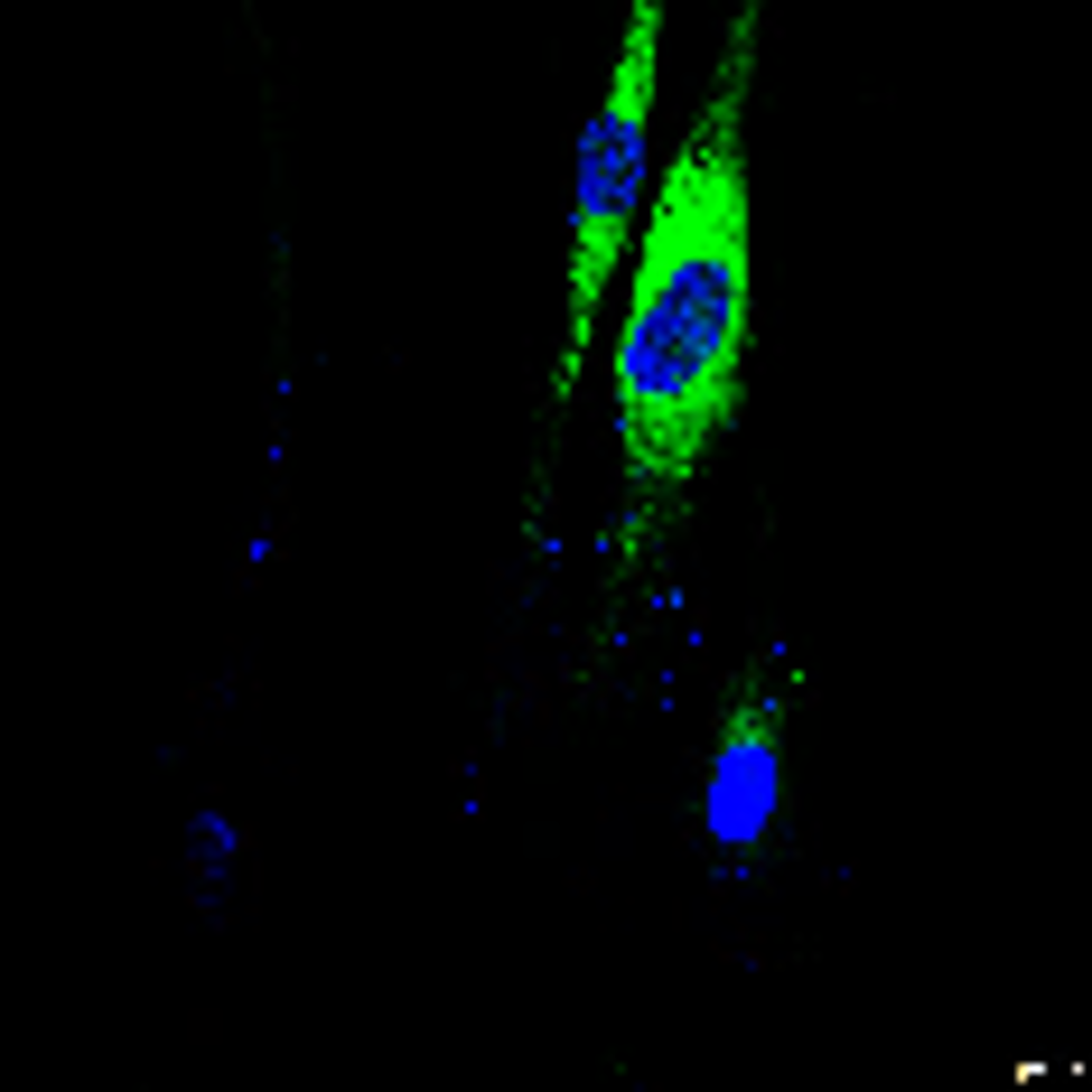

Supplement: Supplementary file 8 — Source data Fig. 2 [file 44318_2024_103_MOESM8_ESM.zip › Figure 2/2D/Fibroblasts/Melanosome cultured/Day 0.tif]

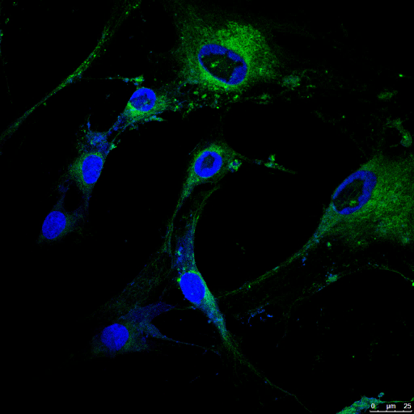

Supplement: Supplementary file 8 — Source data Fig. 2 [file 44318_2024_103_MOESM8_ESM.zip › Figure 2/2D/Fibroblasts/Melanosome cultured/Day 1.tif]

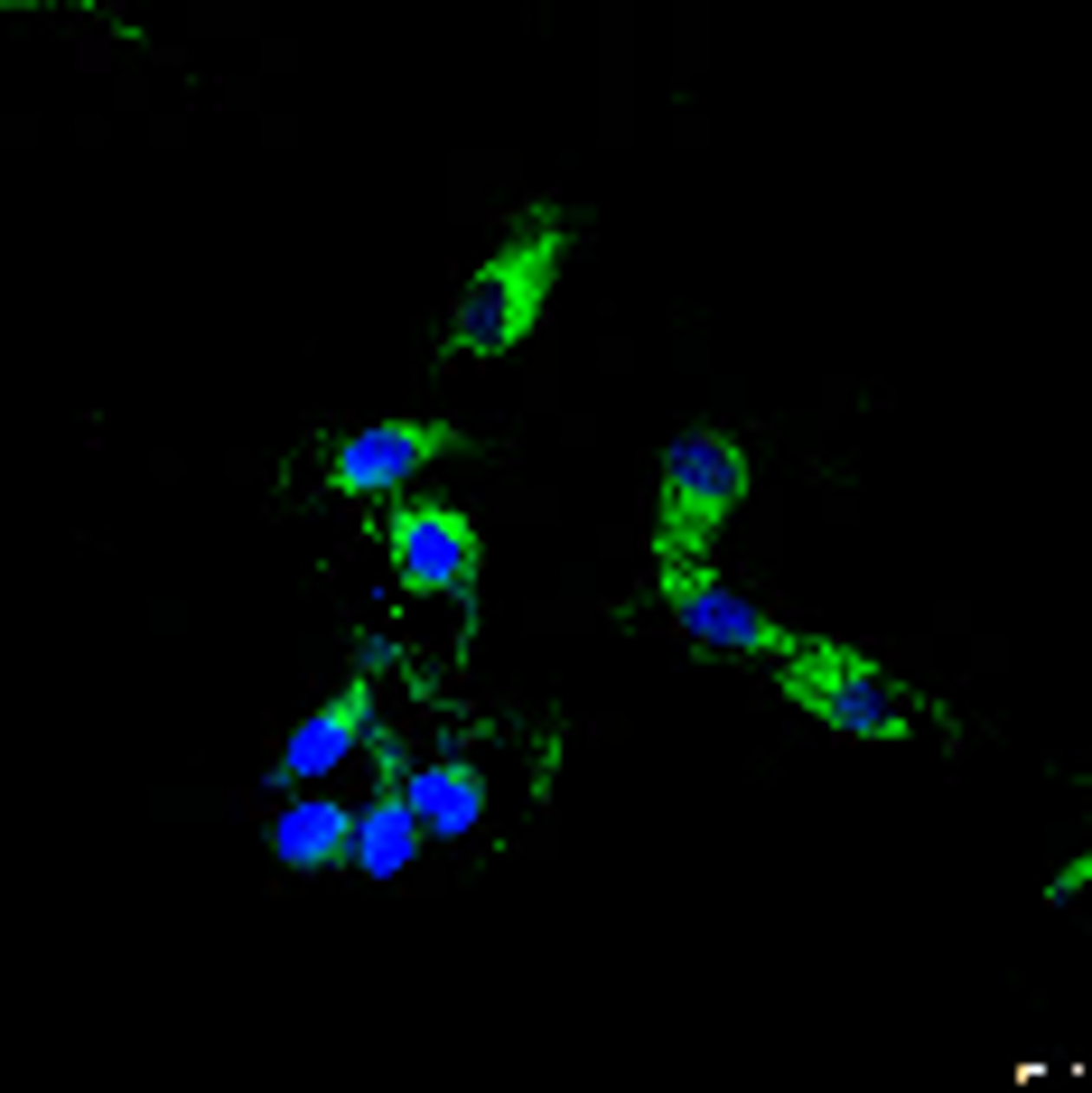

Supplement: Supplementary file 8 — Source data Fig. 2 [file 44318_2024_103_MOESM8_ESM.zip › Figure 2/2D/Fibroblasts/Melanosome cultured/Day 2.tif]

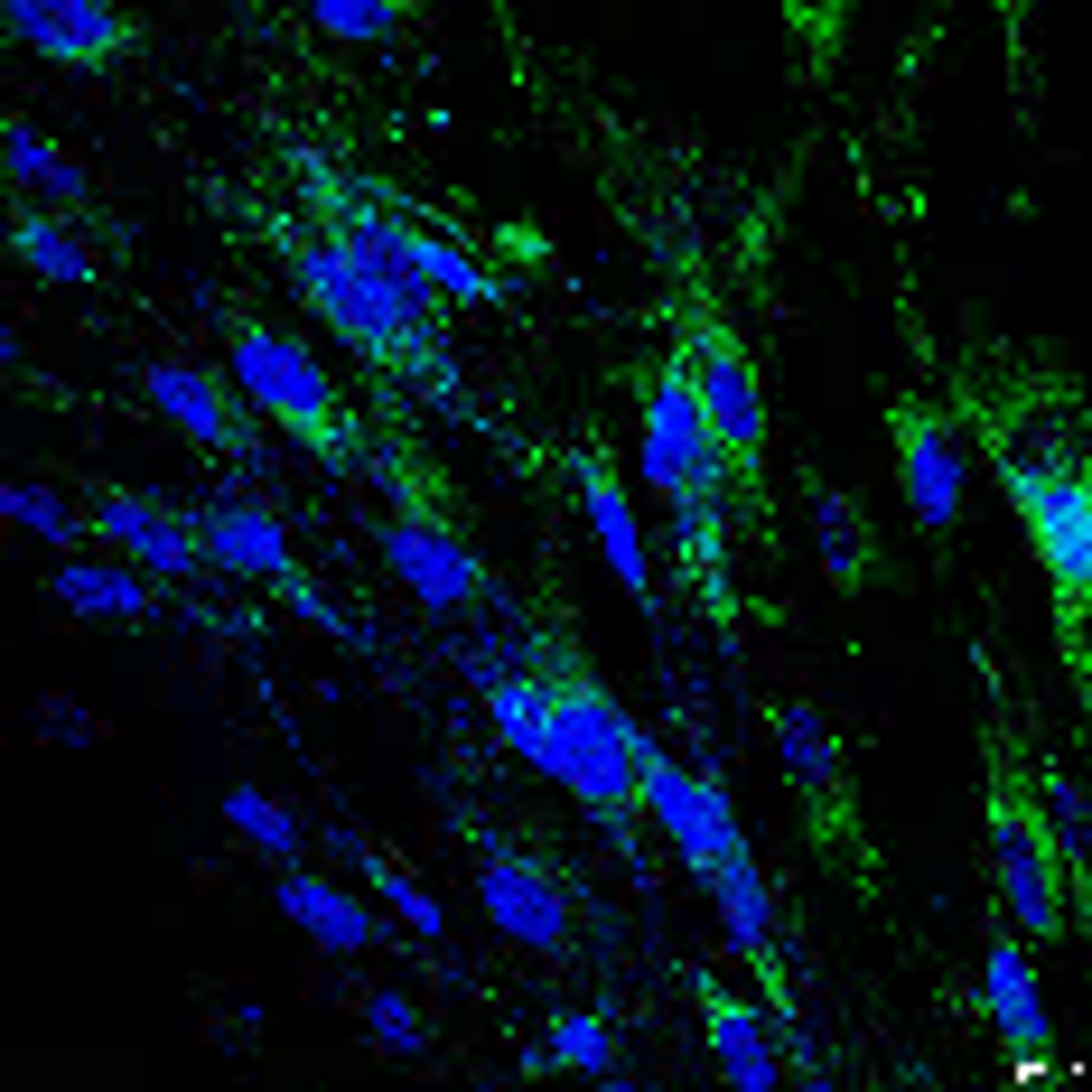

Supplement: Supplementary file 8 — Source data Fig. 2 [file 44318_2024_103_MOESM8_ESM.zip › Figure 2/2D/Fibroblasts/Melanosome cultured/Day 3.tif]

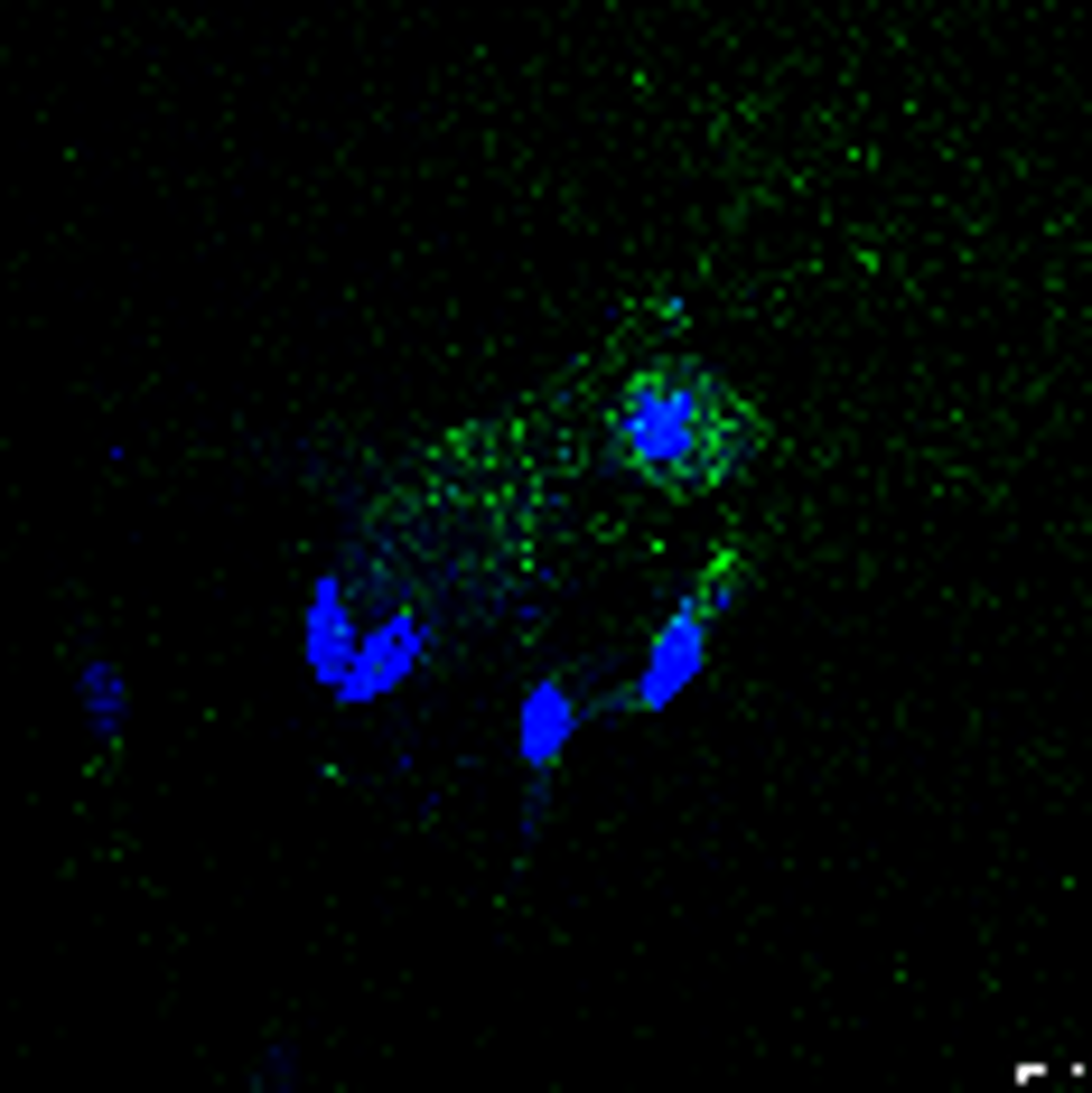

Supplement: Supplementary file 8 — Source data Fig. 2 [file 44318_2024_103_MOESM8_ESM.zip › Figure 2/2D/Fibroblasts/Small EV cultured/Day 0.tif]

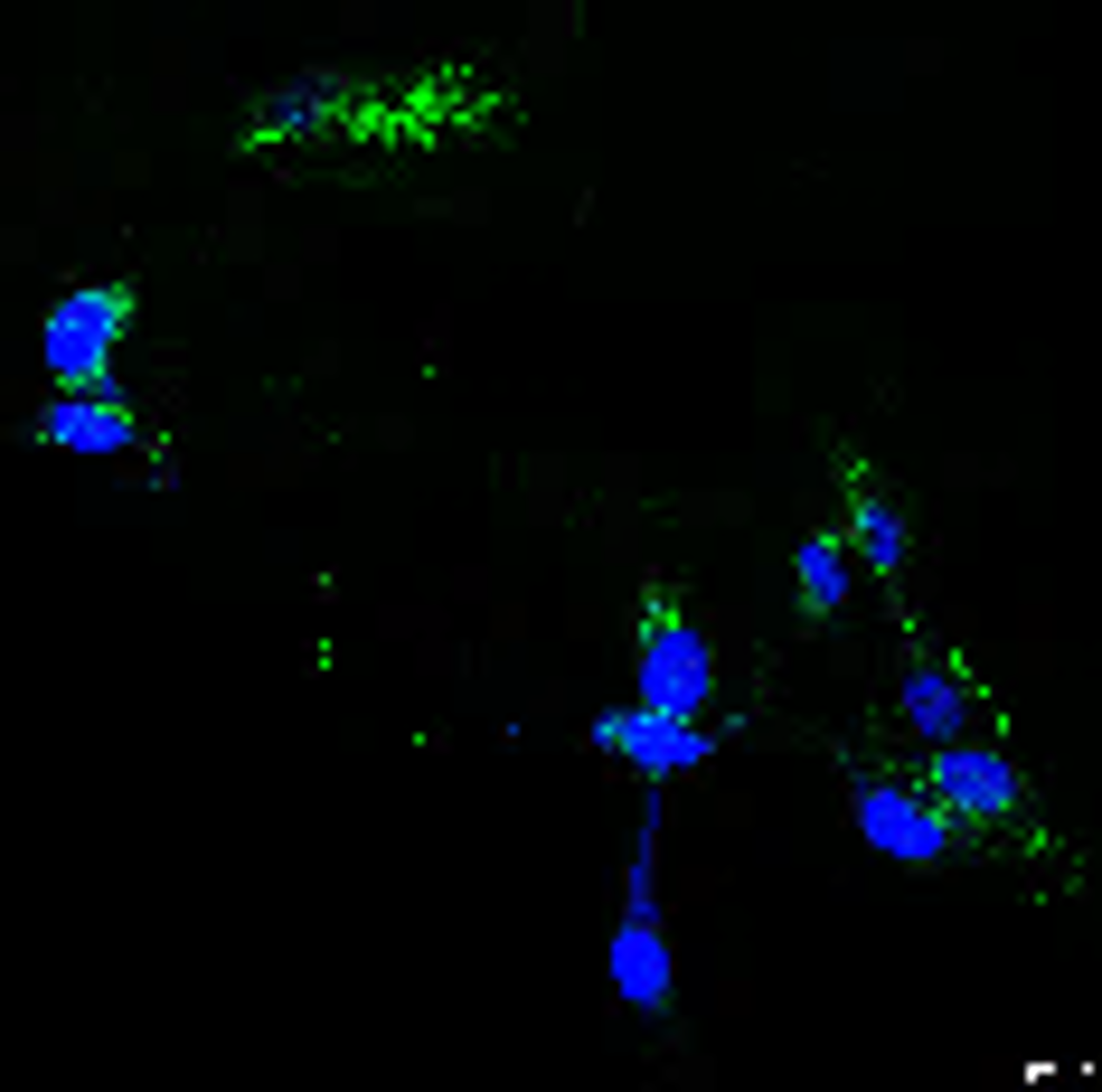

Supplement: Supplementary file 8 — Source data Fig. 2 [file 44318_2024_103_MOESM8_ESM.zip › Figure 2/2D/Fibroblasts/Small EV cultured/Day 1.tif]

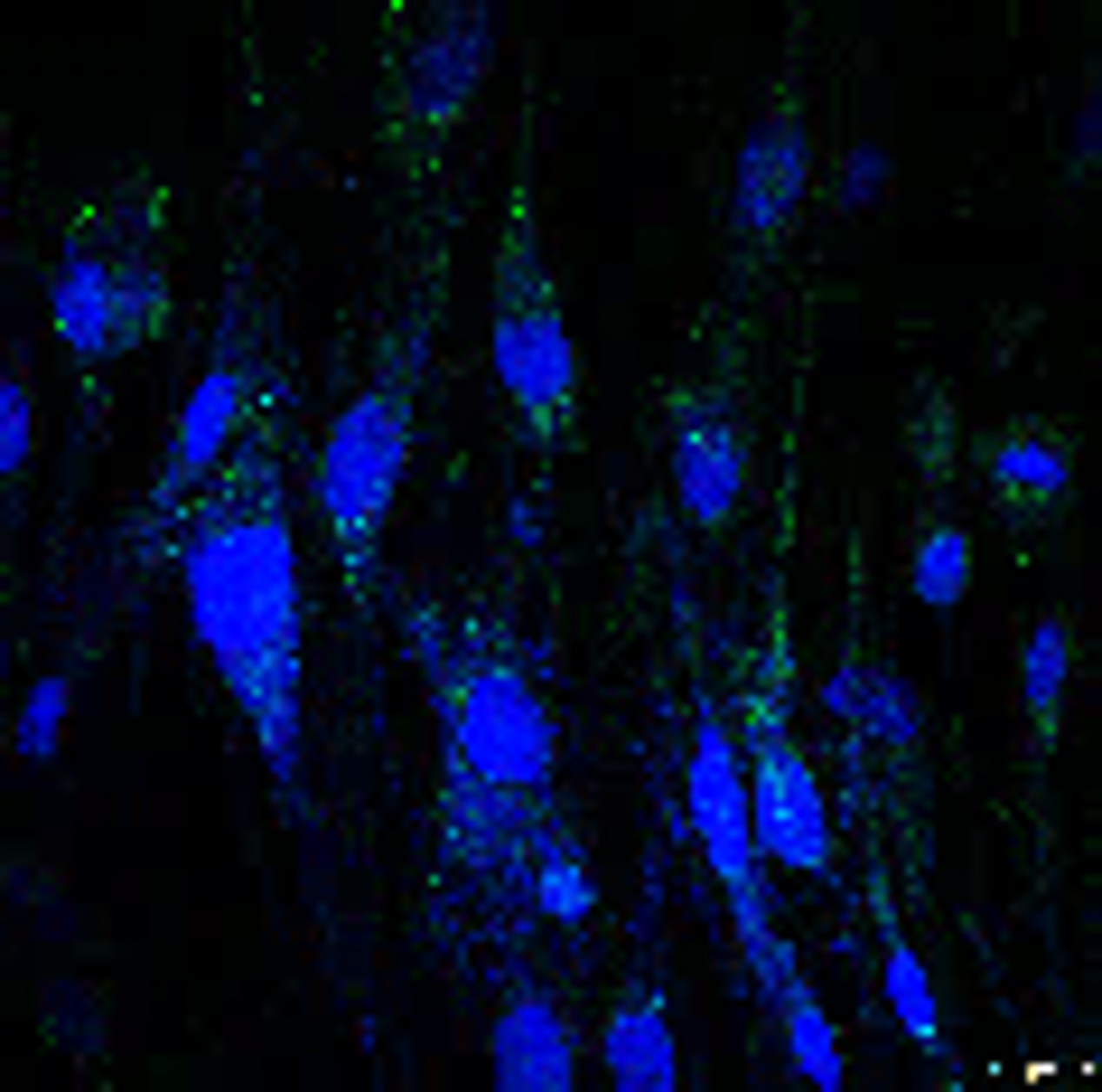

Supplement: Supplementary file 8 — Source data Fig. 2 [file 44318_2024_103_MOESM8_ESM.zip › Figure 2/2D/Fibroblasts/Small EV cultured/Day 2.tif]

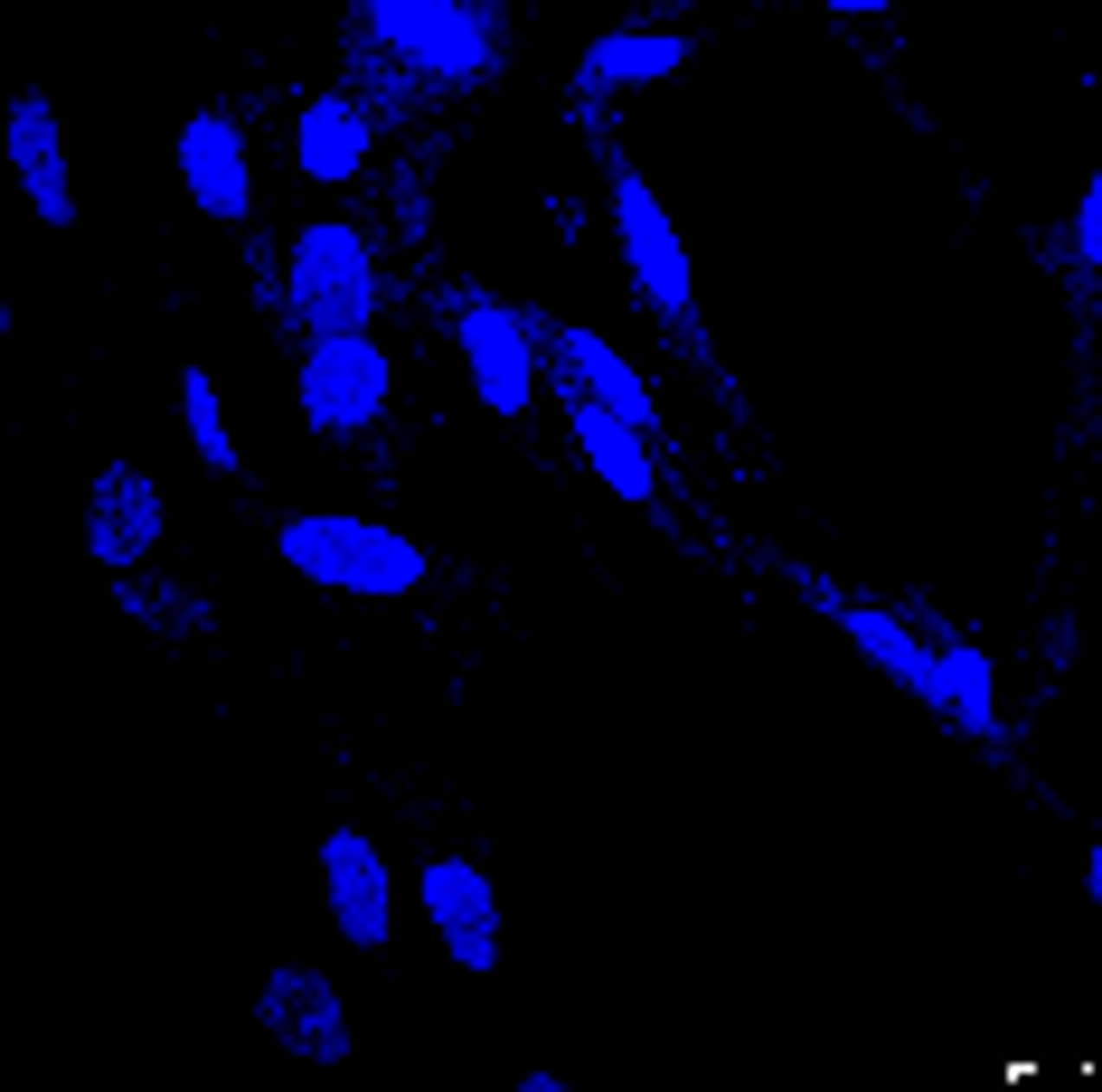

Supplement: Supplementary file 8 — Source data Fig. 2 [file 44318_2024_103_MOESM8_ESM.zip › Figure 2/2D/Fibroblasts/Small EV cultured/Day 3.tif]

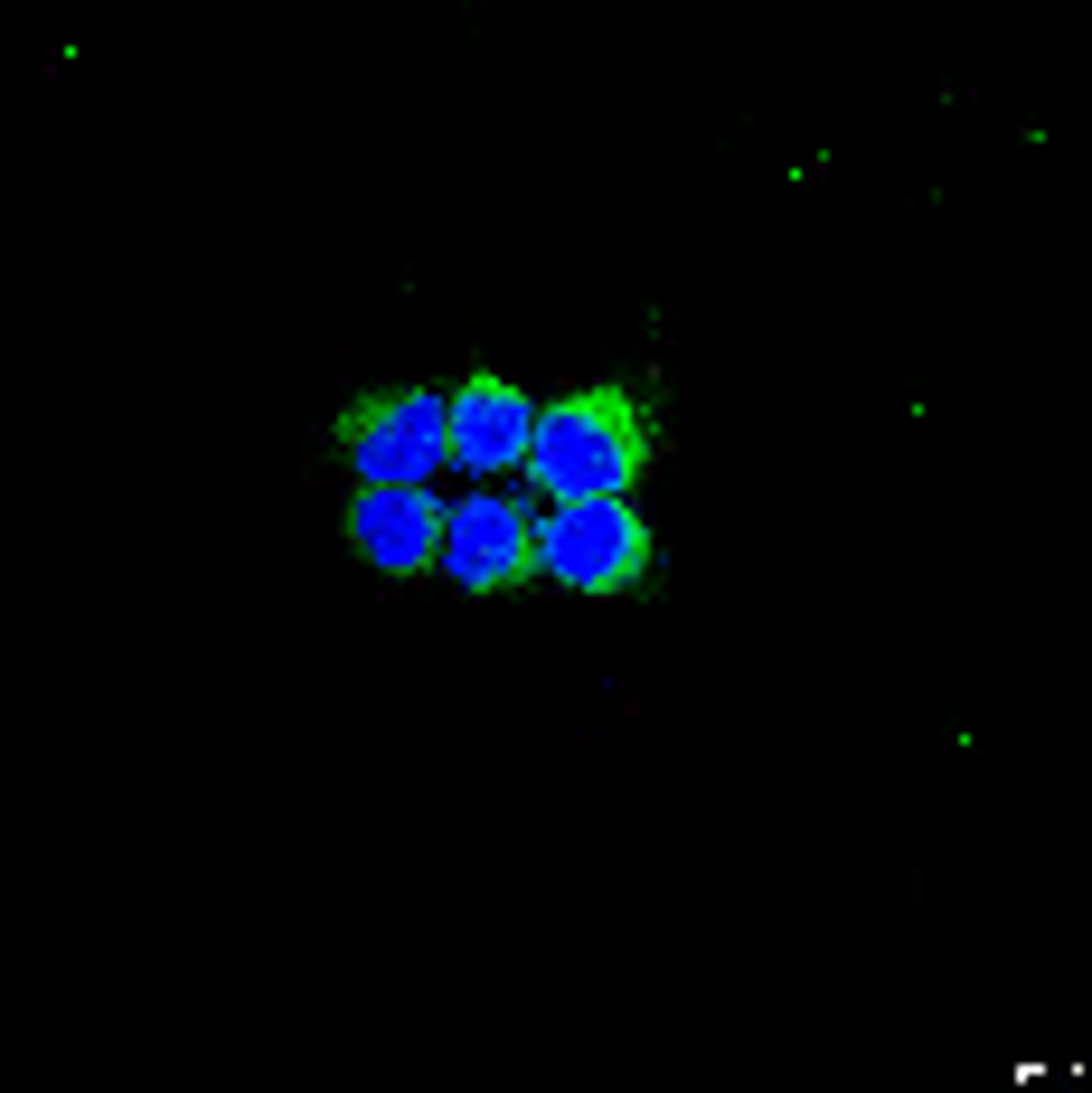

Supplement: Supplementary file 8 — Source data Fig. 2 [file 44318_2024_103_MOESM8_ESM.zip › Figure 2/2D/Keratinocytes/Melanosome cultured/Day 0.tif]

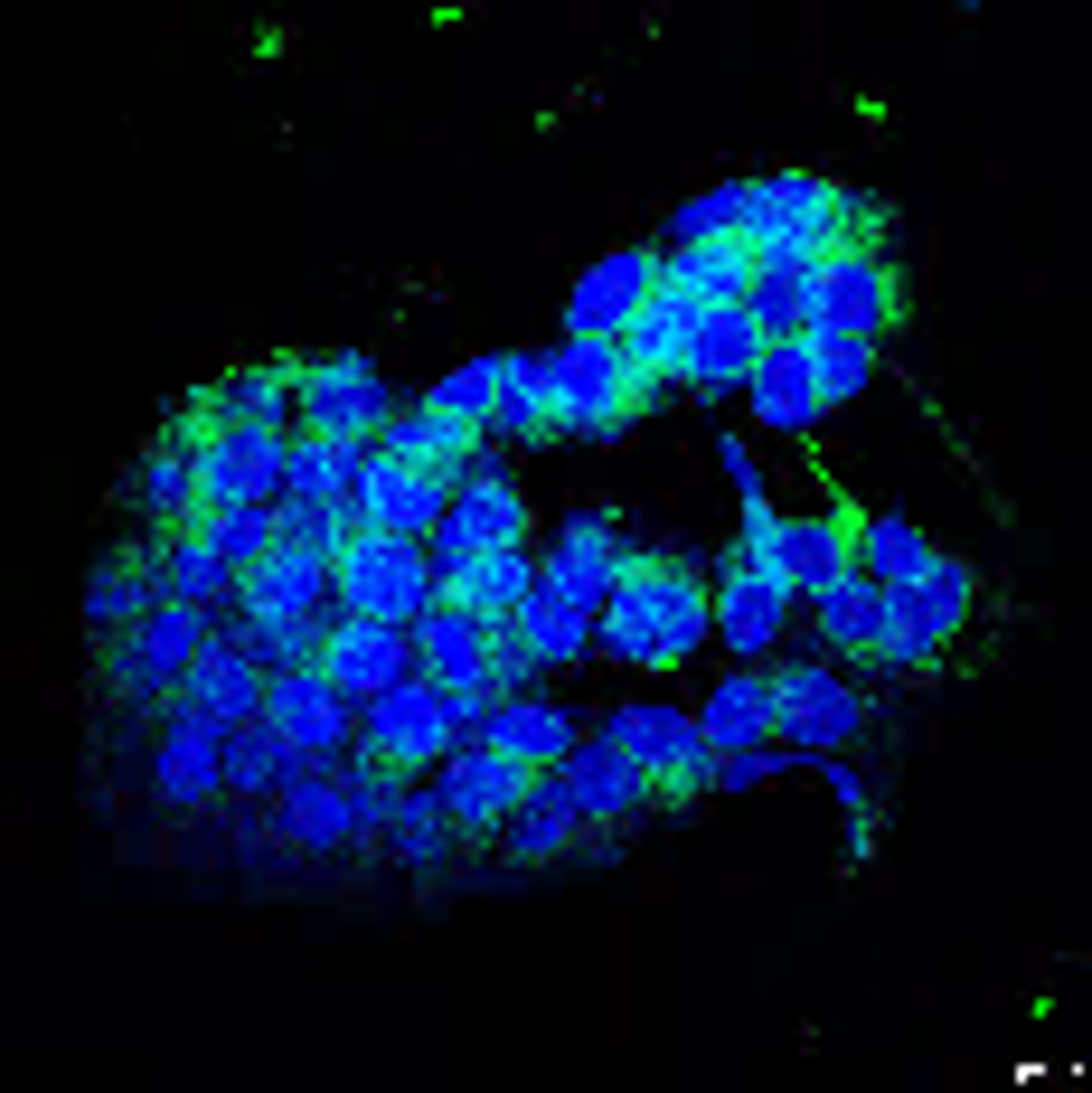

Supplement: Supplementary file 8 — Source data Fig. 2 [file 44318_2024_103_MOESM8_ESM.zip › Figure 2/2D/Keratinocytes/Melanosome cultured/Day 1.tif]

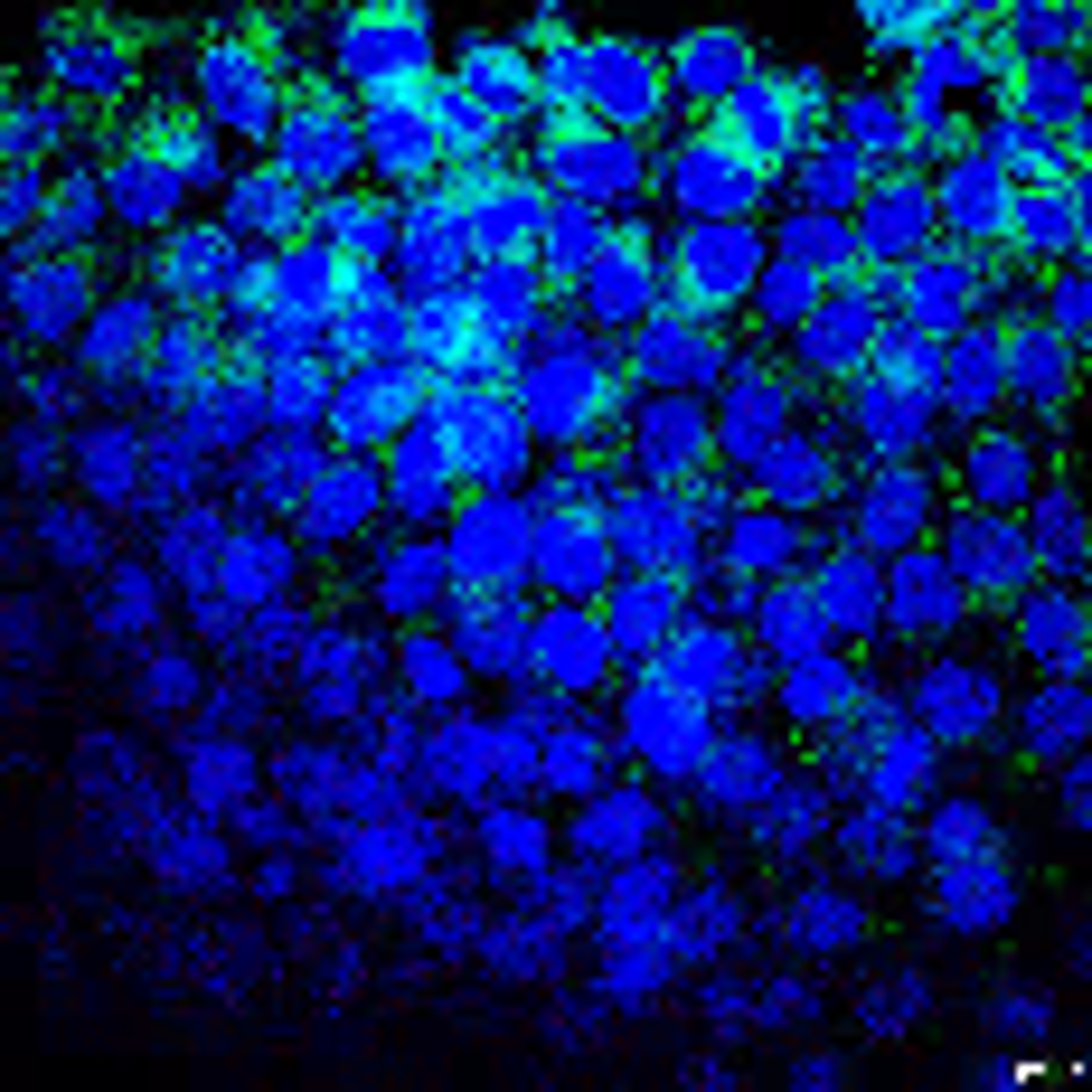

Supplement: Supplementary file 8 — Source data Fig. 2 [file 44318_2024_103_MOESM8_ESM.zip › Figure 2/2D/Keratinocytes/Melanosome cultured/Day 2.tif]

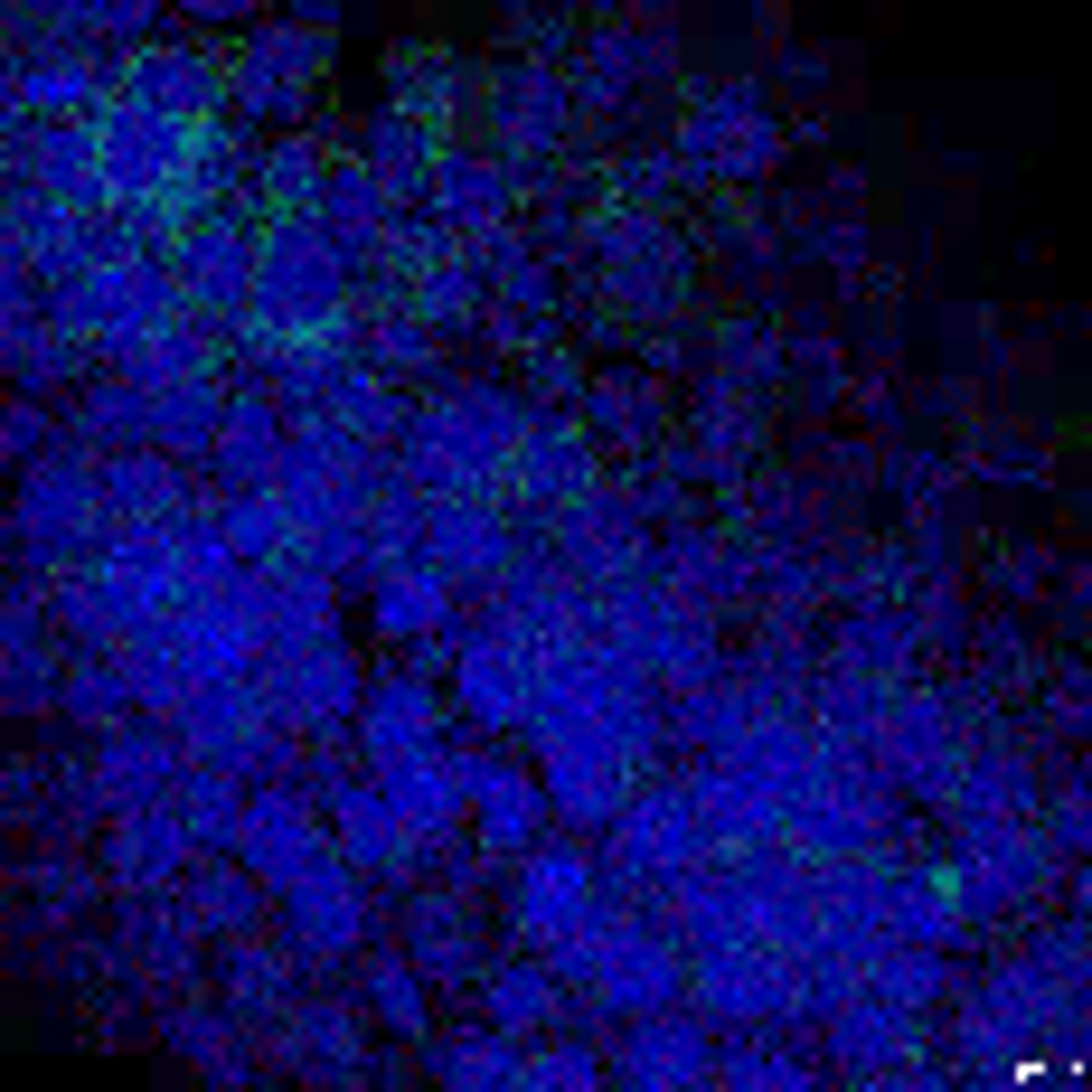

Supplement: Supplementary file 8 — Source data Fig. 2 [file 44318_2024_103_MOESM8_ESM.zip › Figure 2/2D/Keratinocytes/Melanosome cultured/Day 3.tif]

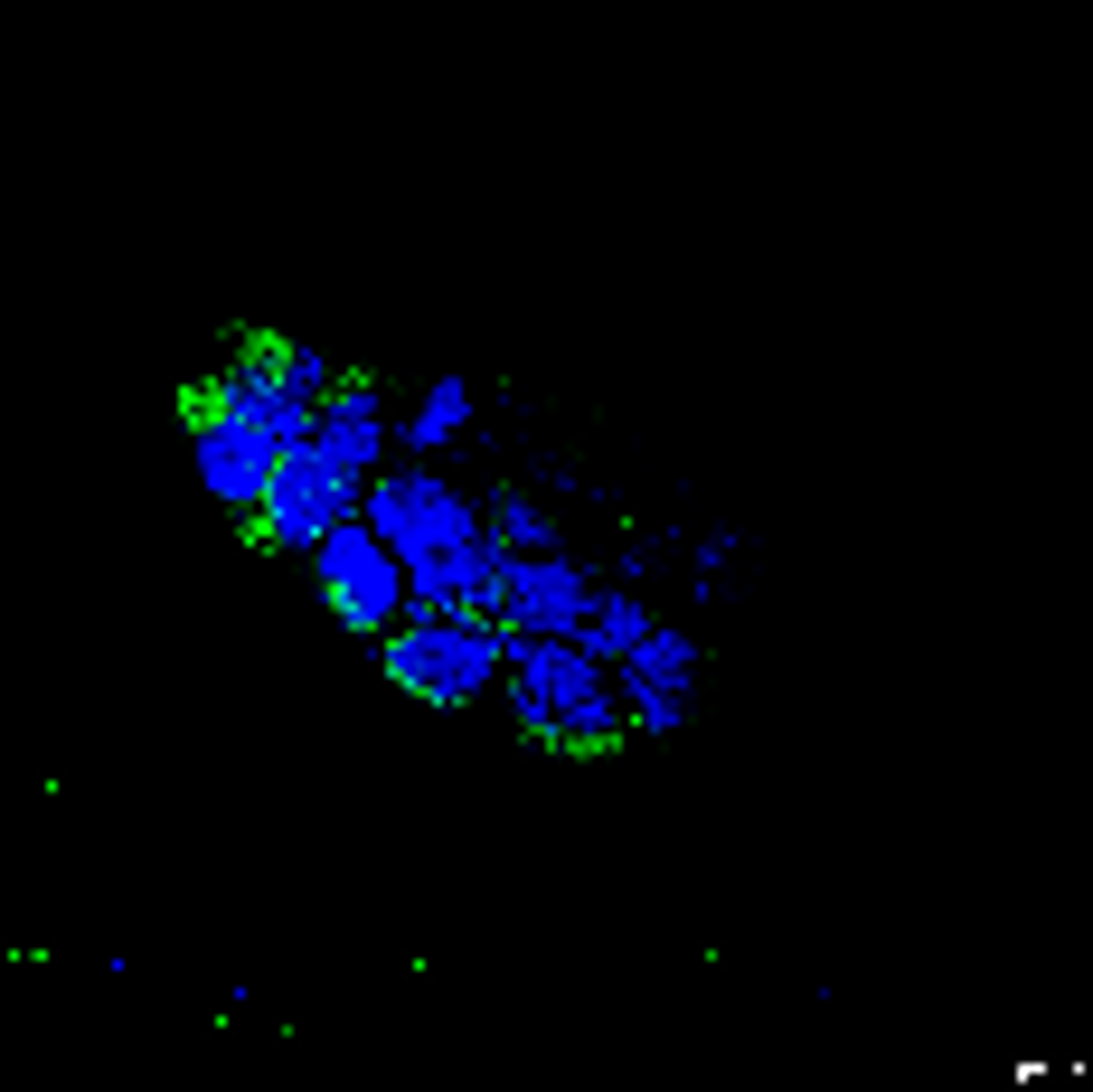

Supplement: Supplementary file 8 — Source data Fig. 2 [file 44318_2024_103_MOESM8_ESM.zip › Figure 2/2D/Keratinocytes/Small EV cultured/Day 0.tif]

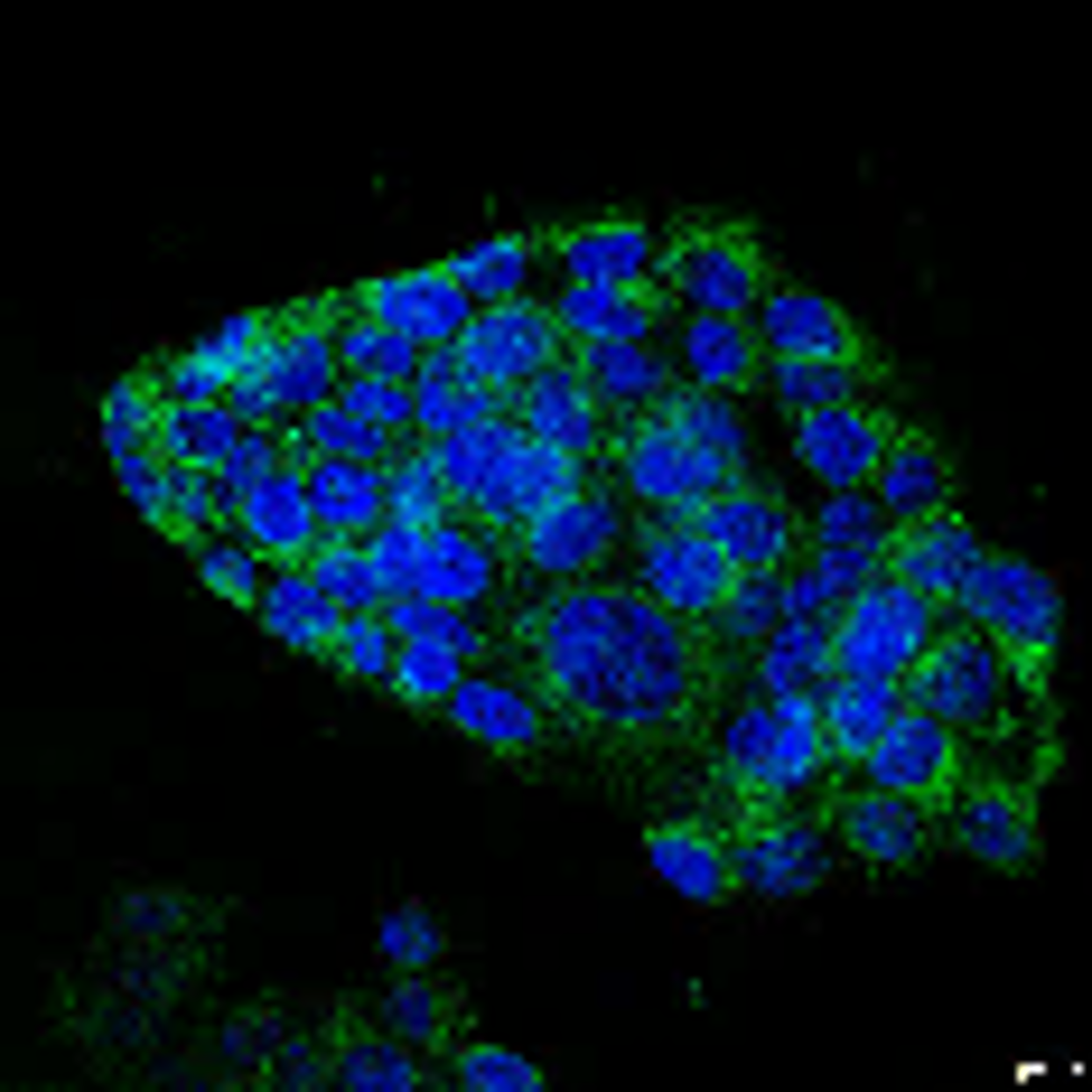

Supplement: Supplementary file 8 — Source data Fig. 2 [file 44318_2024_103_MOESM8_ESM.zip › Figure 2/2D/Keratinocytes/Small EV cultured/Day 1.tif]

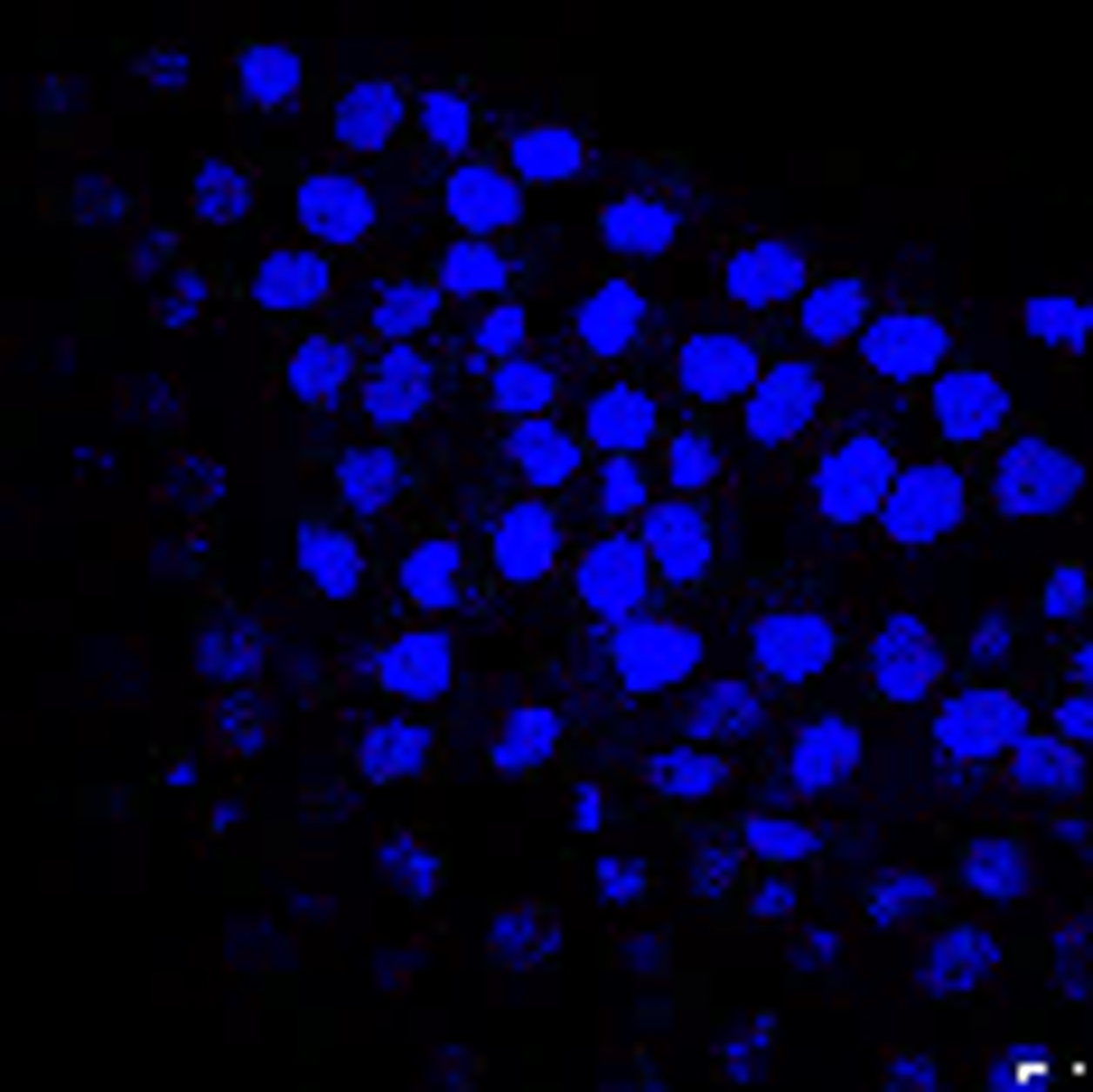

Supplement: Supplementary file 8 — Source data Fig. 2 [file 44318_2024_103_MOESM8_ESM.zip › Figure 2/2D/Keratinocytes/Small EV cultured/Day 2.tif]

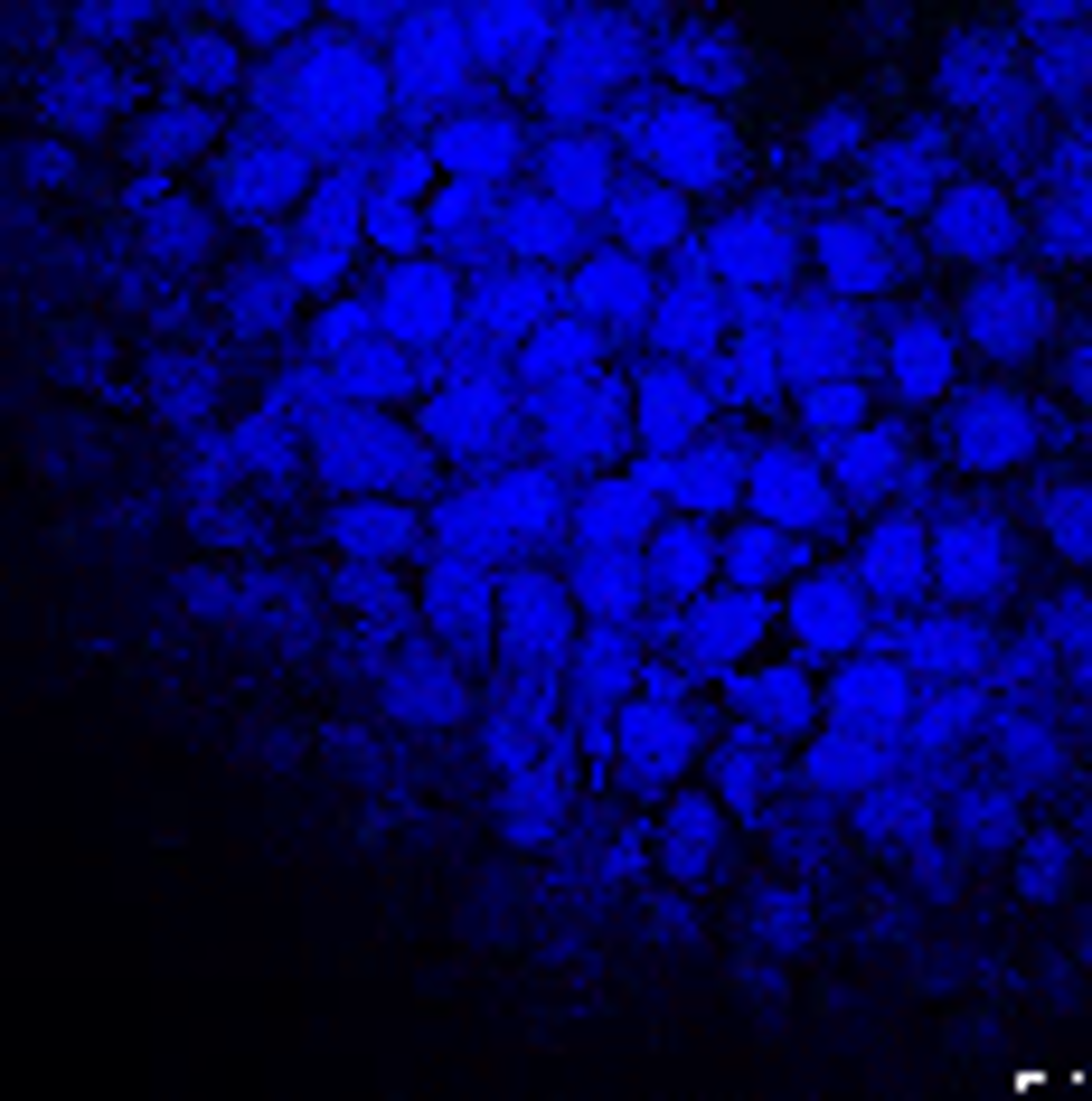

Supplement: Supplementary file 8 — Source data Fig. 2 [file 44318_2024_103_MOESM8_ESM.zip › Figure 2/2D/Keratinocytes/Small EV cultured/Day 3.tif]

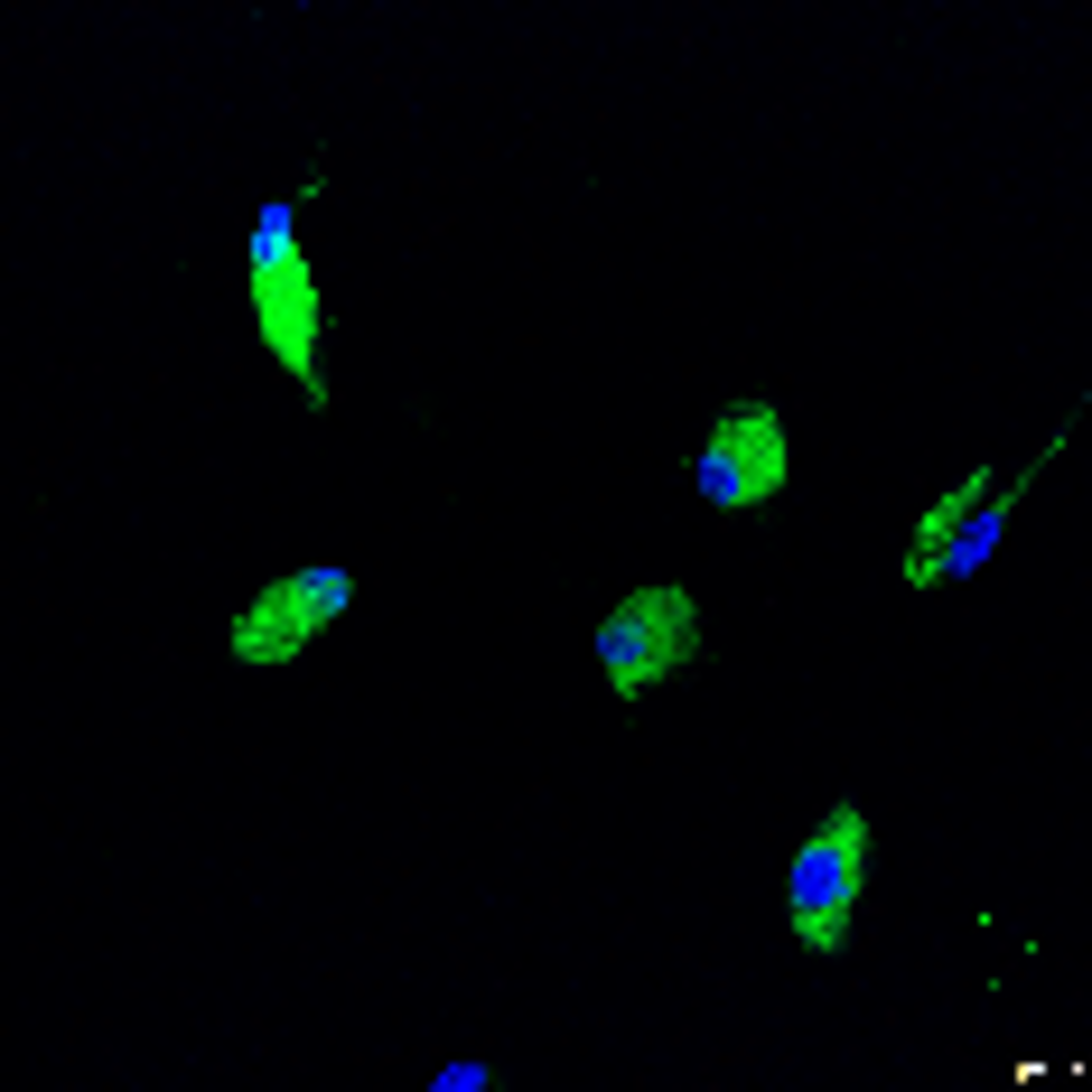

Supplement: Supplementary file 8 — Source data Fig. 2 [file 44318_2024_103_MOESM8_ESM.zip › Figure 2/2D/Macrophages/Melanosomes cultured/Day 0.tif]

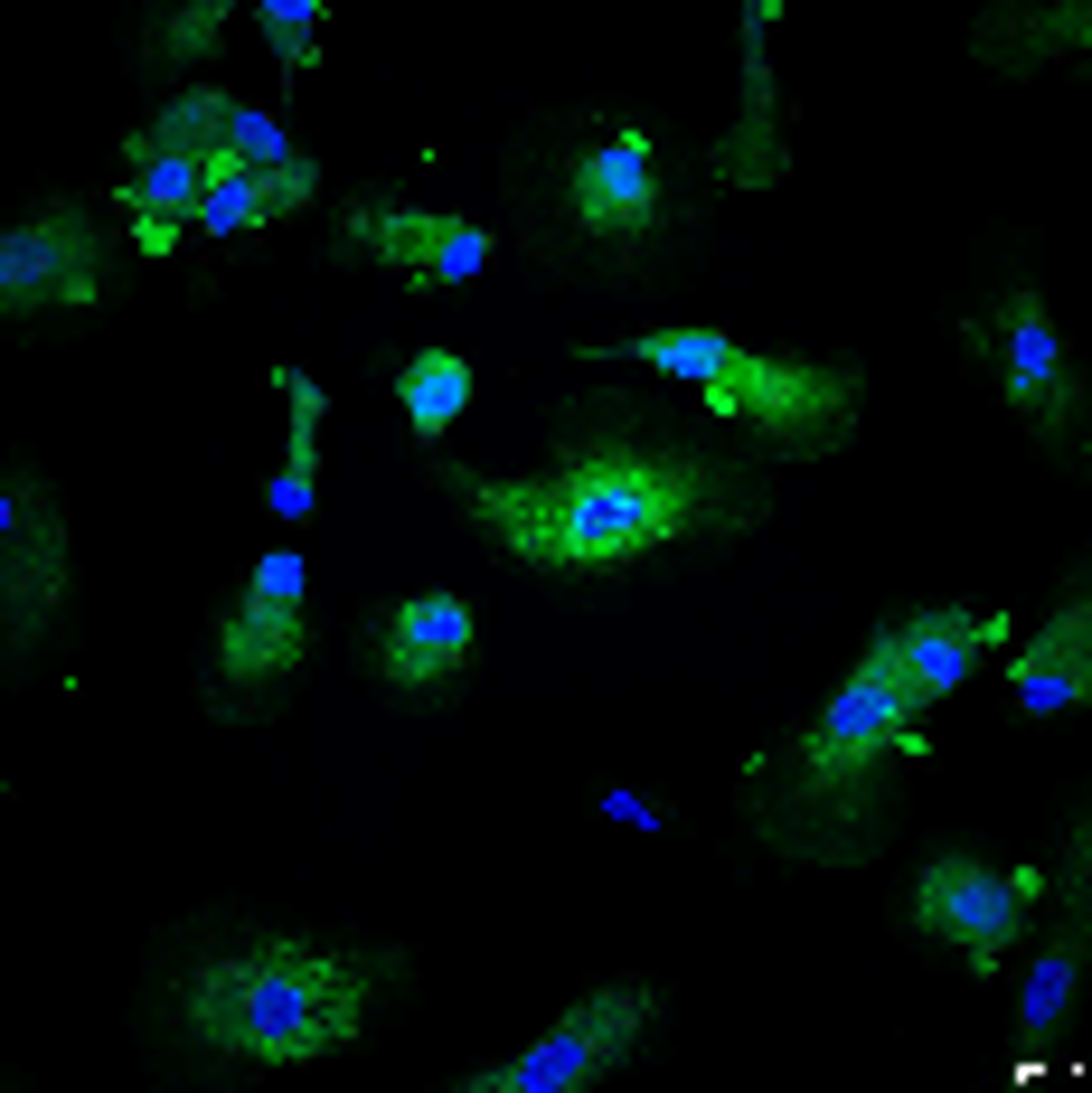

Supplement: Supplementary file 8 — Source data Fig. 2 [file 44318_2024_103_MOESM8_ESM.zip › Figure 2/2D/Macrophages/Melanosomes cultured/Day 1.tif]

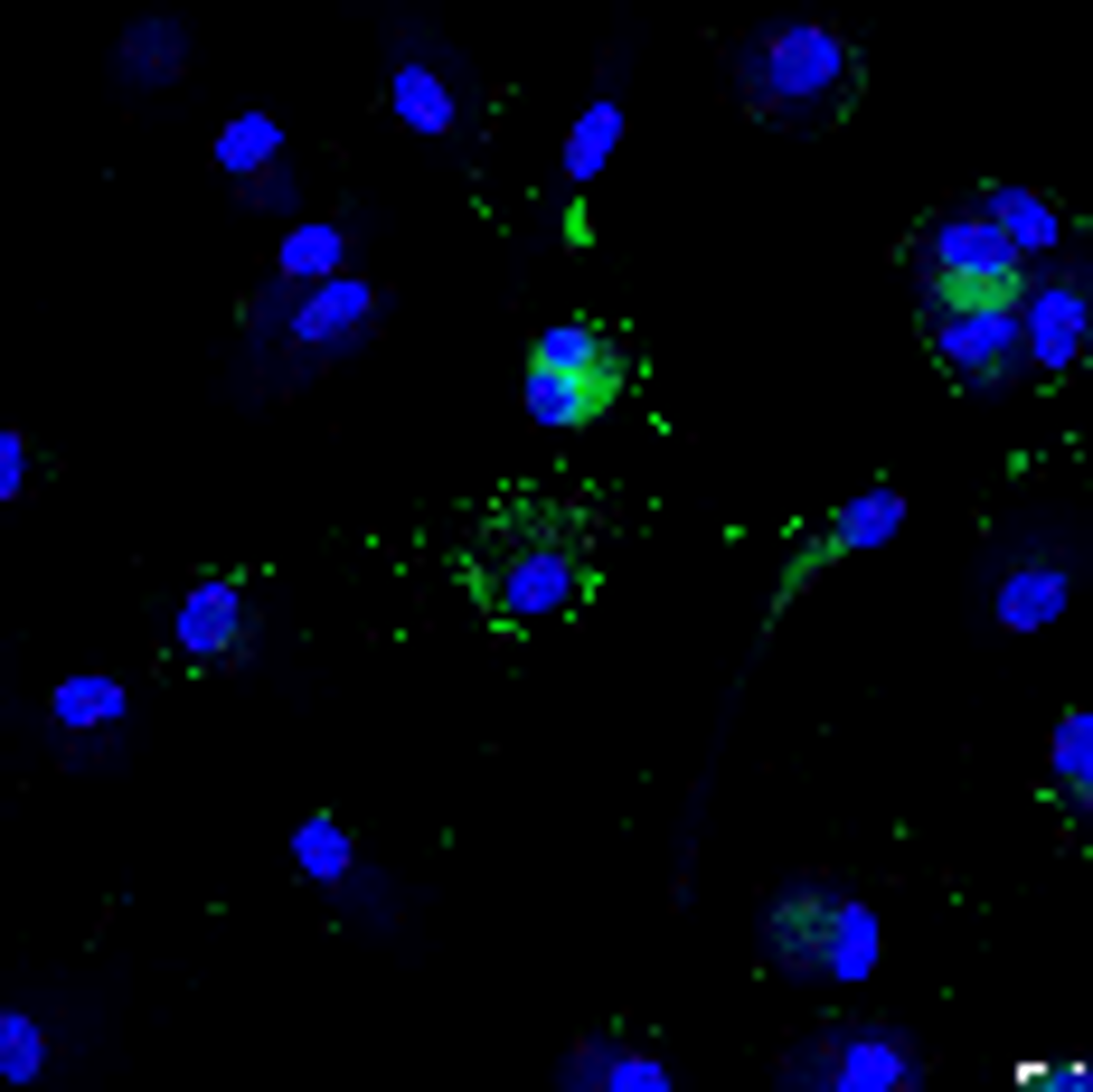

Supplement: Supplementary file 8 — Source data Fig. 2 [file 44318_2024_103_MOESM8_ESM.zip › Figure 2/2D/Macrophages/Melanosomes cultured/Day 2.tif]

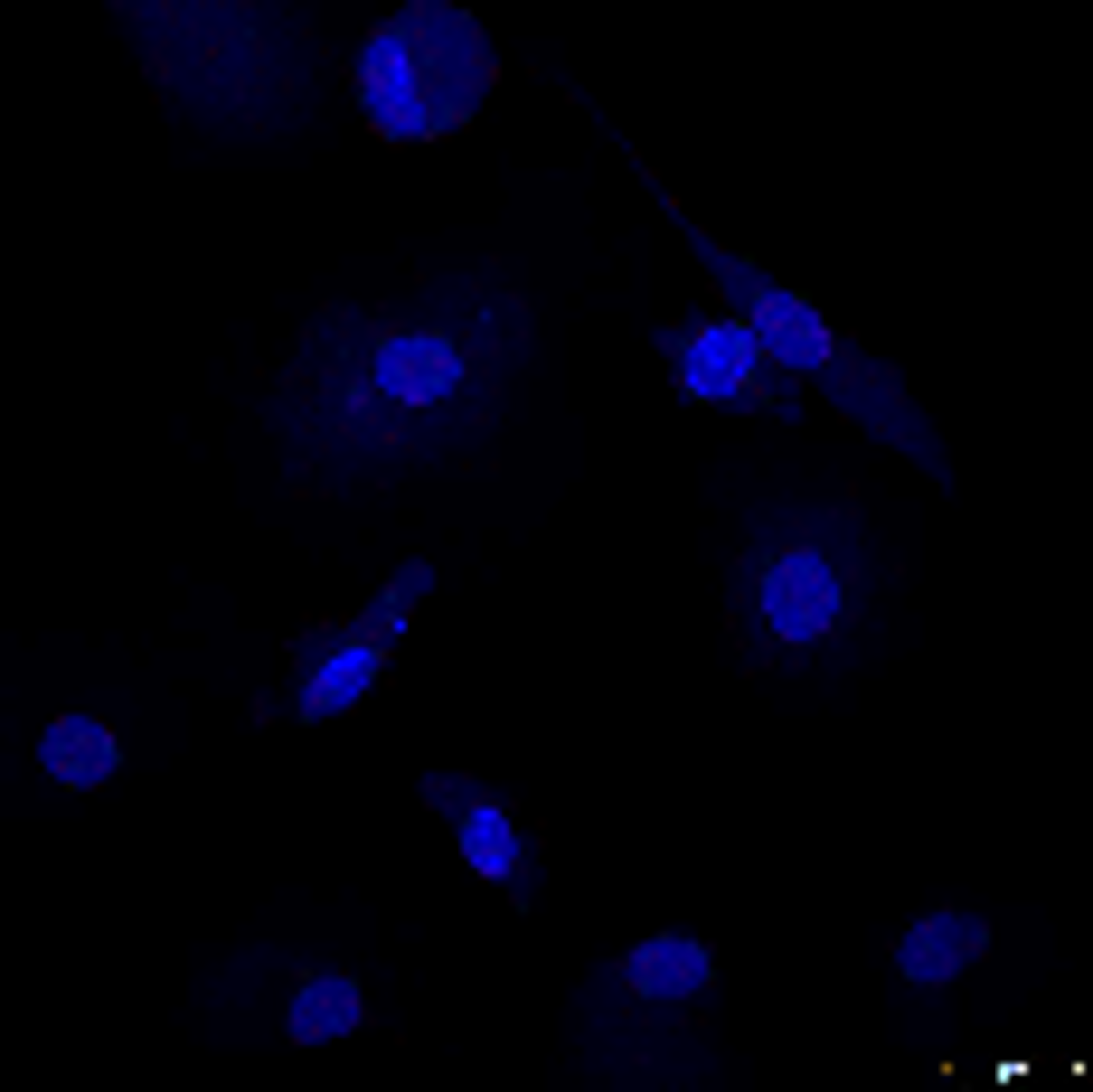

Supplement: Supplementary file 8 — Source data Fig. 2 [file 44318_2024_103_MOESM8_ESM.zip › Figure 2/2D/Macrophages/Melanosomes cultured/Day 3.tif]

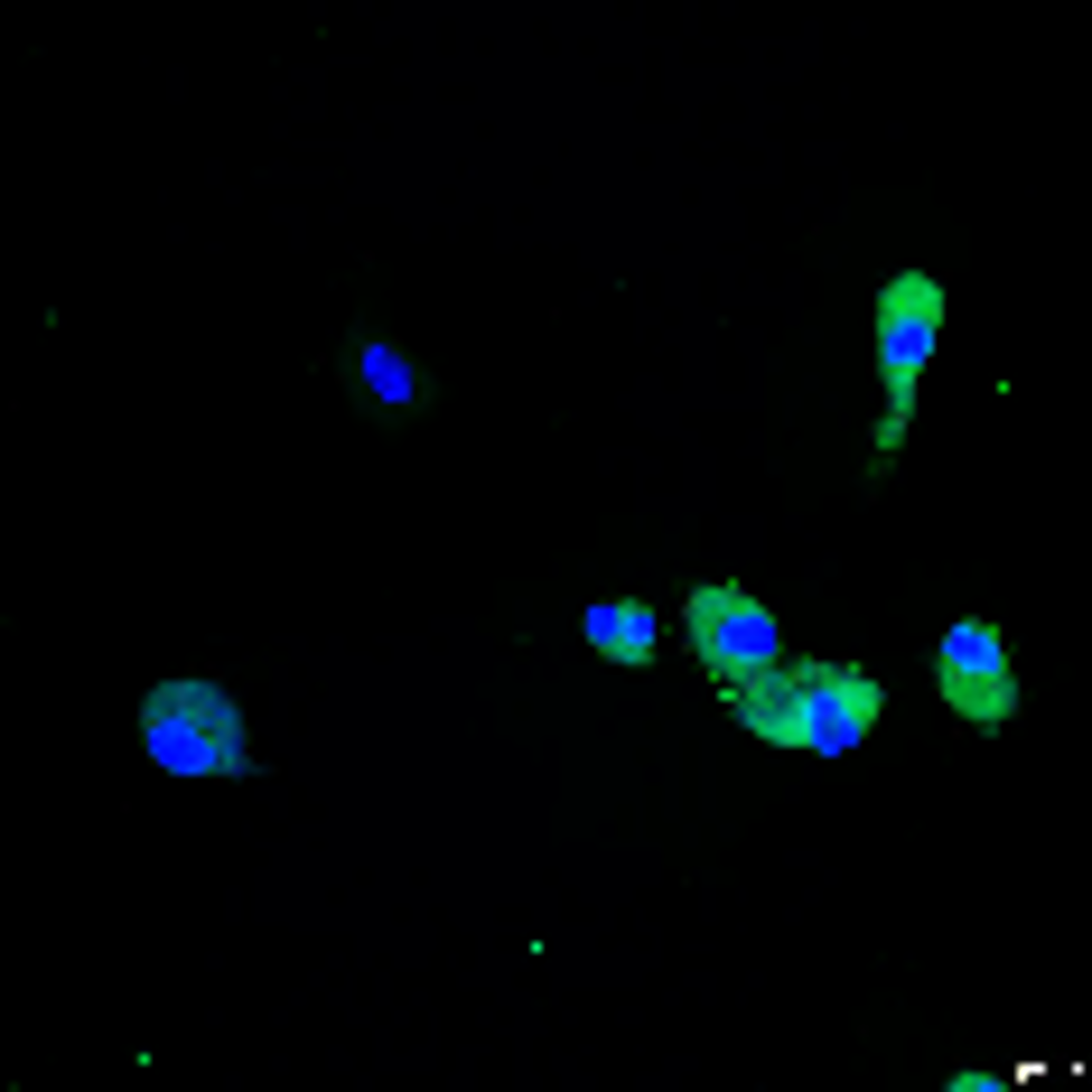

Supplement: Supplementary file 8 — Source data Fig. 2 [file 44318_2024_103_MOESM8_ESM.zip › Figure 2/2D/Macrophages/Small EV cultured/Day 0.tif]

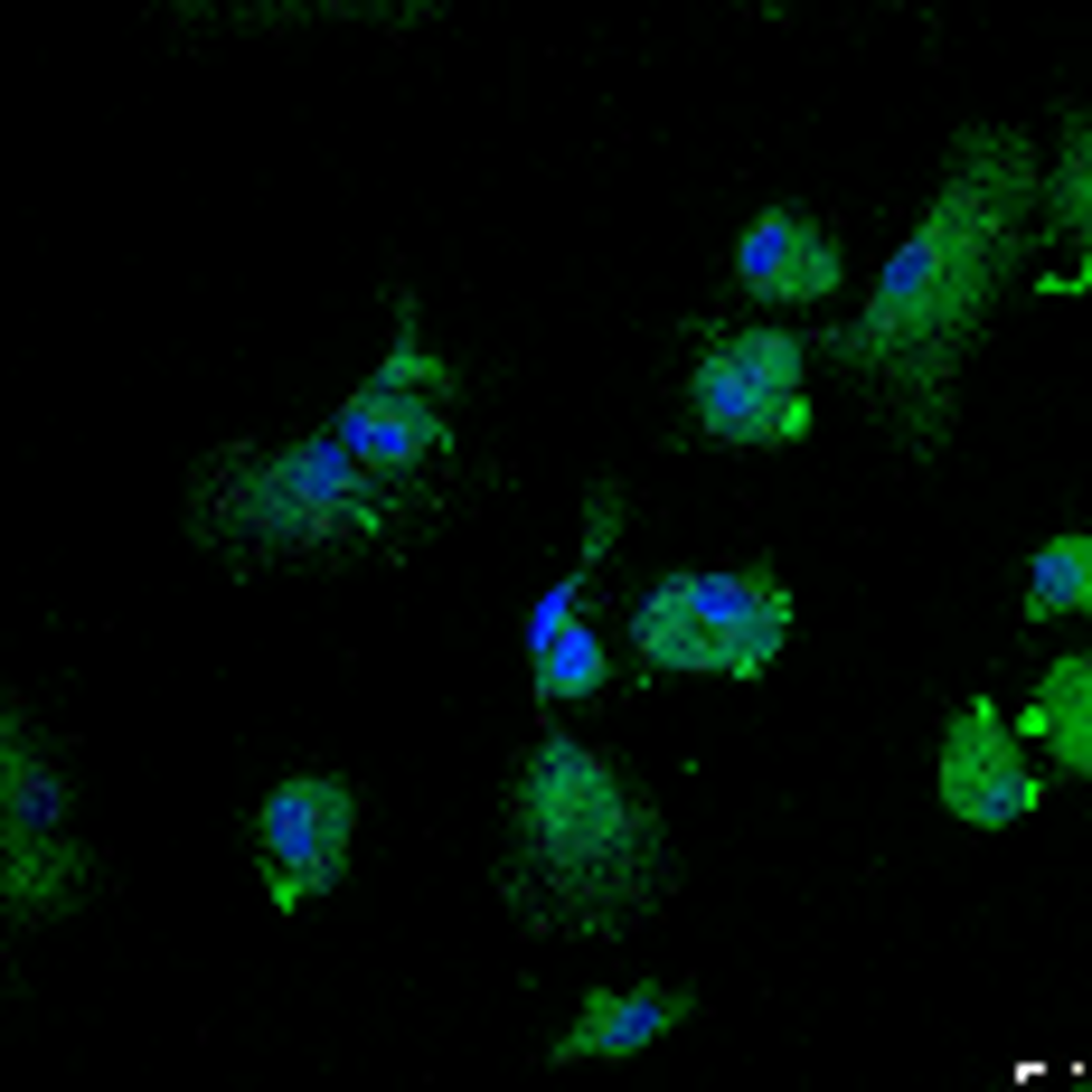

Supplement: Supplementary file 8 — Source data Fig. 2 [file 44318_2024_103_MOESM8_ESM.zip › Figure 2/2D/Macrophages/Small EV cultured/Day 1.tif]

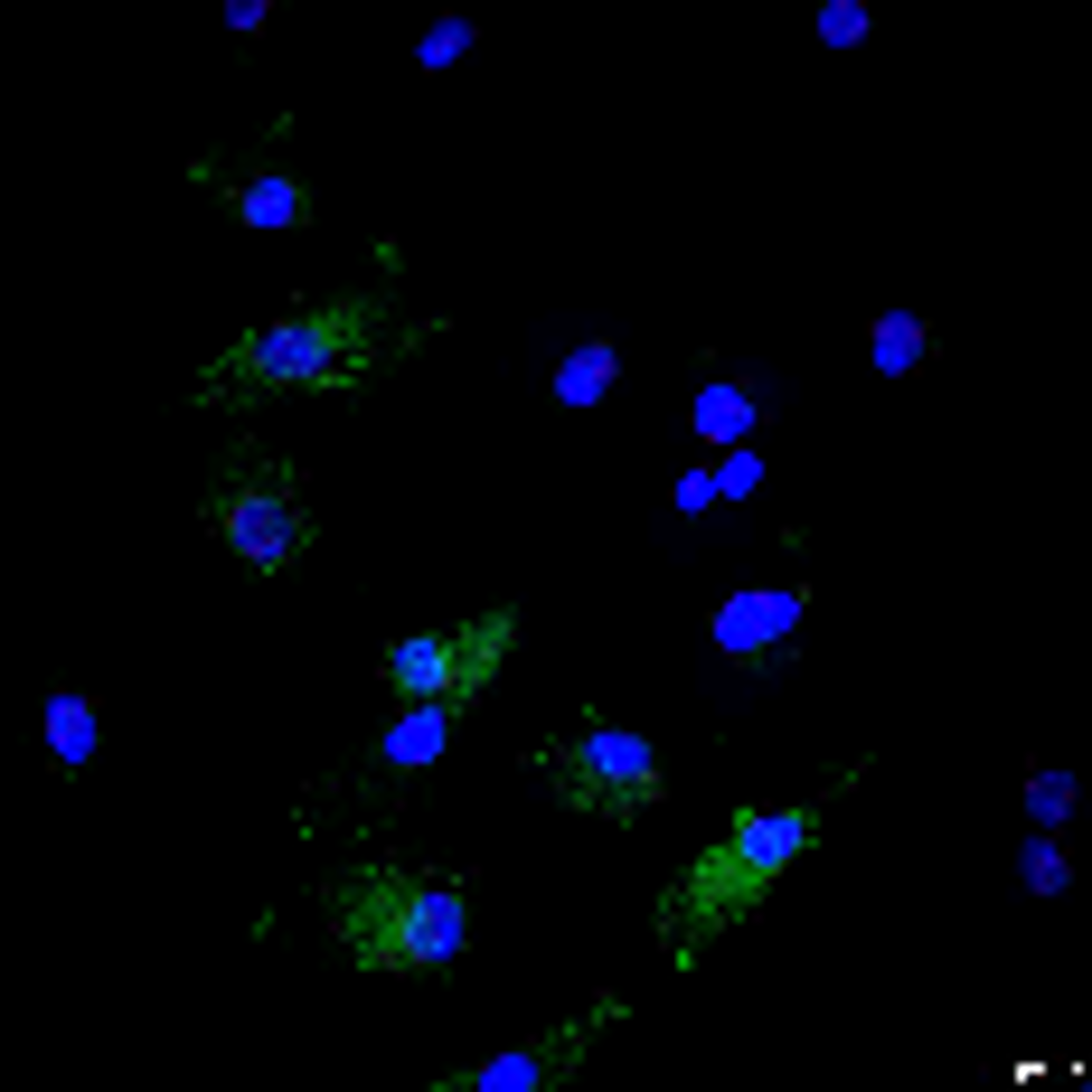

Supplement: Supplementary file 8 — Source data Fig. 2 [file 44318_2024_103_MOESM8_ESM.zip › Figure 2/2D/Macrophages/Small EV cultured/Day 2.tif]

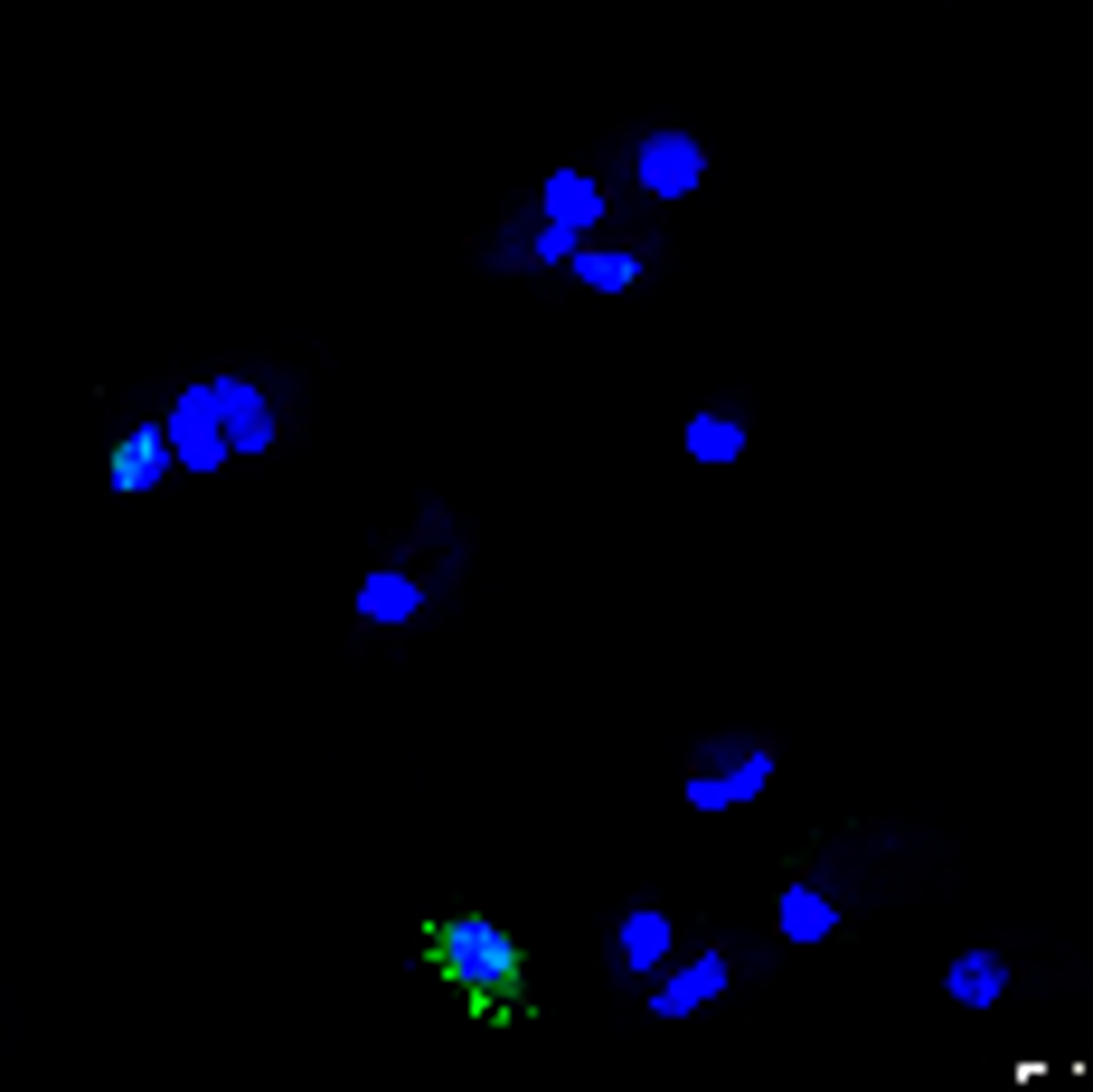

Supplement: Supplementary file 8 — Source data Fig. 2 [file 44318_2024_103_MOESM8_ESM.zip › Figure 2/2D/Macrophages/Small EV cultured/Day 3.tif]

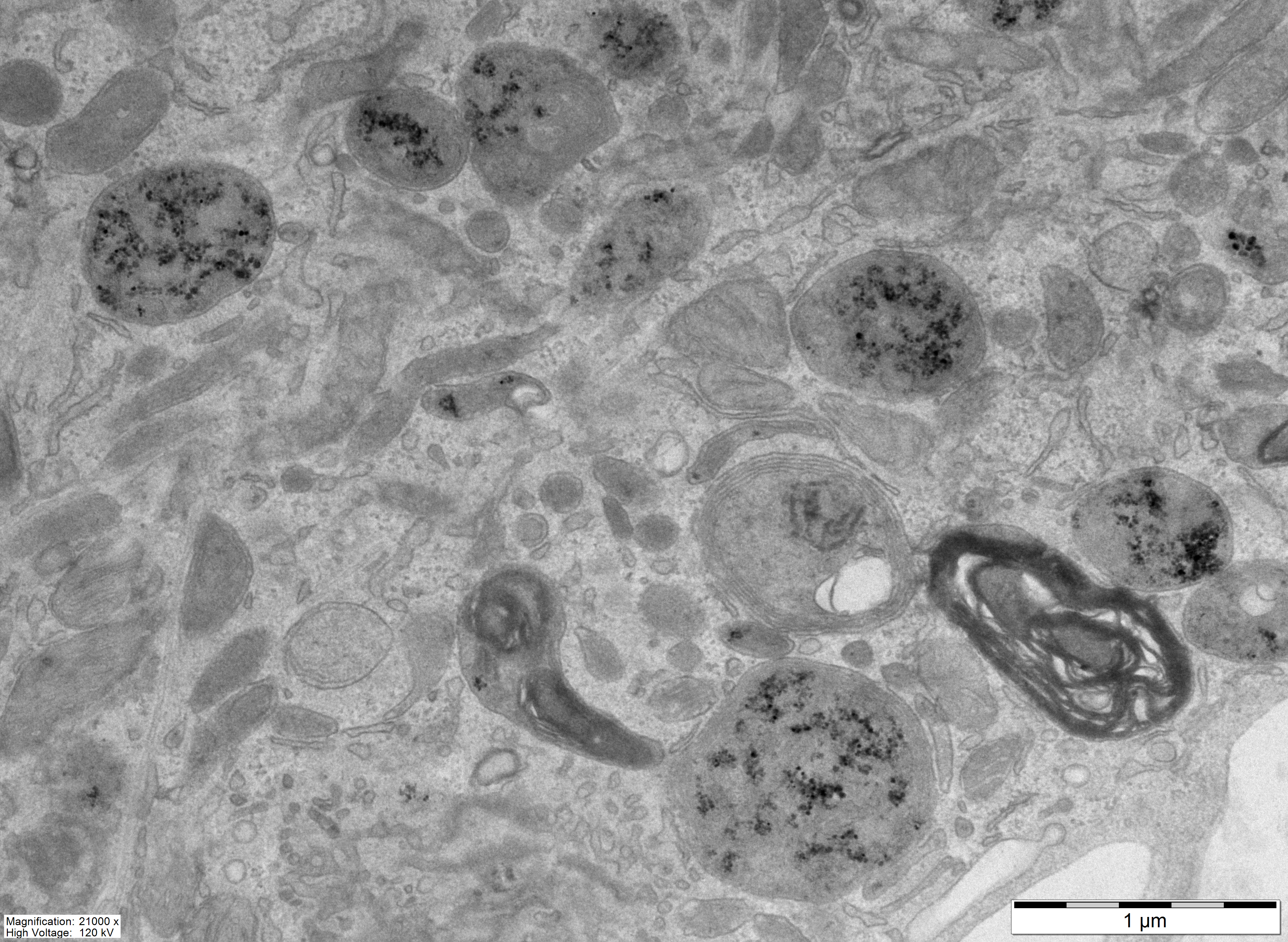

Supplement: Supplementary file 8 — Source data Fig. 2 [file 44318_2024_103_MOESM8_ESM.zip › Figure 2/2G/Macrophages with Fibroblast melanosomes/MDM+fib-90004-E2_009-18500.tif]

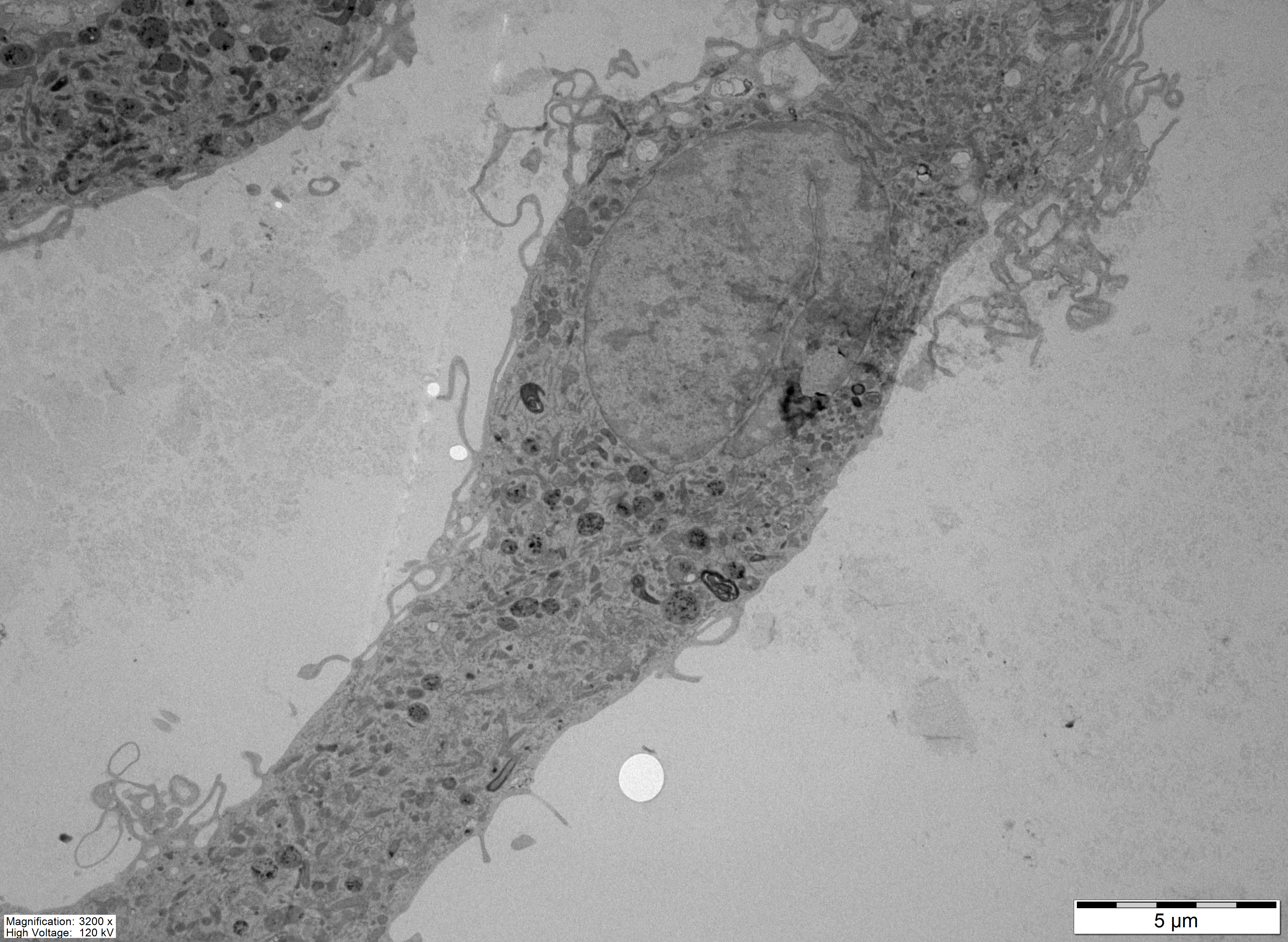

Supplement: Supplementary file 8 — Source data Fig. 2 [file 44318_2024_103_MOESM8_ESM.zip › Figure 2/2G/Macrophages with Fibroblast melanosomes/MDM+fib-90004-E2_010-2850.tif]

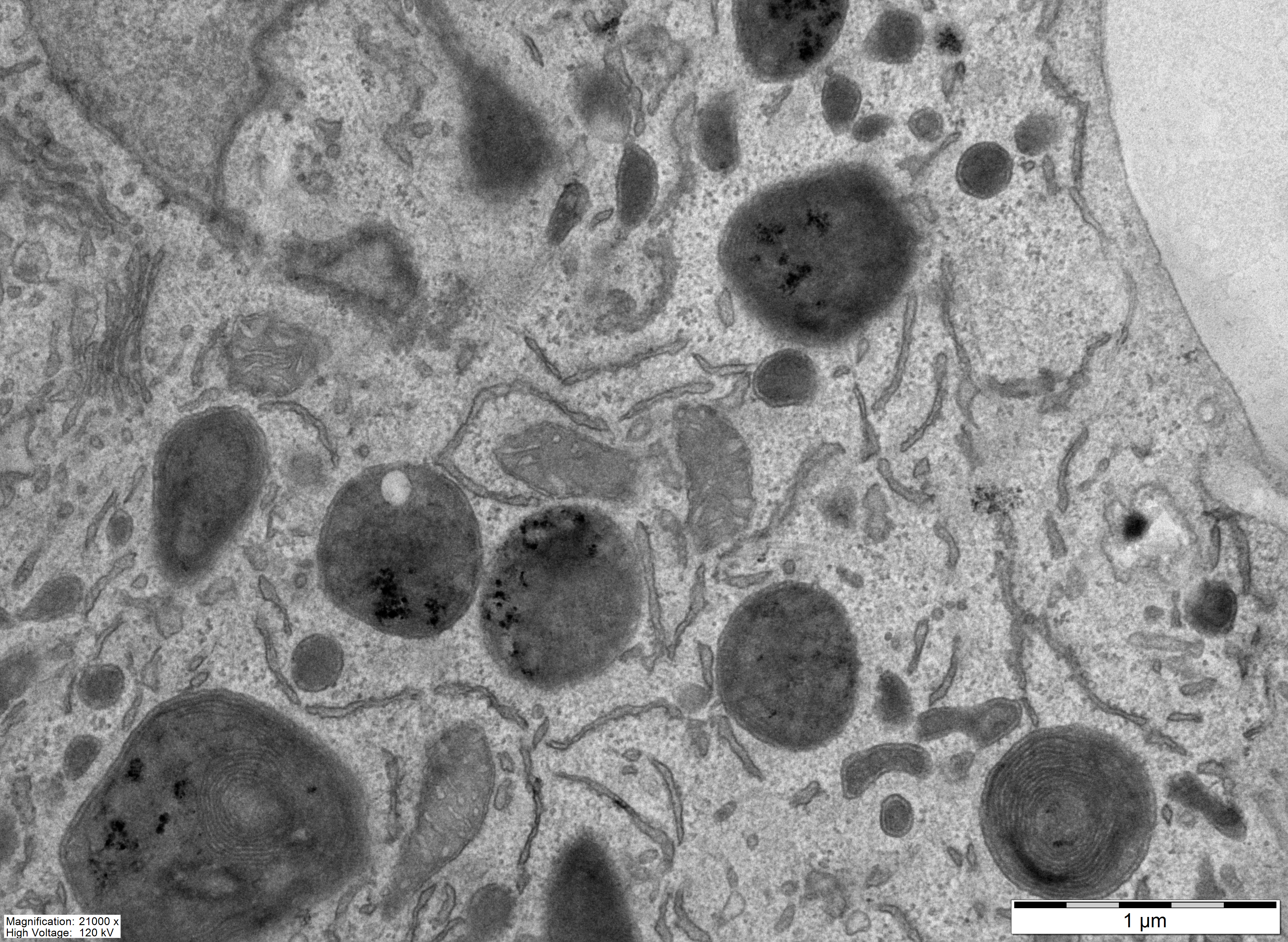

Supplement: Supplementary file 8 — Source data Fig. 2 [file 44318_2024_103_MOESM8_ESM.zip › Figure 2/2G/Macrophages with Keratinocyte melanosomes/MDM+HaCAT-90004-D2_001-18500.tif]

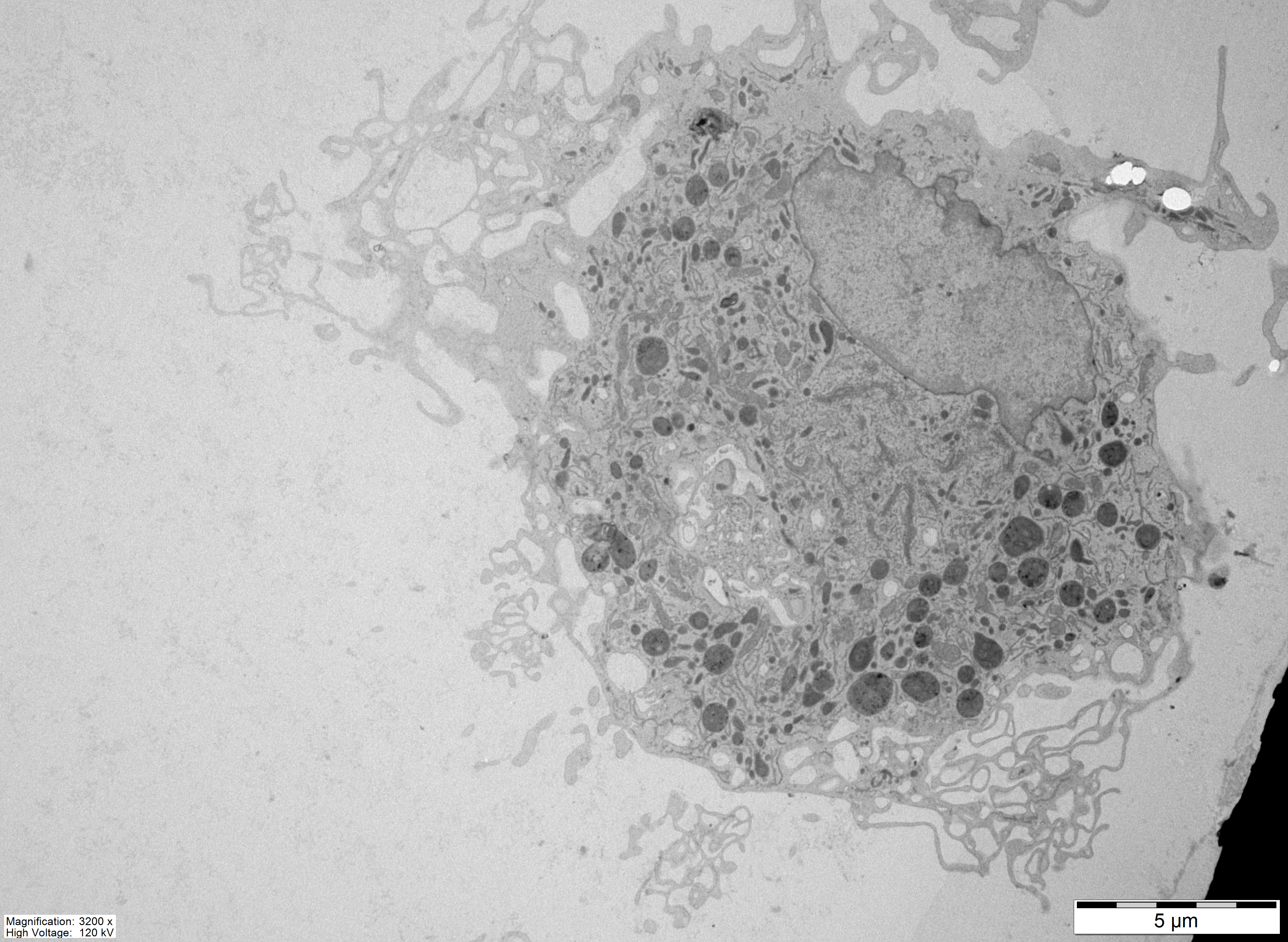

Supplement: Supplementary file 8 — Source data Fig. 2 [file 44318_2024_103_MOESM8_ESM.zip › Figure 2/2G/Macrophages with Keratinocyte melanosomes/MDM+HaCAT-90004-D2_002-2850.tif]

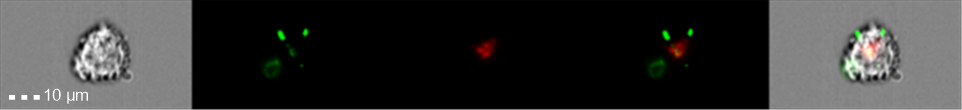

Supplement: Supplementary file 8 — Source data Fig. 2 [file 44318_2024_103_MOESM8_ESM.zip › Figure 2/2H/Fib. cells with MNT1 melanosomes.tif]

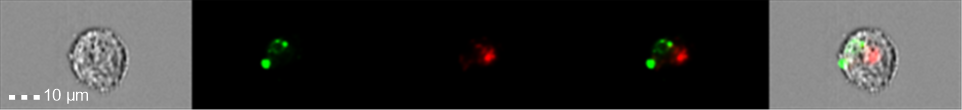

Supplement: Supplementary file 8 — Source data Fig. 2 [file 44318_2024_103_MOESM8_ESM.zip › Figure 2/2H/Ker. cells with MNT1 melanosomes.tif]

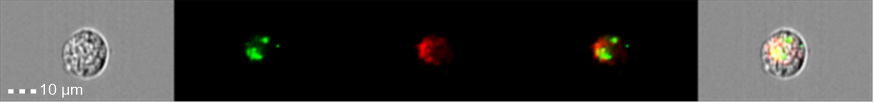

Supplement: Supplementary file 8 — Source data Fig. 2 [file 44318_2024_103_MOESM8_ESM.zip › Figure 2/2H/Macrophages with Fib. melanosomes.tif]

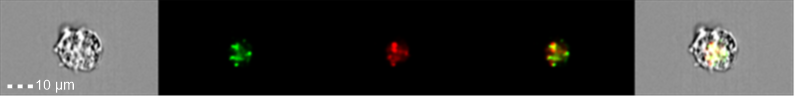

Supplement: Supplementary file 8 — Source data Fig. 2 [file 44318_2024_103_MOESM8_ESM.zip › Figure 2/2H/Macrophages with Ker. melanosomes.tif]

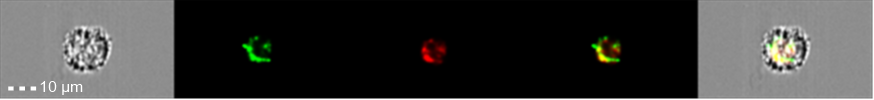

Supplement: Supplementary file 8 — Source data Fig. 2 [file 44318_2024_103_MOESM8_ESM.zip › Figure 2/2H/Macrophages with MNT1 melanosomes.tif]

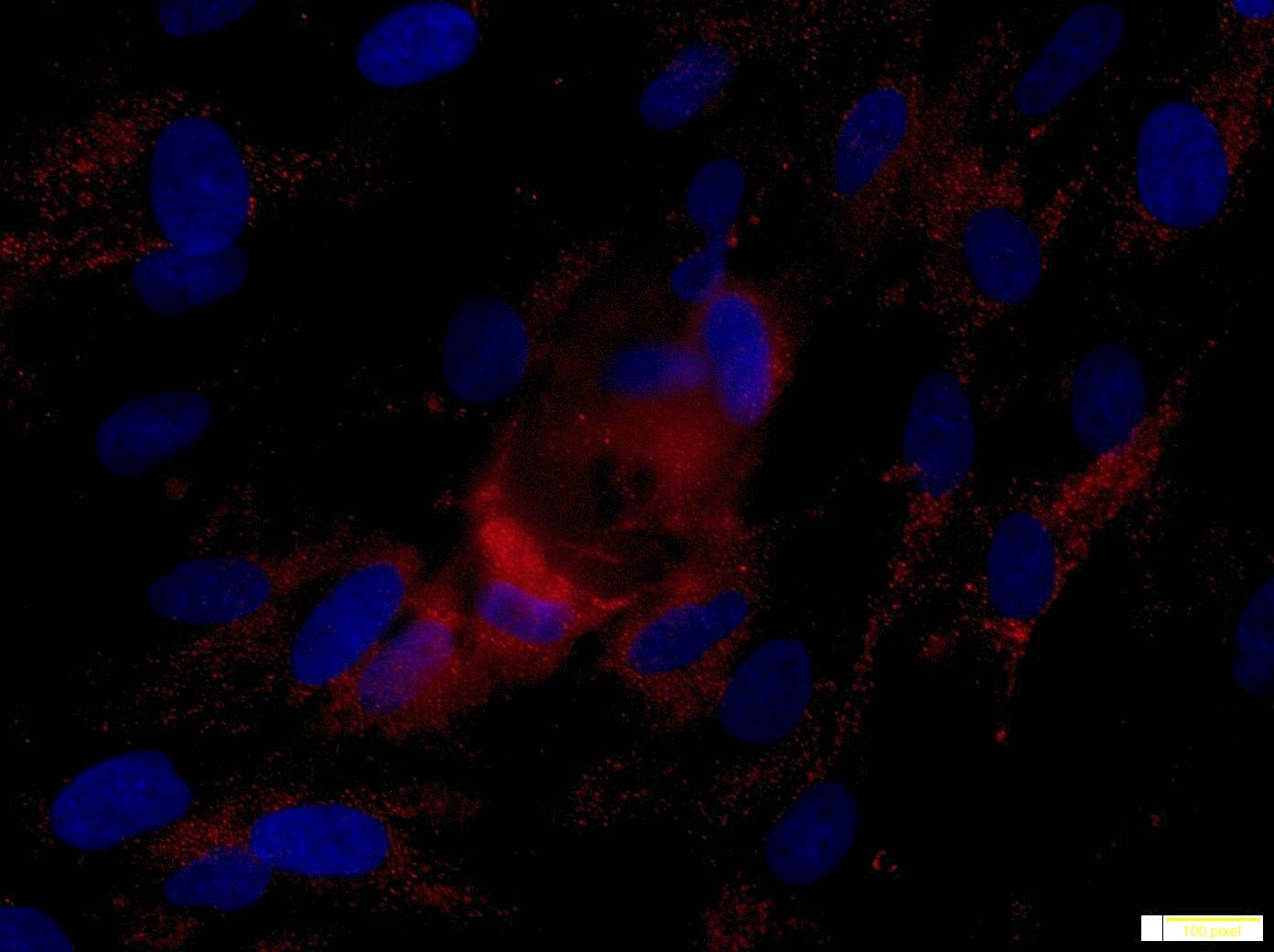

Supplement: Supplementary file 8 — Source data Fig. 2 [file 44318_2024_103_MOESM8_ESM.zip › Figure 2/2I/Fibroblasts.jpg]

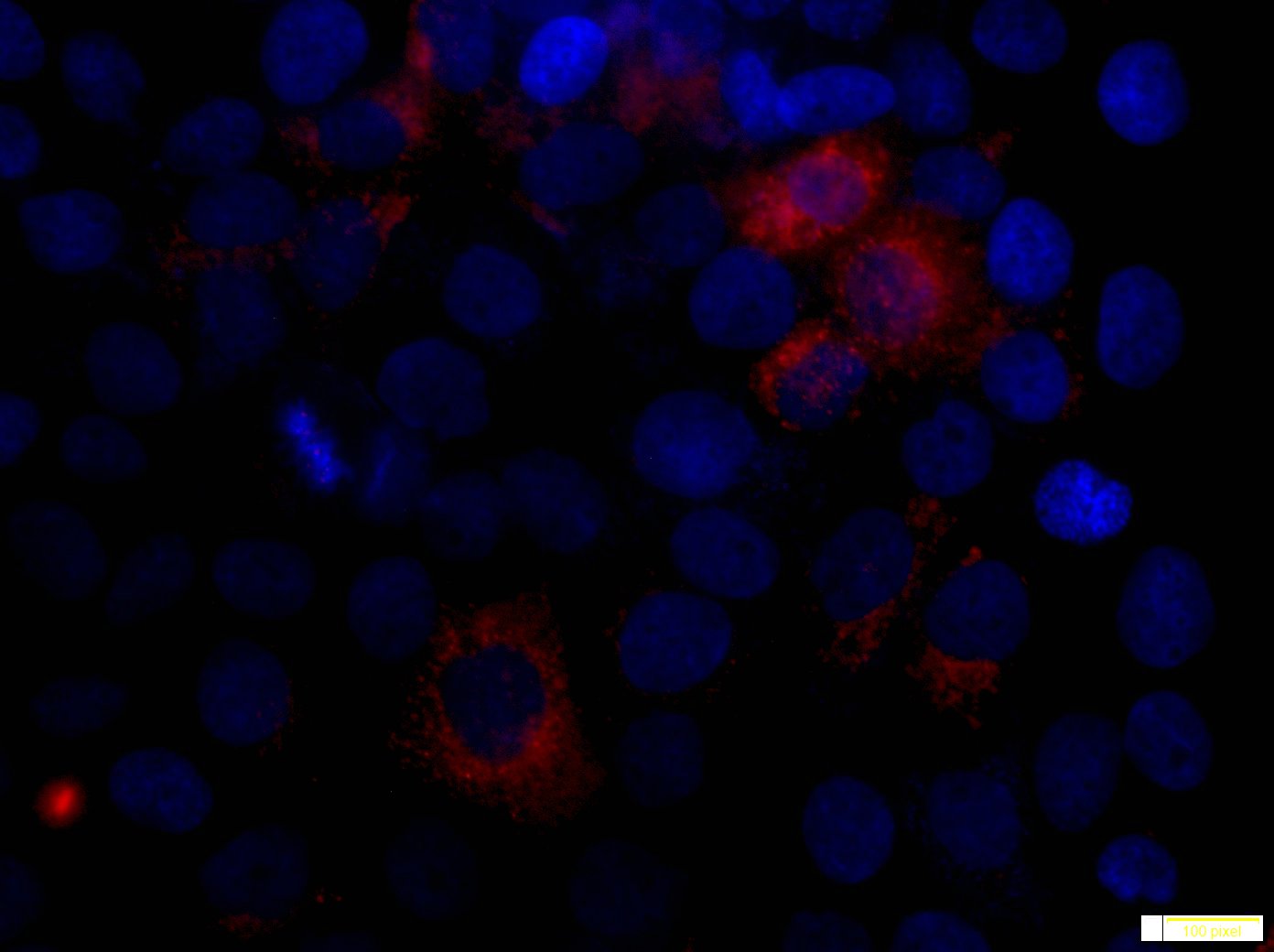

Supplement: Supplementary file 8 — Source data Fig. 2 [file 44318_2024_103_MOESM8_ESM.zip › Figure 2/2I/Keratinocytes.jpg]

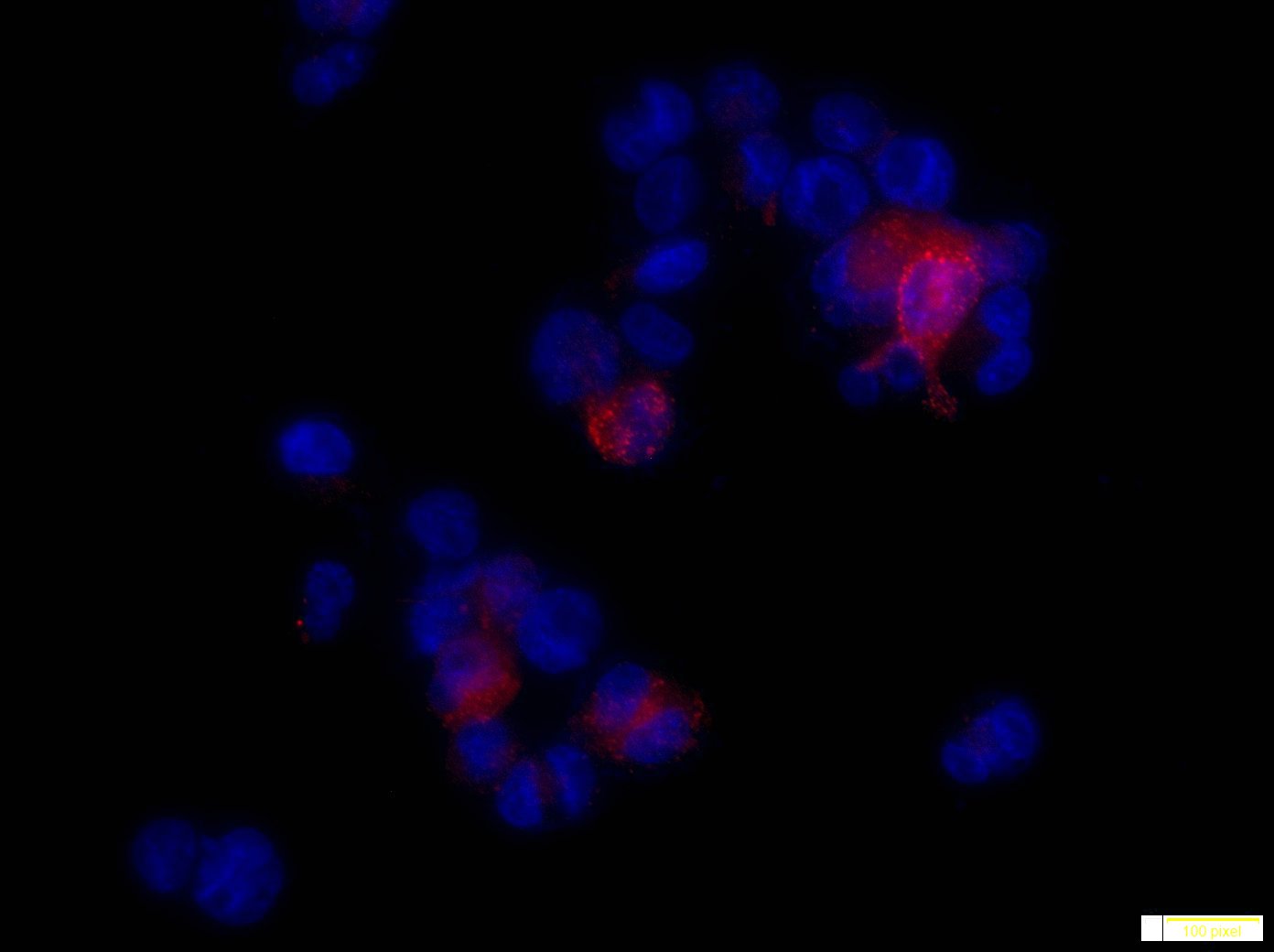

Supplement: Supplementary file 8 — Source data Fig. 2 [file 44318_2024_103_MOESM8_ESM.zip › Figure 2/2I/Macrophages.jpg]

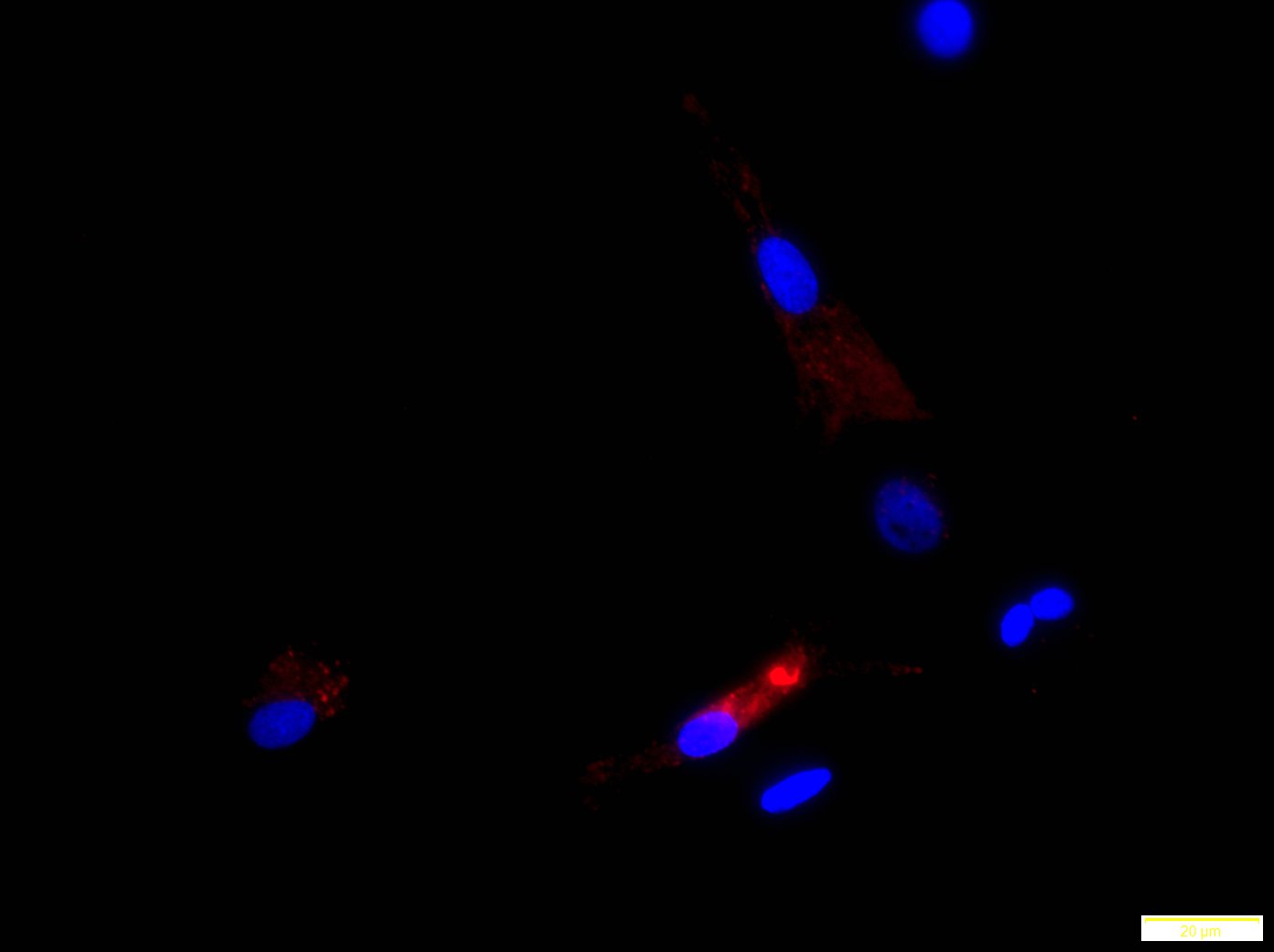

Supplement: Supplementary file 8 — Source data Fig. 2 [file 44318_2024_103_MOESM8_ESM.zip › Figure 2/2J/Macrophage-Fib co-culture.jpg]

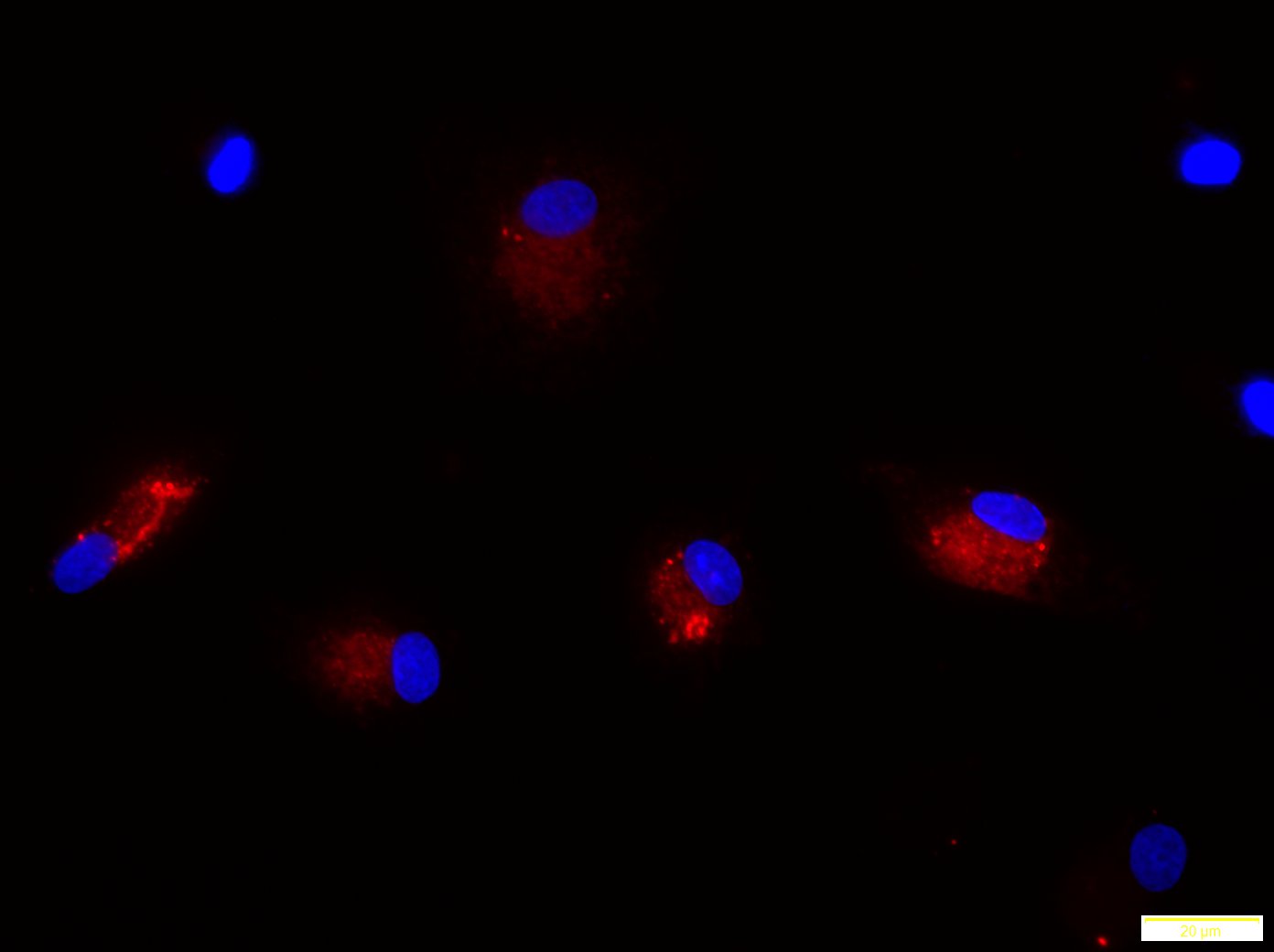

Supplement: Supplementary file 8 — Source data Fig. 2 [file 44318_2024_103_MOESM8_ESM.zip › Figure 2/2J/Macrophage-Ker co-culture.jpg]

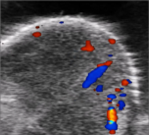

Supplement: Supplementary file 9 — Source data Fig. 3 [file 44318_2024_103_MOESM9_ESM.zip › Figure 3/3F/Representative image from the video_B16 control.tif]

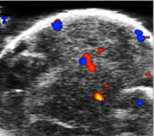

Supplement: Supplementary file 9 — Source data Fig. 3 [file 44318_2024_103_MOESM9_ESM.zip › Figure 3/3F/Representative image from the video_B16.tif]

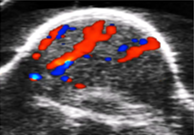

Supplement: Supplementary file 9 — Source data Fig. 3 [file 44318_2024_103_MOESM9_ESM.zip › Figure 3/3F/Representative image from the video_Fib.tif]

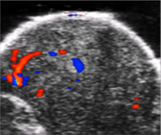

Supplement: Supplementary file 9 — Source data Fig. 3 [file 44318_2024_103_MOESM9_ESM.zip › Figure 3/3F/Representative image from the video_Ker.tif]

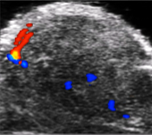

Supplement: Supplementary file 9 — Source data Fig. 3 [file 44318_2024_103_MOESM9_ESM.zip › Figure 3/3F/Representative image from the video_Naive.tif]

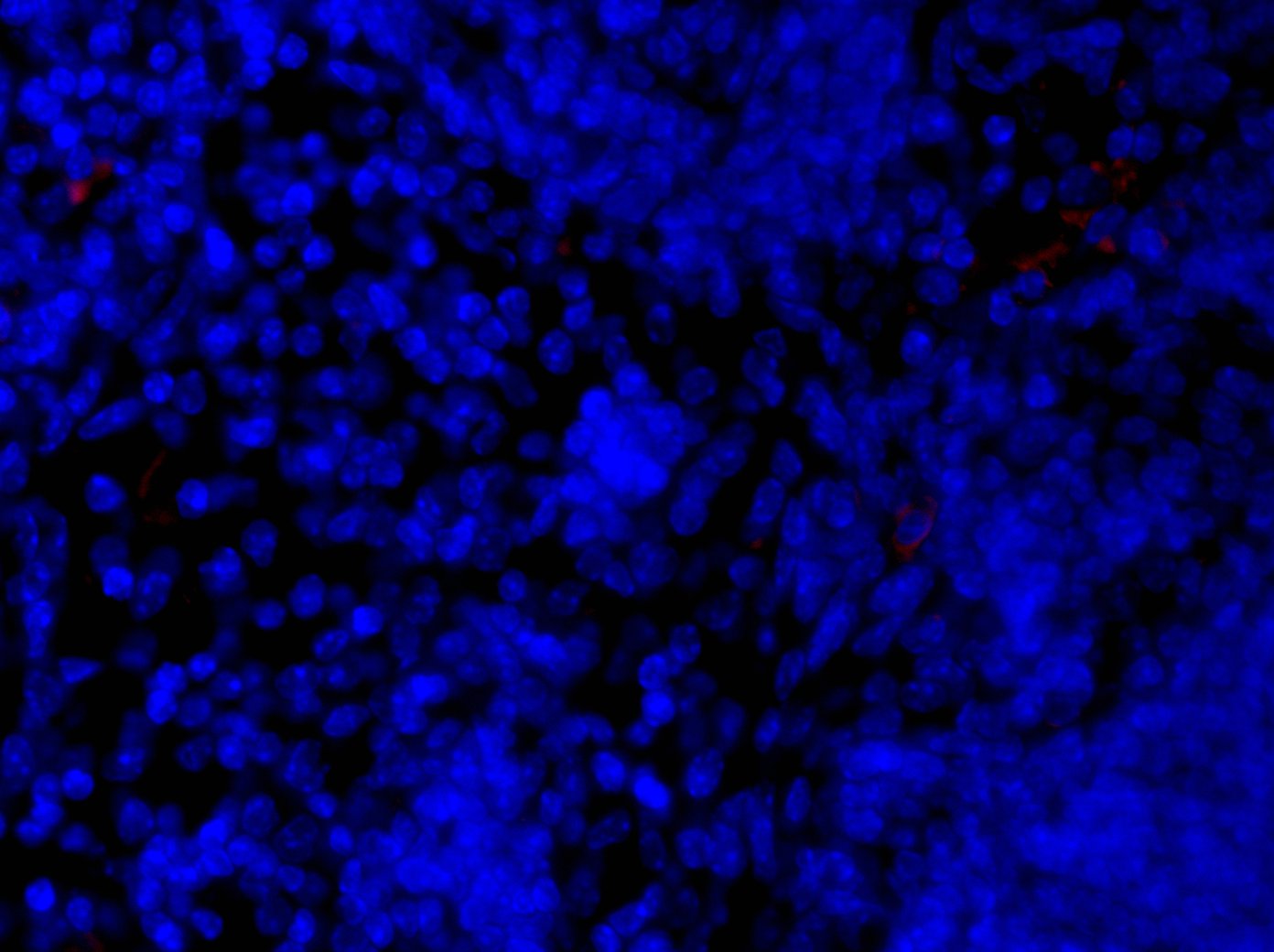

Supplement: Supplementary file 9 — Source data Fig. 3 [file 44318_2024_103_MOESM9_ESM.zip › Figure 3/3H/B16 control.jpg]

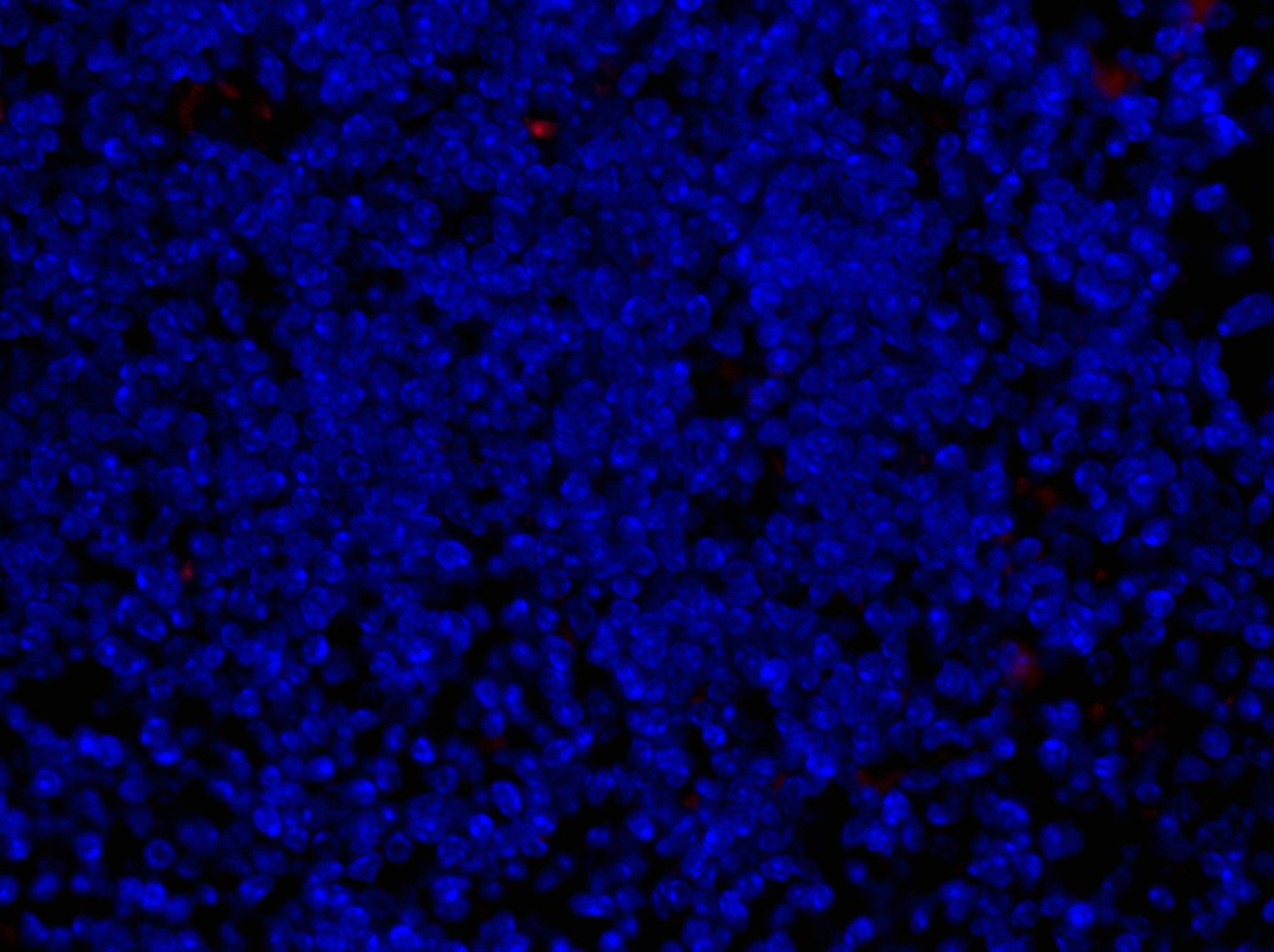

Supplement: Supplementary file 9 — Source data Fig. 3 [file 44318_2024_103_MOESM9_ESM.zip › Figure 3/3H/B16.jpg]

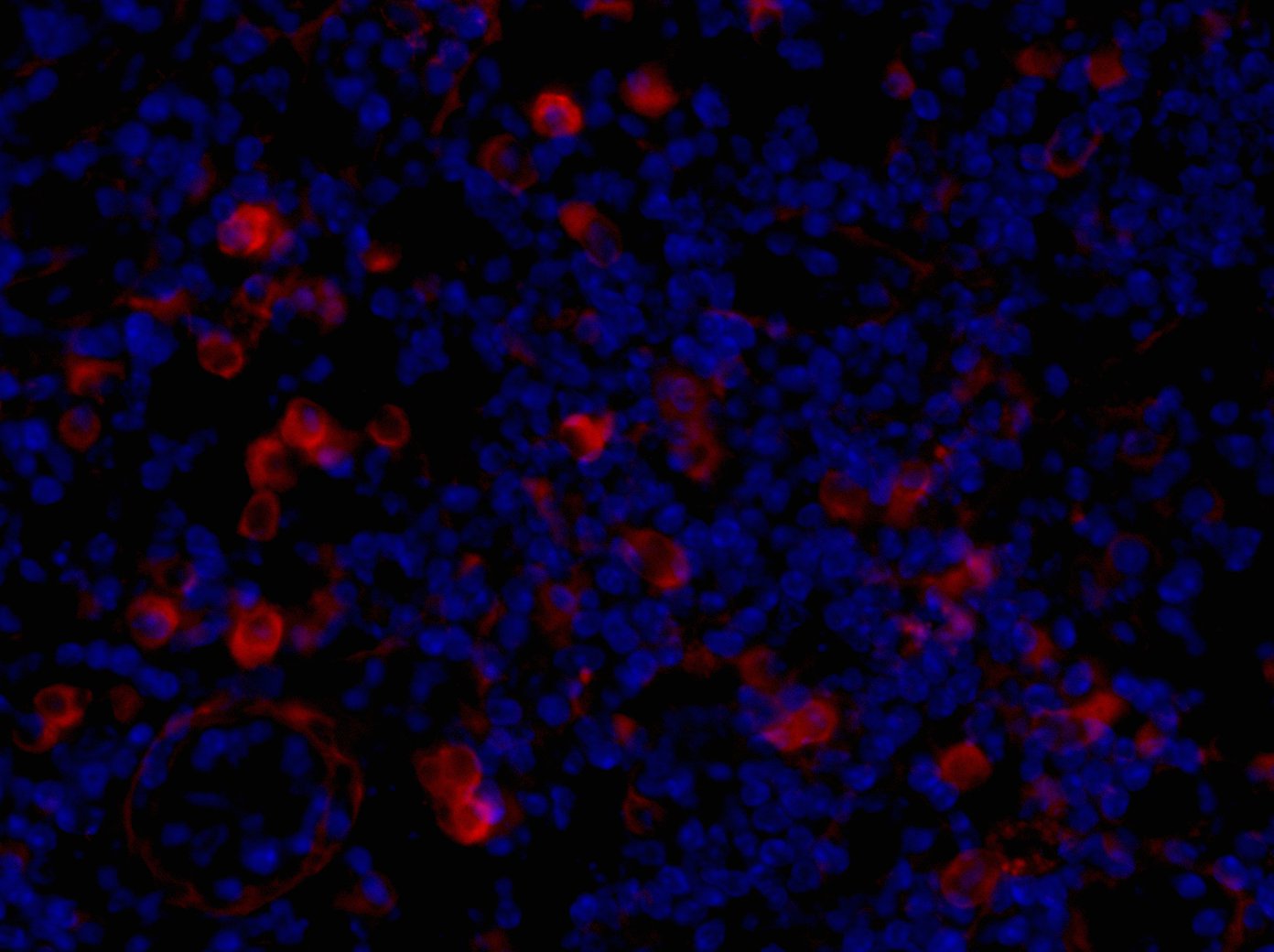

Supplement: Supplementary file 9 — Source data Fig. 3 [file 44318_2024_103_MOESM9_ESM.zip › Figure 3/3H/Fib.jpg]

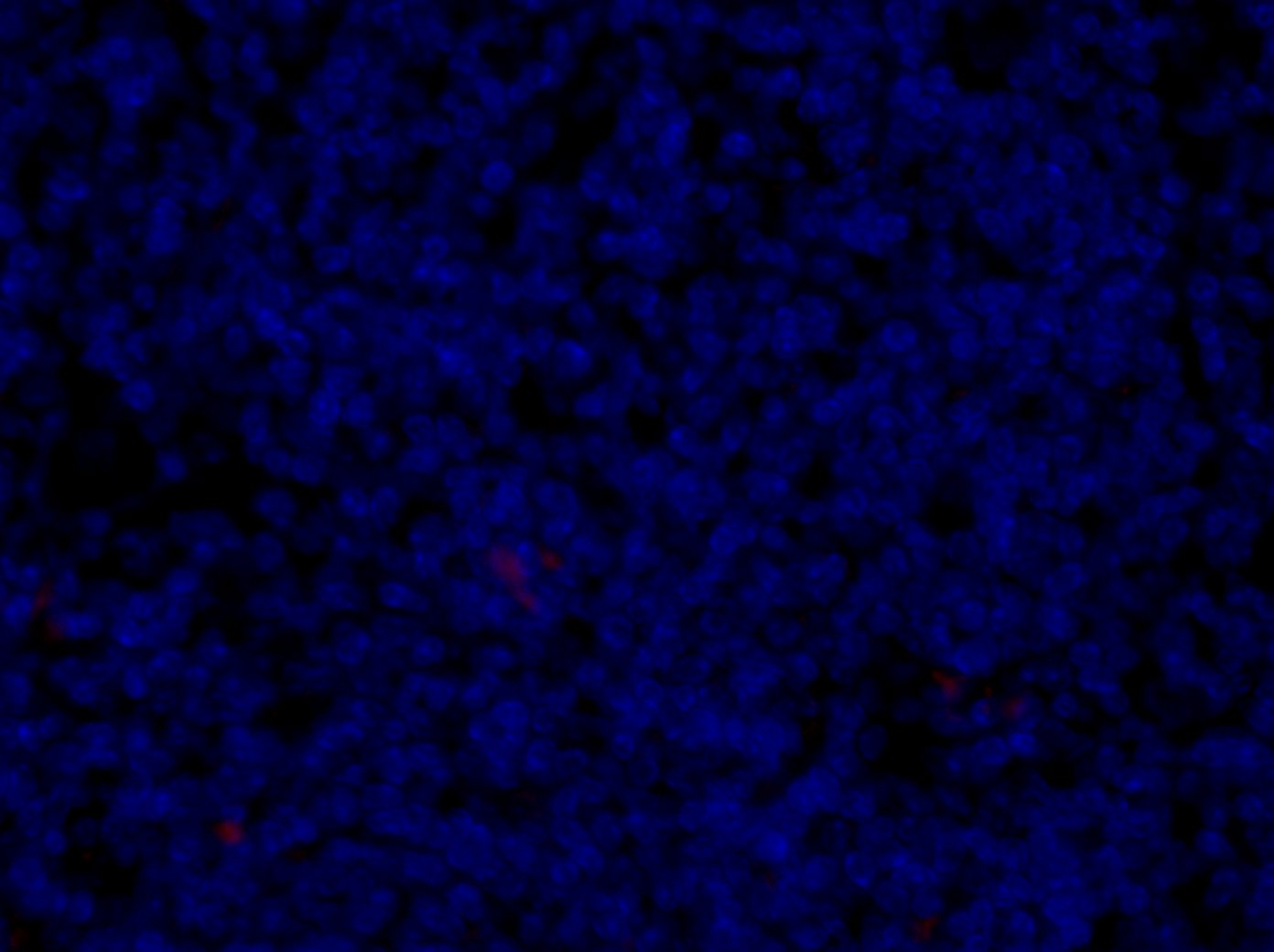

Supplement: Supplementary file 9 — Source data Fig. 3 [file 44318_2024_103_MOESM9_ESM.zip › Figure 3/3H/Ker.jpg]

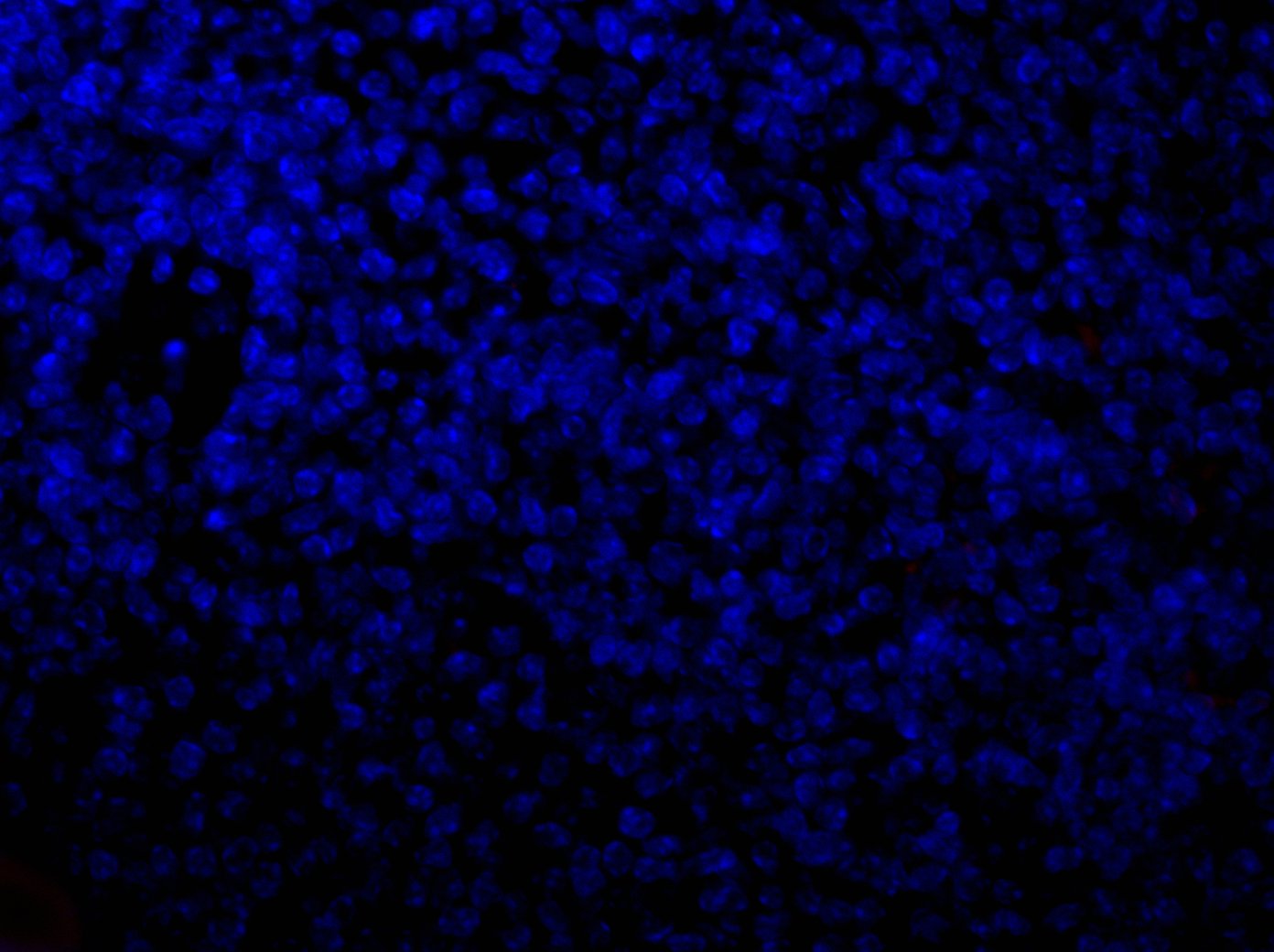

Supplement: Supplementary file 9 — Source data Fig. 3 [file 44318_2024_103_MOESM9_ESM.zip › Figure 3/3H/Naive.jpg]

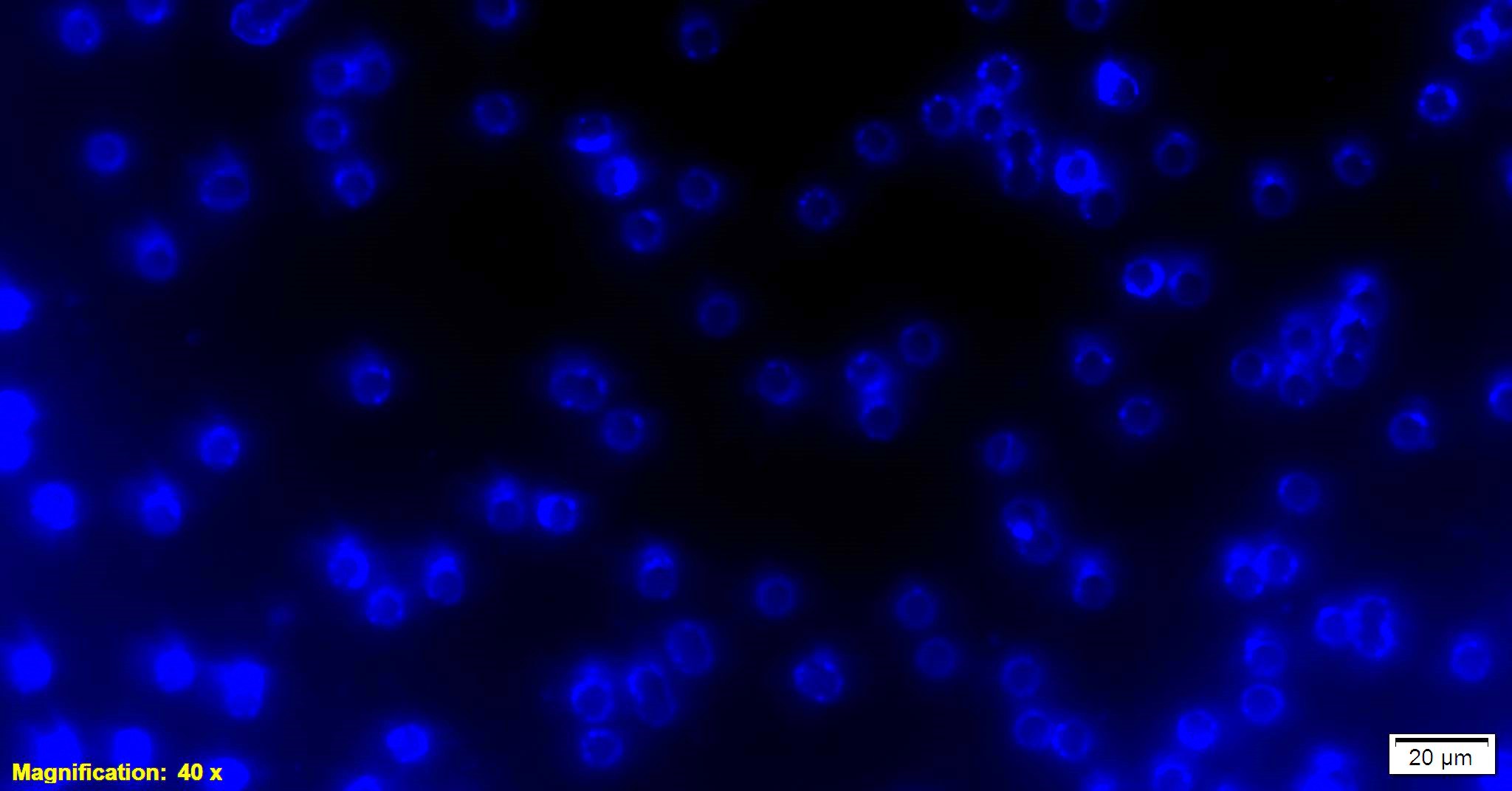

Supplement: Supplementary file 9 — Source data Fig. 3 [file 44318_2024_103_MOESM9_ESM.zip › Figure 3/3L/Fib.-MNT1.jpg]

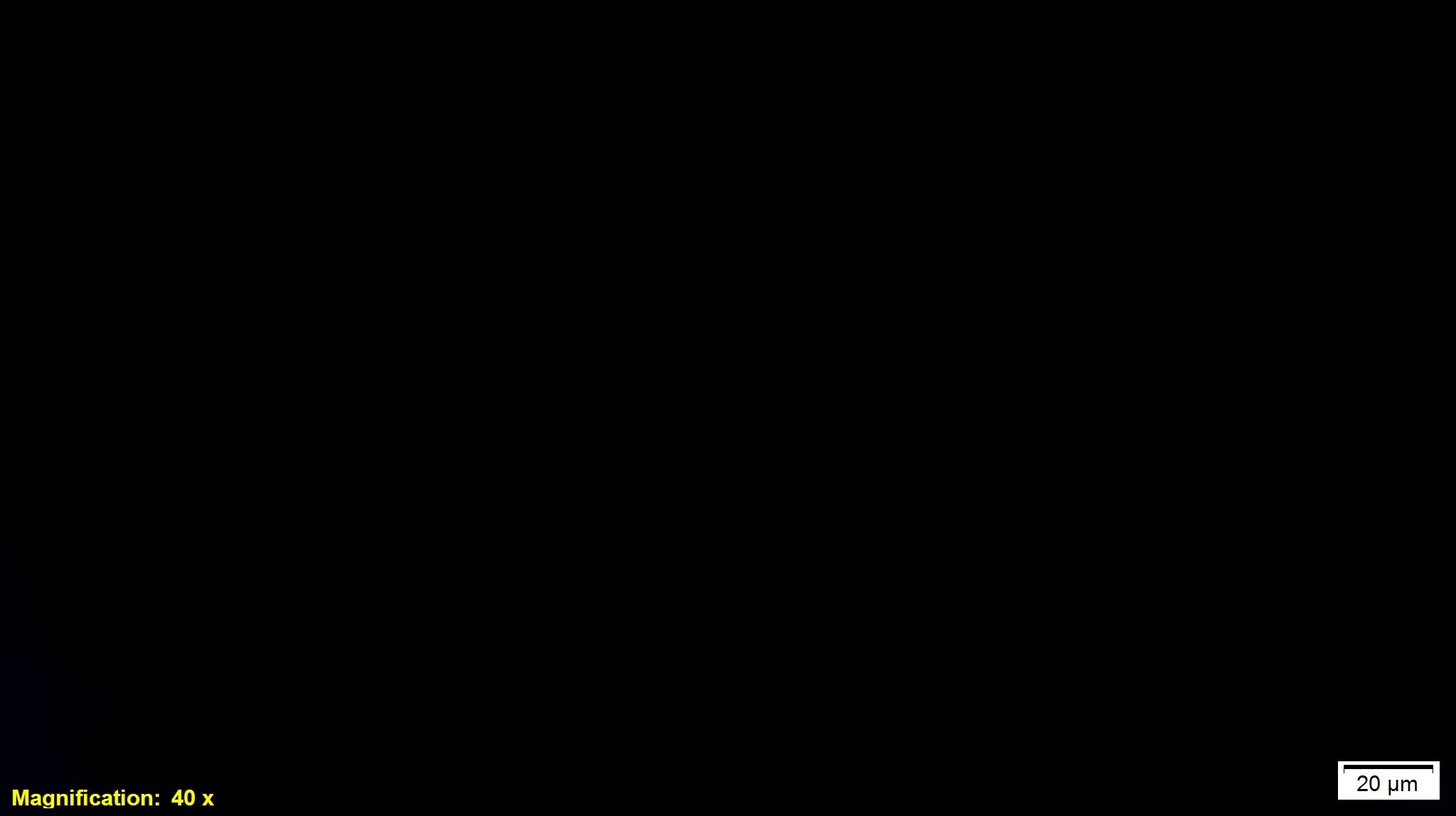

Supplement: Supplementary file 9 — Source data Fig. 3 [file 44318_2024_103_MOESM9_ESM.zip › Figure 3/3L/Ker.-MNT1.jpg]

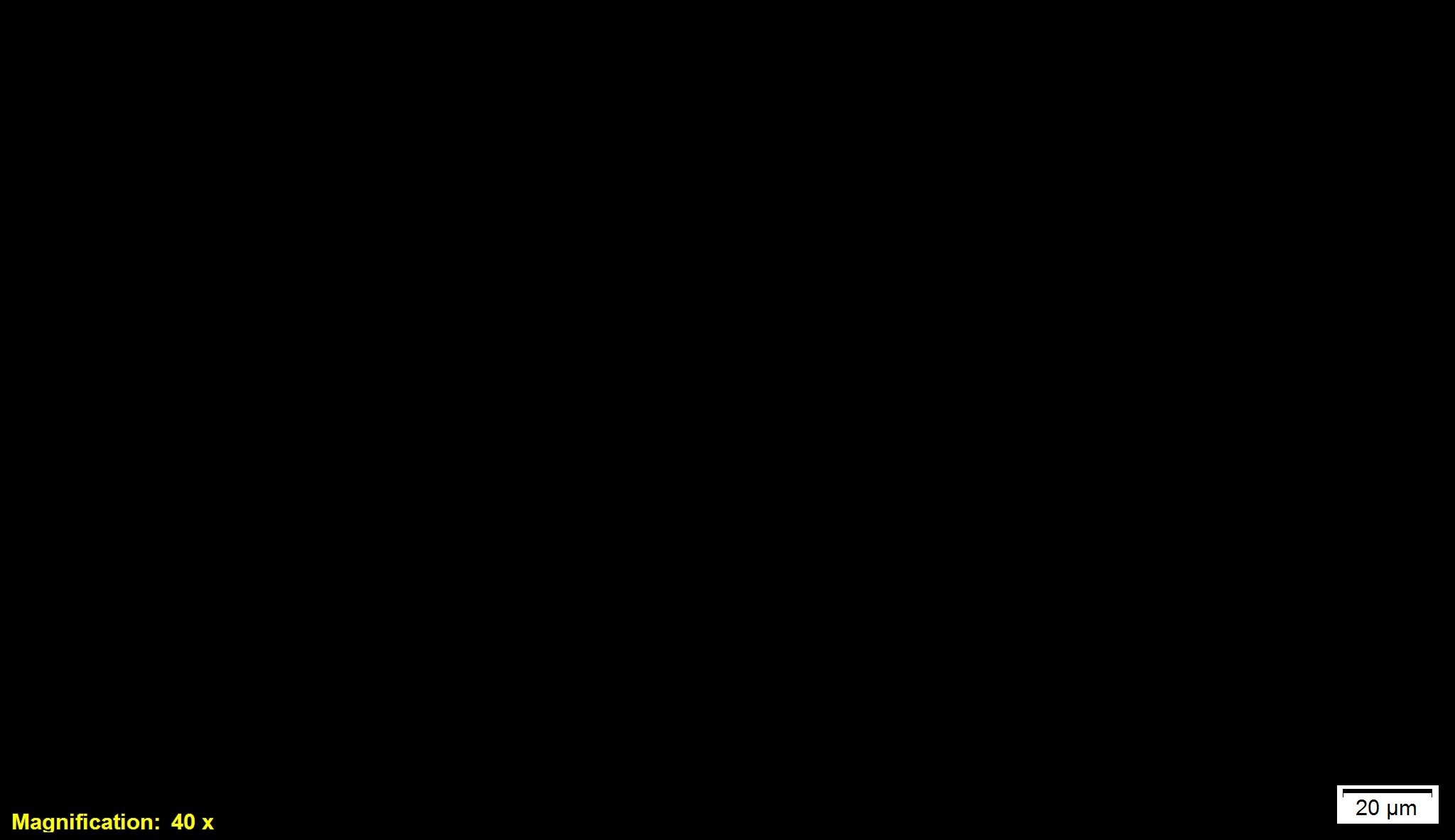

Supplement: Supplementary file 9 — Source data Fig. 3 [file 44318_2024_103_MOESM9_ESM.zip › Figure 3/3L/MNT1.jpg]

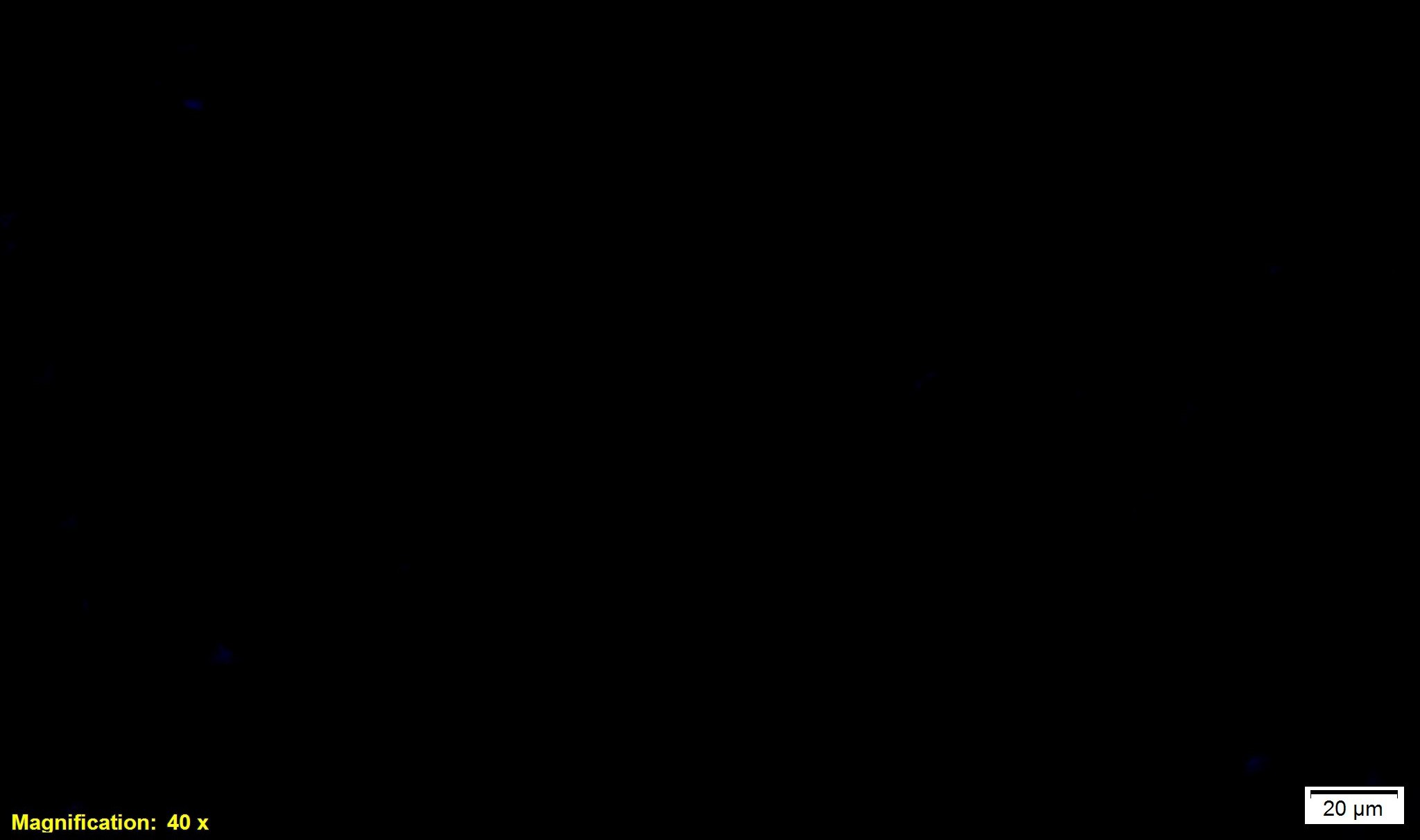

Supplement: Supplementary file 9 — Source data Fig. 3 [file 44318_2024_103_MOESM9_ESM.zip › Figure 3/3L/Naive.jpg]

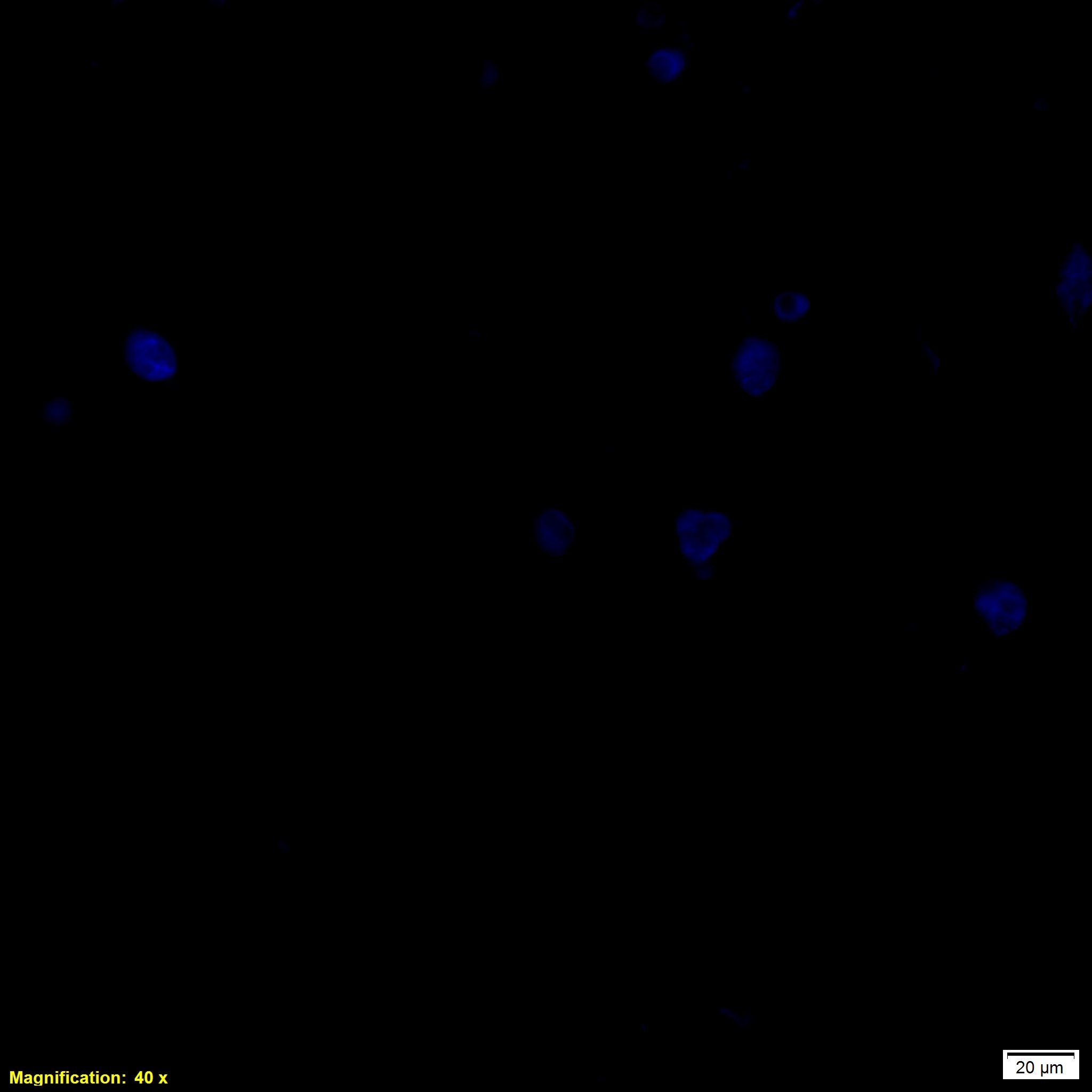

Supplement: Supplementary file 9 — Source data Fig. 3 [file 44318_2024_103_MOESM9_ESM.zip › Figure 3/3M/501 mel.jpg]

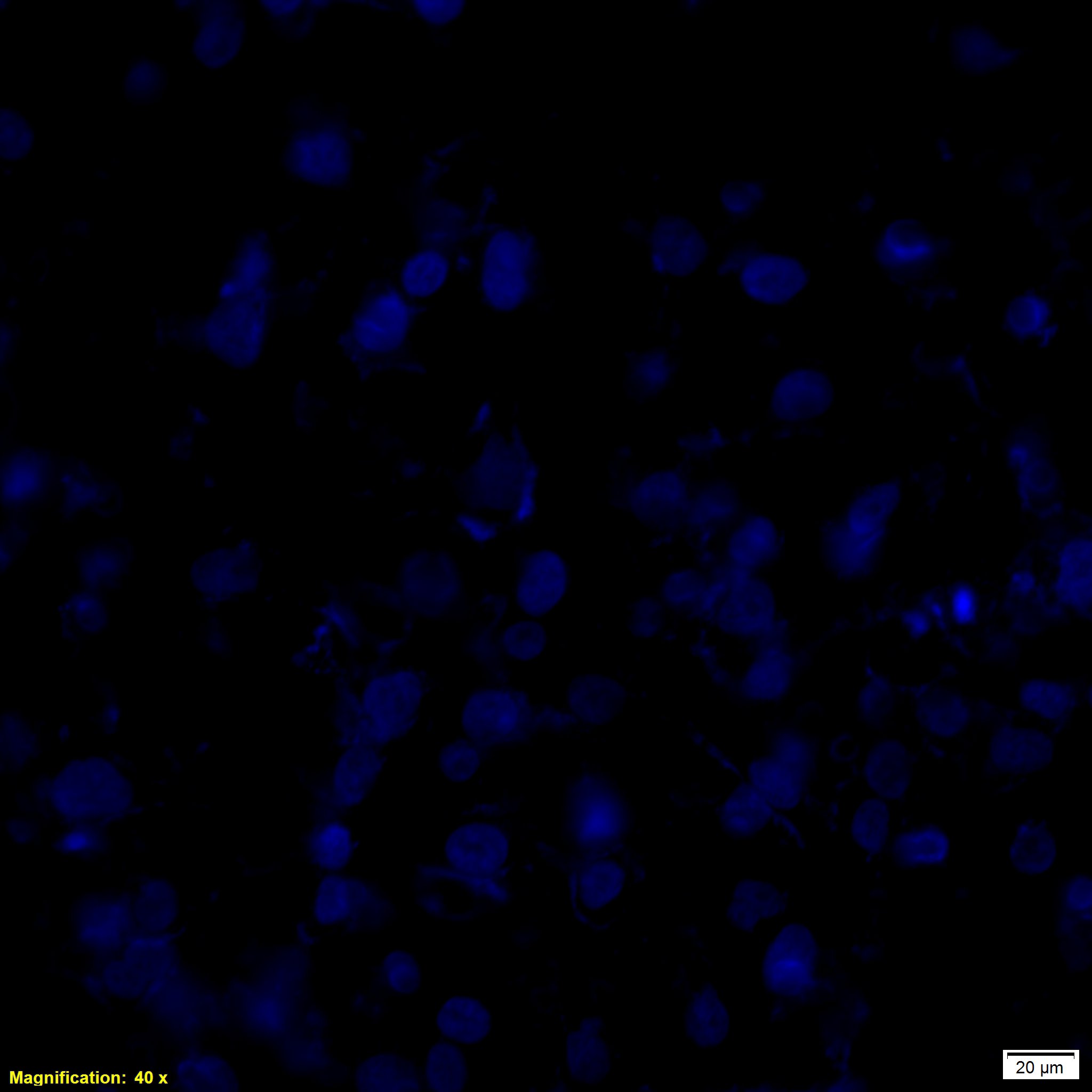

Supplement: Supplementary file 9 — Source data Fig. 3 [file 44318_2024_103_MOESM9_ESM.zip › Figure 3/3M/Fib.-501 mel.jpg]

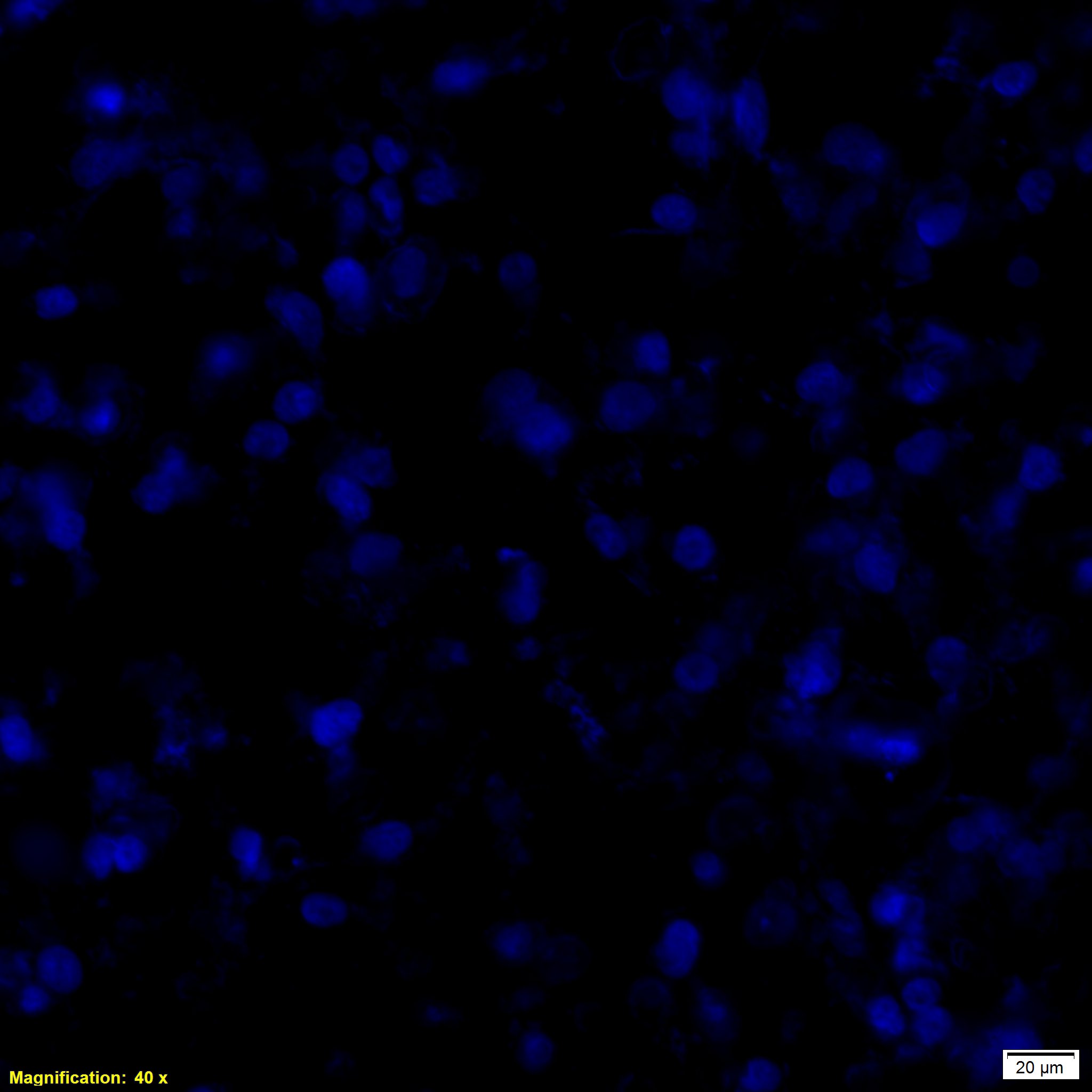

Supplement: Supplementary file 9 — Source data Fig. 3 [file 44318_2024_103_MOESM9_ESM.zip › Figure 3/3M/Fib.-WM3682.jpg]

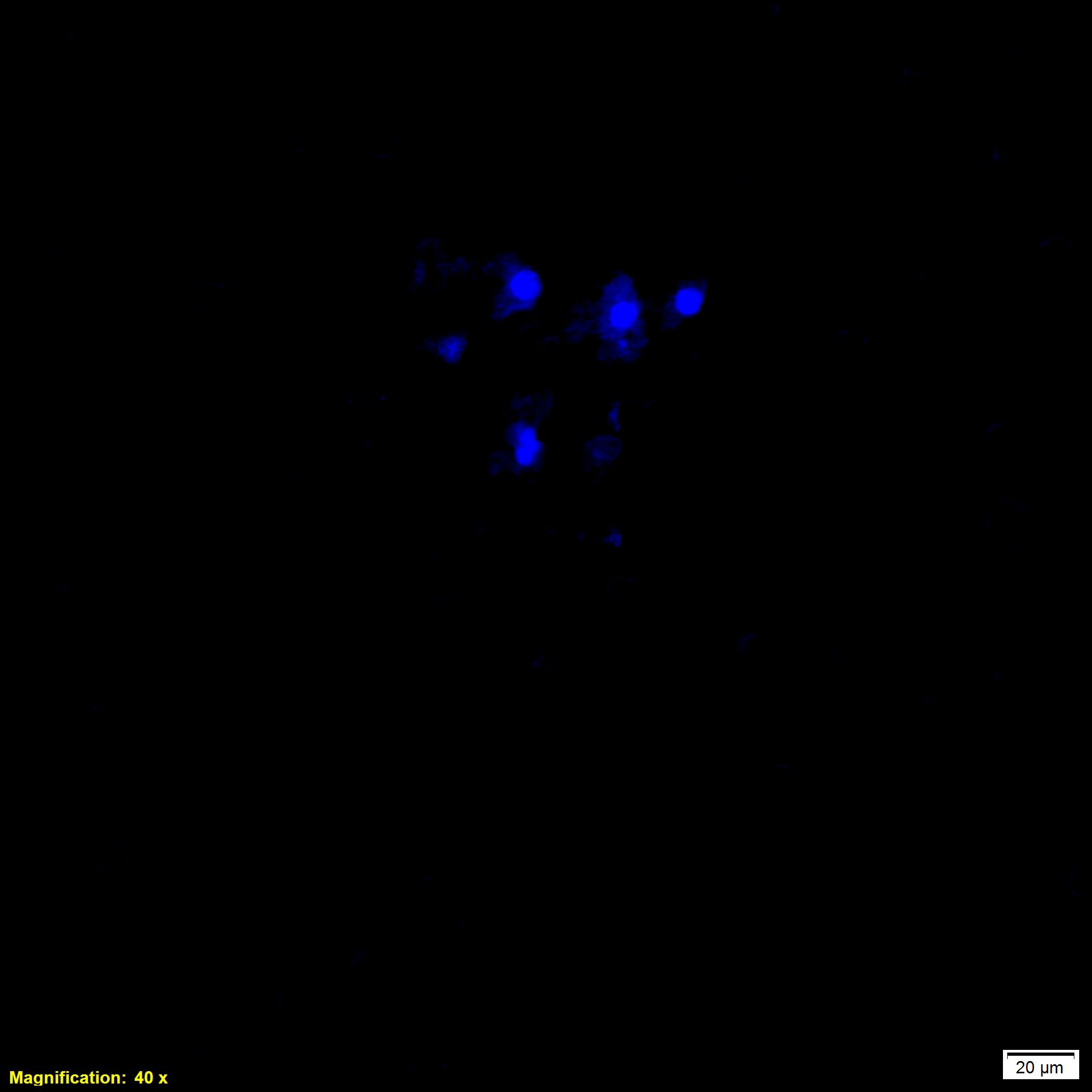

Supplement: Supplementary file 9 — Source data Fig. 3 [file 44318_2024_103_MOESM9_ESM.zip › Figure 3/3M/Naive.jpg]

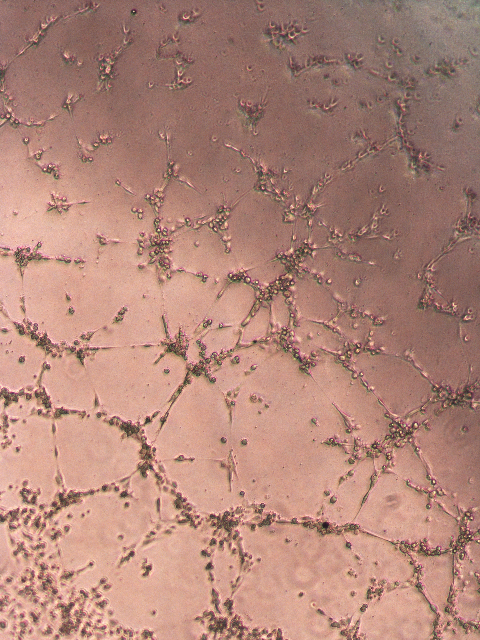

Supplement: Supplementary file 9 — Source data Fig. 3 [file 44318_2024_103_MOESM9_ESM.zip › Figure 3/3P/Fib.-MNT1.TIF]

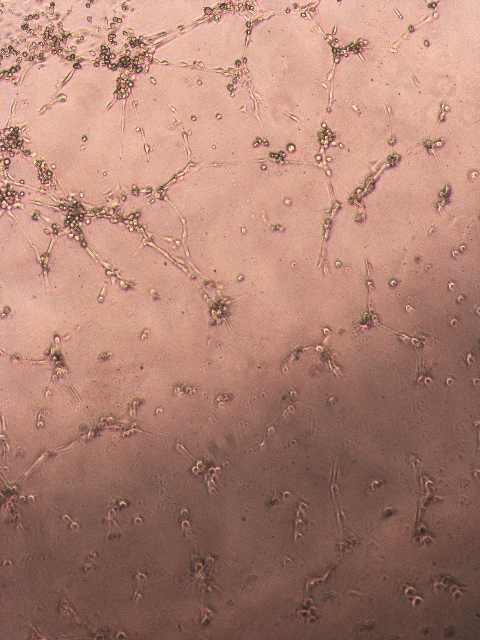

Supplement: Supplementary file 9 — Source data Fig. 3 [file 44318_2024_103_MOESM9_ESM.zip › Figure 3/3P/HDBEC control.TIF]

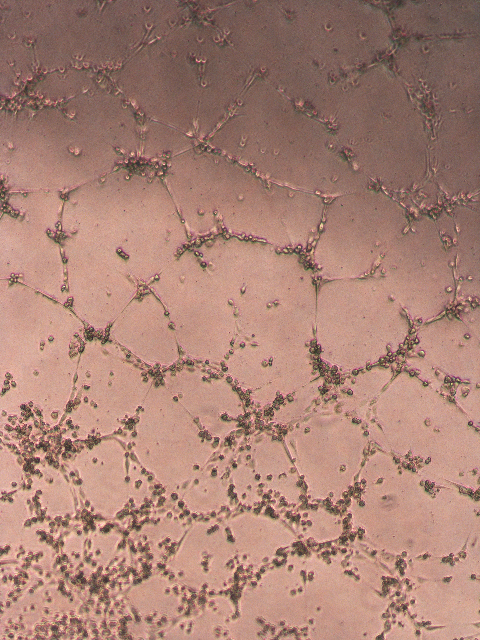

Supplement: Supplementary file 9 — Source data Fig. 3 [file 44318_2024_103_MOESM9_ESM.zip › Figure 3/3P/Ker.-MNT1.TIF]

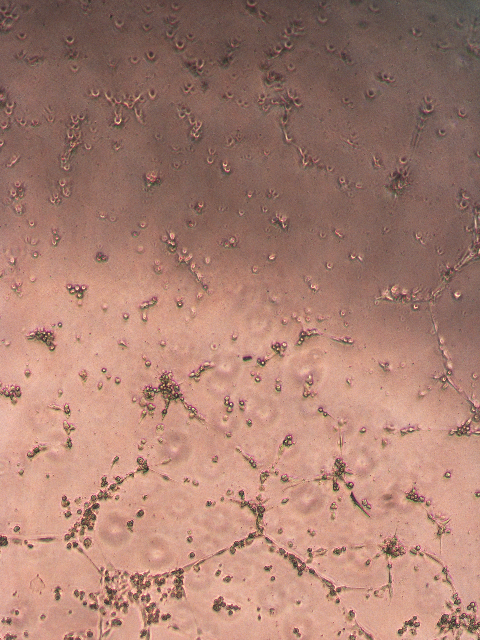

Supplement: Supplementary file 9 — Source data Fig. 3 [file 44318_2024_103_MOESM9_ESM.zip › Figure 3/3P/MNT1.TIF]

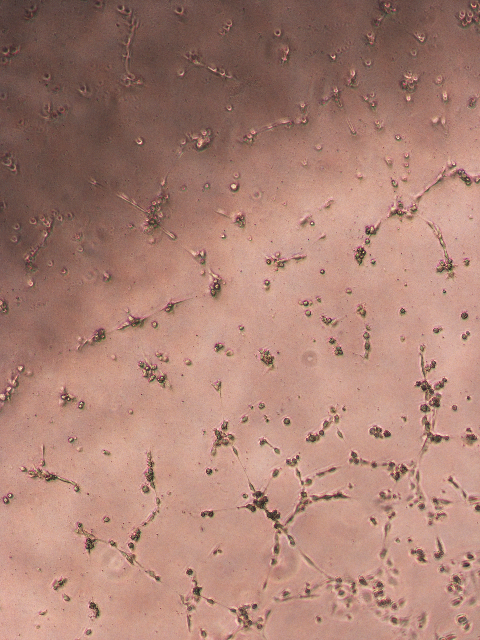

Supplement: Supplementary file 9 — Source data Fig. 3 [file 44318_2024_103_MOESM9_ESM.zip › Figure 3/3P/Naive.TIF]

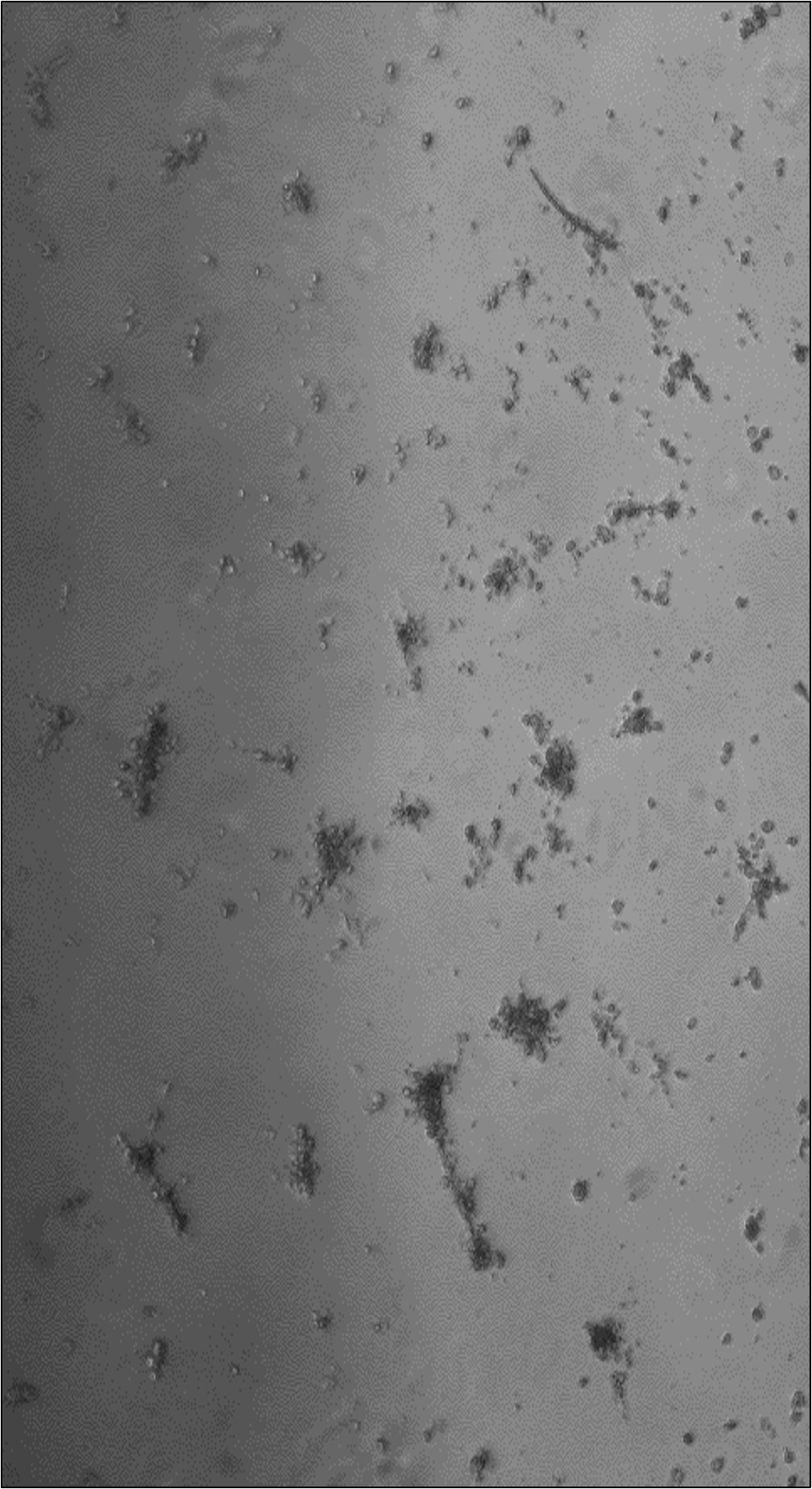

Supplement: Supplementary file 9 — Source data Fig. 3 [file 44318_2024_103_MOESM9_ESM.zip › Figure 3/3Q/501 mel.tif]

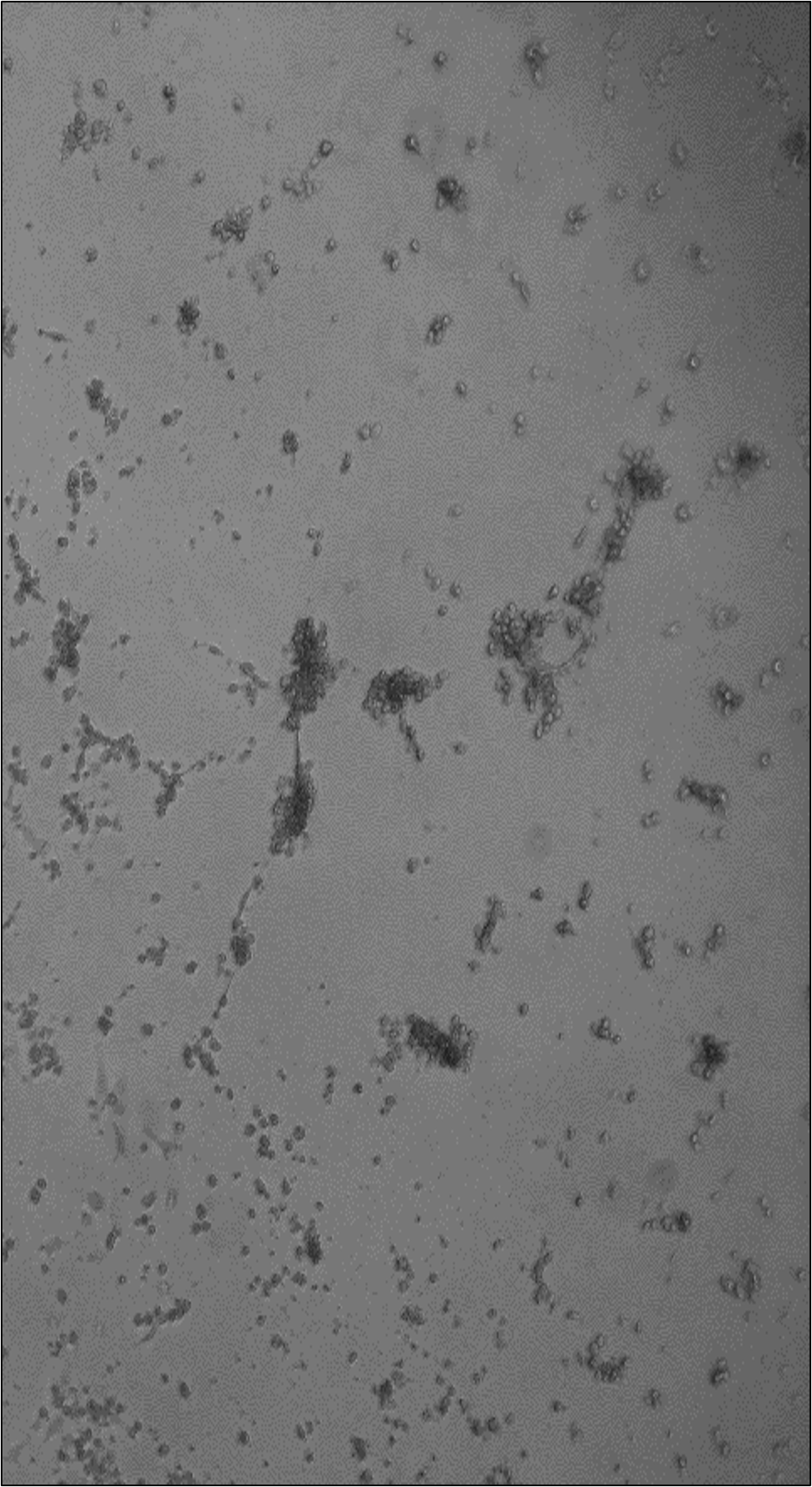

Supplement: Supplementary file 9 — Source data Fig. 3 [file 44318_2024_103_MOESM9_ESM.zip › Figure 3/3Q/Fib.-501 mel.tif]

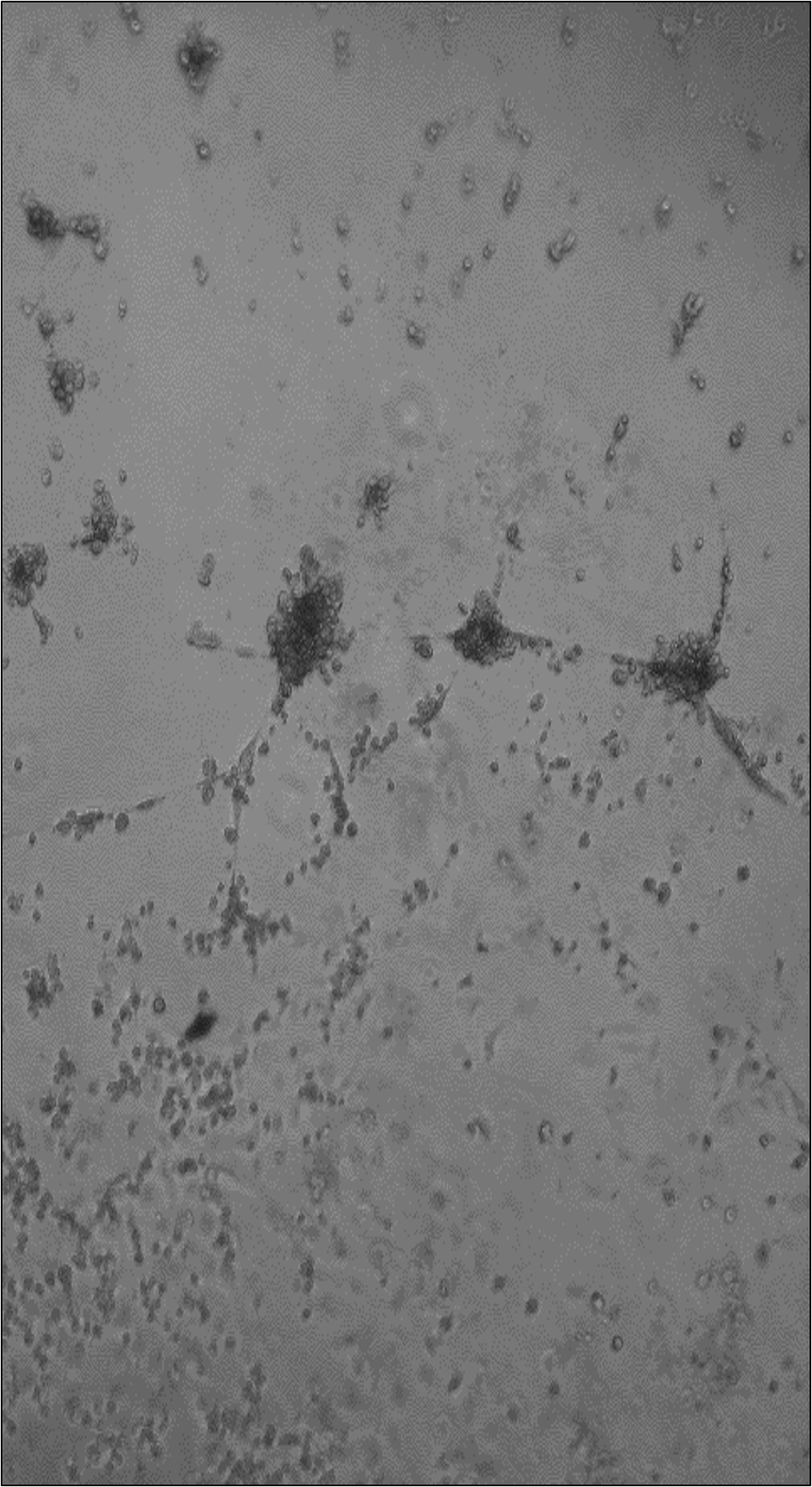

Supplement: Supplementary file 9 — Source data Fig. 3 [file 44318_2024_103_MOESM9_ESM.zip › Figure 3/3Q/Fib.-WM3682.tif]

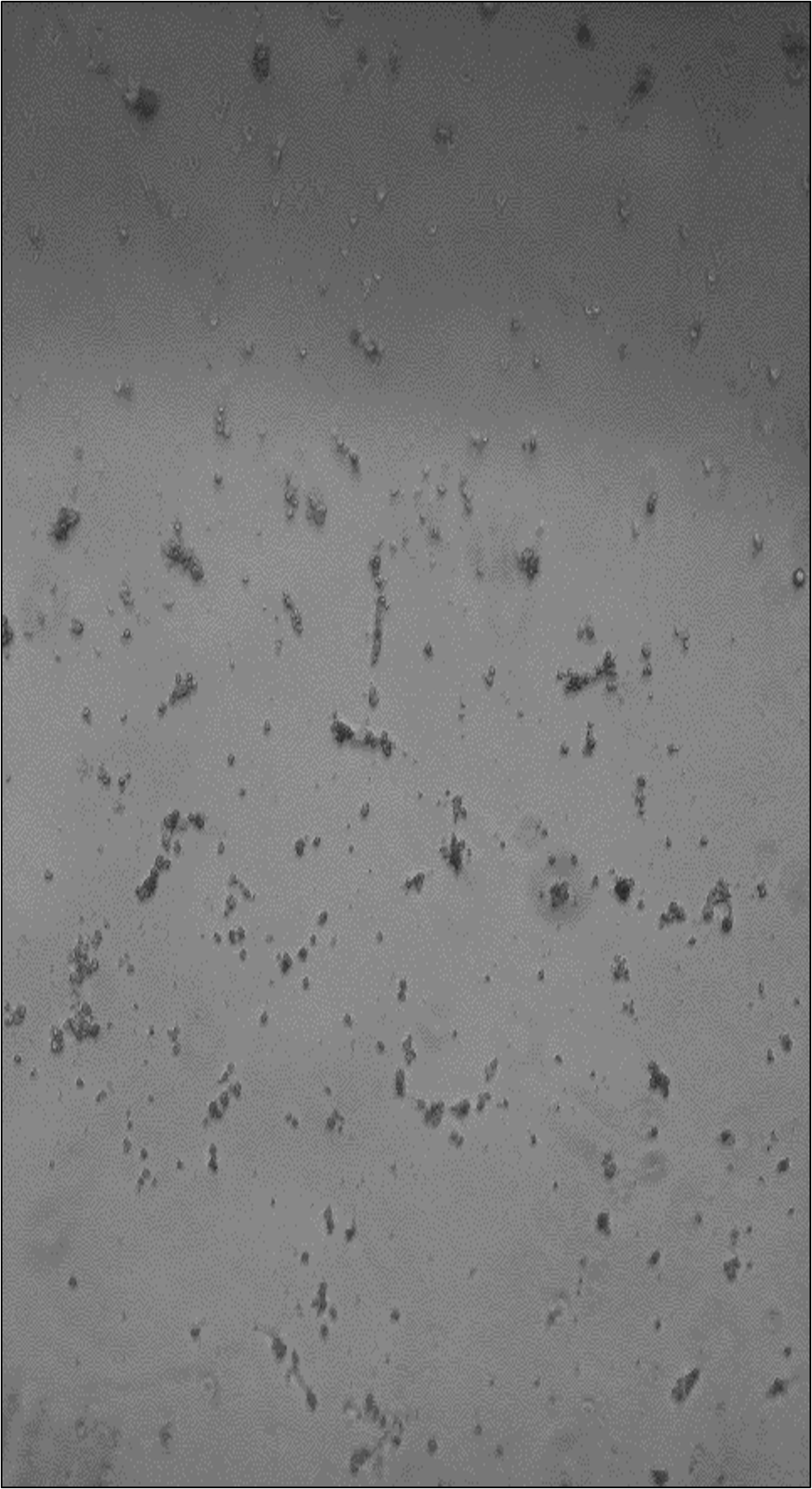

Supplement: Supplementary file 9 — Source data Fig. 3 [file 44318_2024_103_MOESM9_ESM.zip › Figure 3/3Q/HDBEC control.tif]

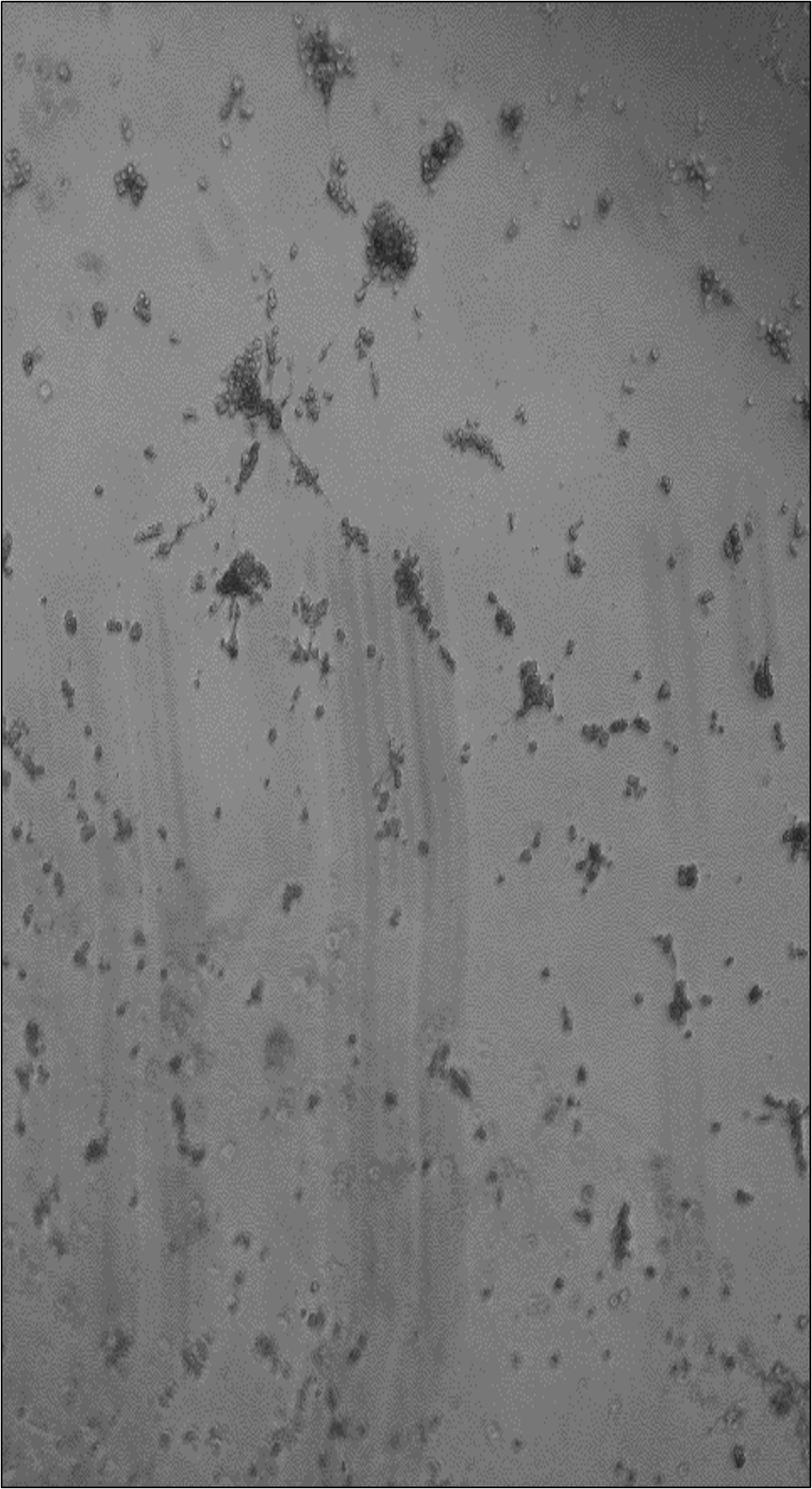

Supplement: Supplementary file 9 — Source data Fig. 3 [file 44318_2024_103_MOESM9_ESM.zip › Figure 3/3Q/WM3682.tif]

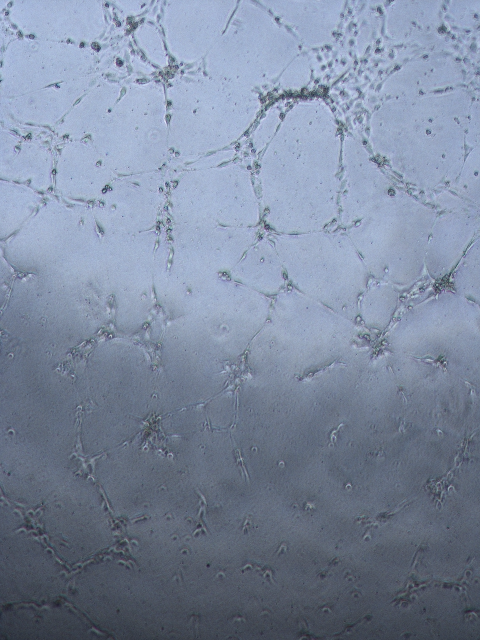

Supplement: Supplementary file 11 — Source data Fig. 5 [file 44318_2024_103_MOESM11_ESM.zip › Figure 5/5F/HDBEC control.TIF]

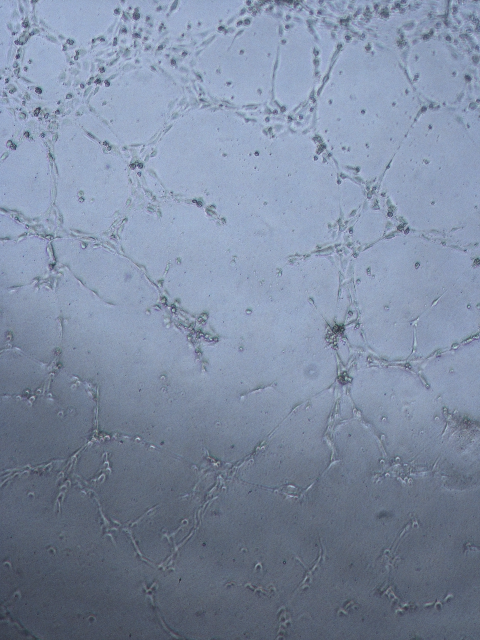

Supplement: Supplementary file 11 — Source data Fig. 5 [file 44318_2024_103_MOESM11_ESM.zip › Figure 5/5F/Naive.TIF]

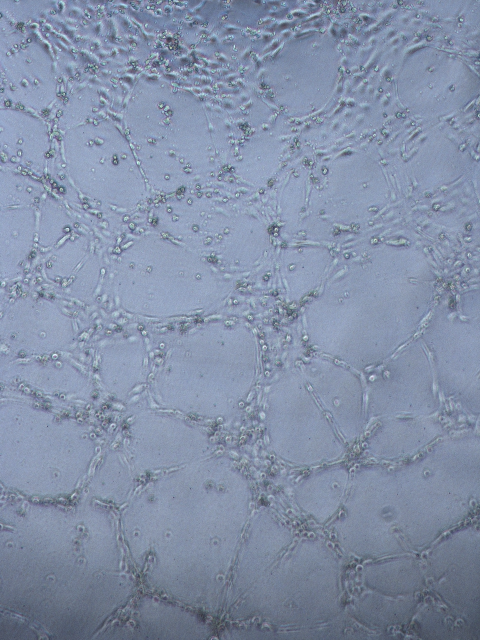

Supplement: Supplementary file 11 — Source data Fig. 5 [file 44318_2024_103_MOESM11_ESM.zip › Figure 5/5F/Non-trypsinized.TIF]

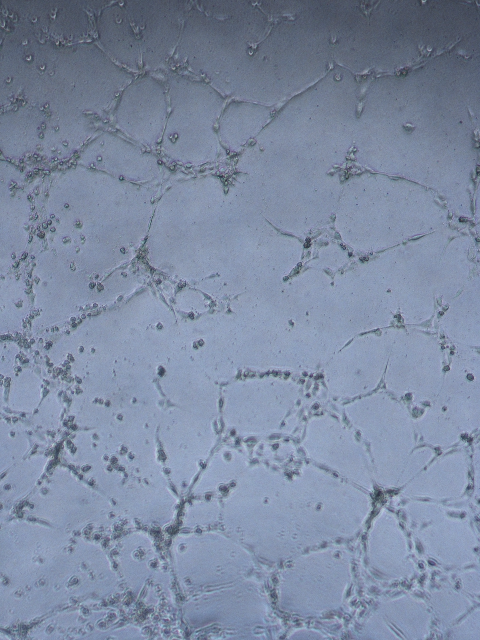

Supplement: Supplementary file 11 — Source data Fig. 5 [file 44318_2024_103_MOESM11_ESM.zip › Figure 5/5F/Trypsinized.TIF]

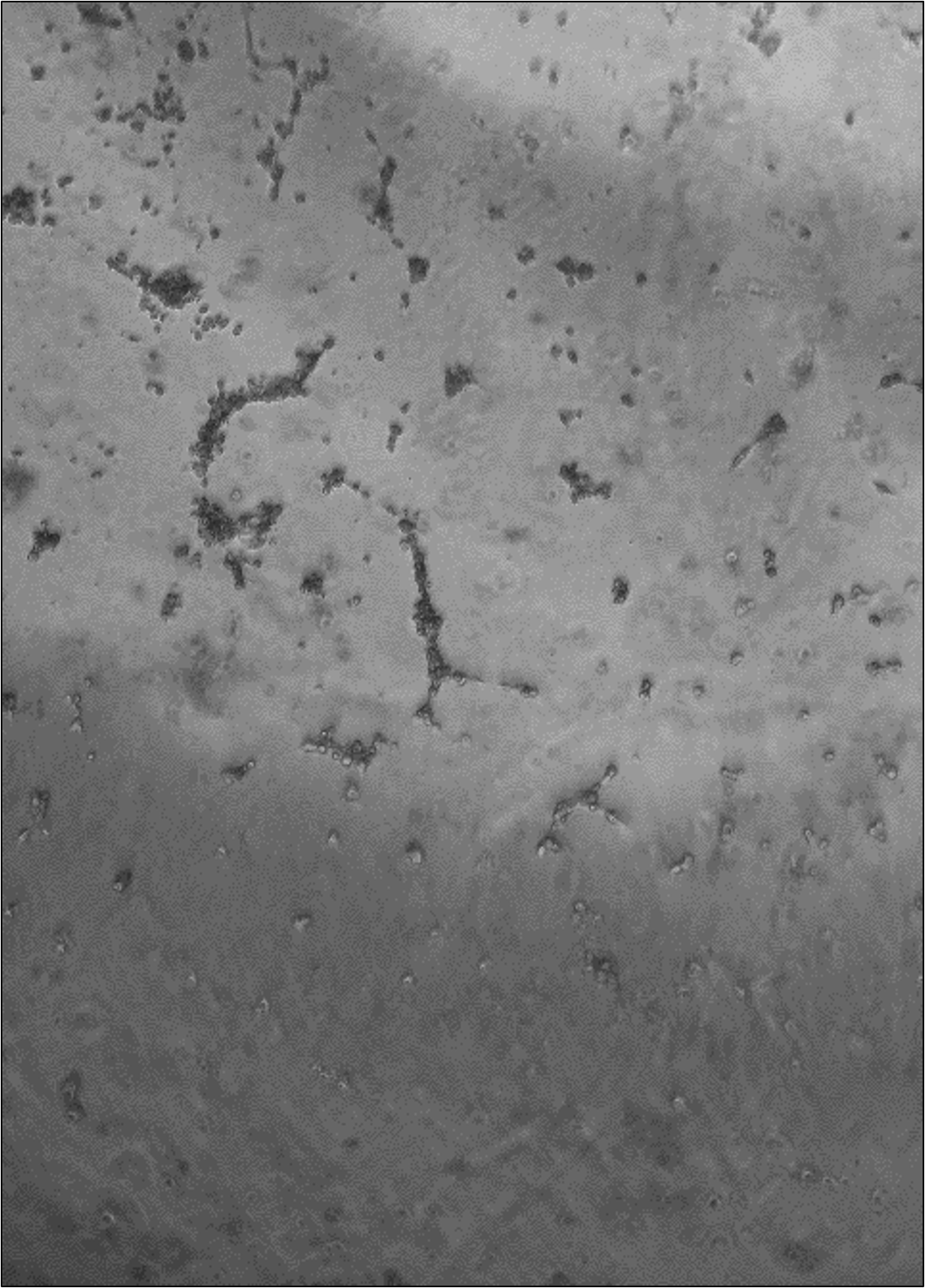

Supplement: Supplementary file 11 — Source data Fig. 5 [file 44318_2024_103_MOESM11_ESM.zip › Figure 5/5G/- AKTi.tif]

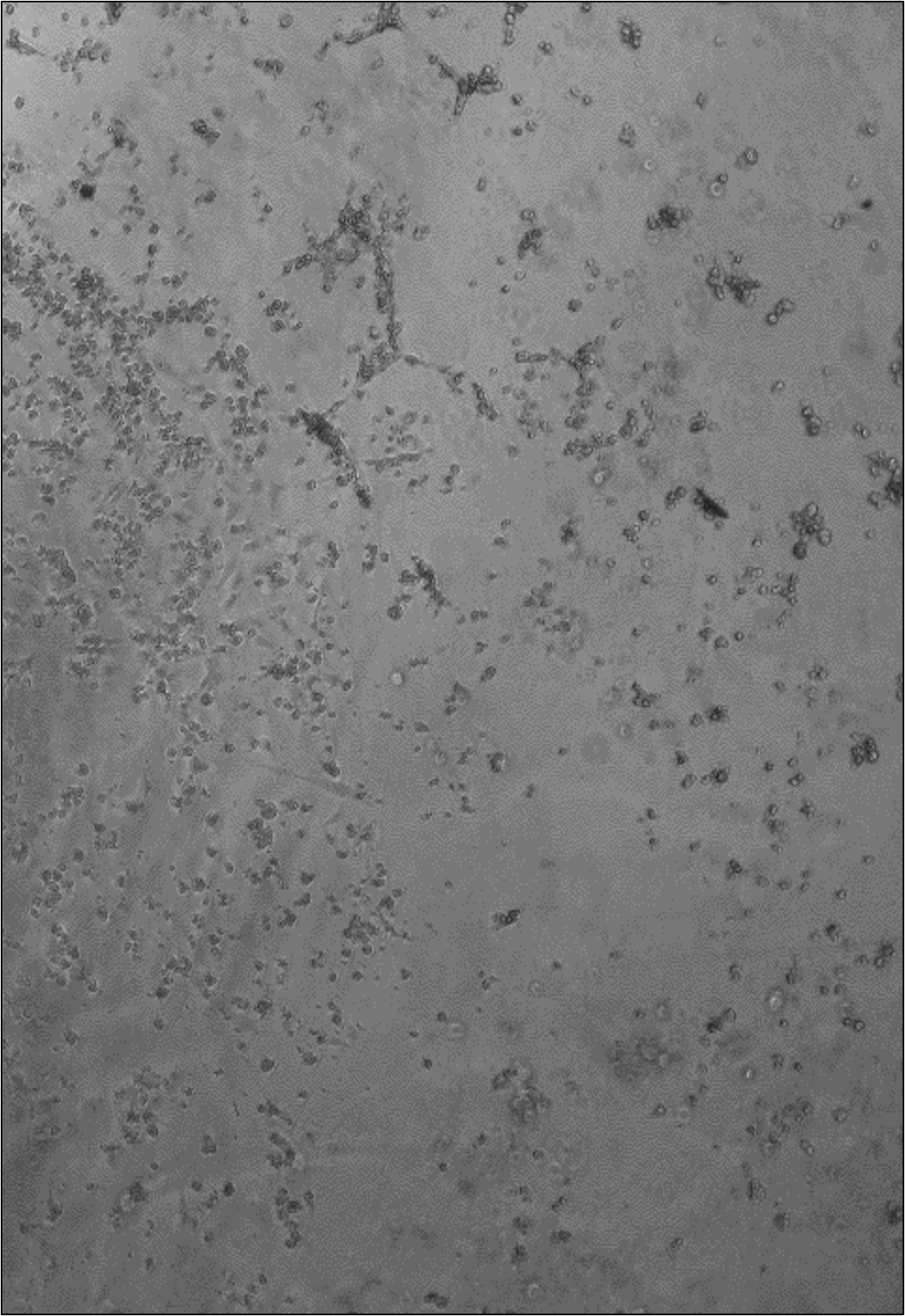

Supplement: Supplementary file 11 — Source data Fig. 5 [file 44318_2024_103_MOESM11_ESM.zip › Figure 5/5G/+ AKTi.tif]

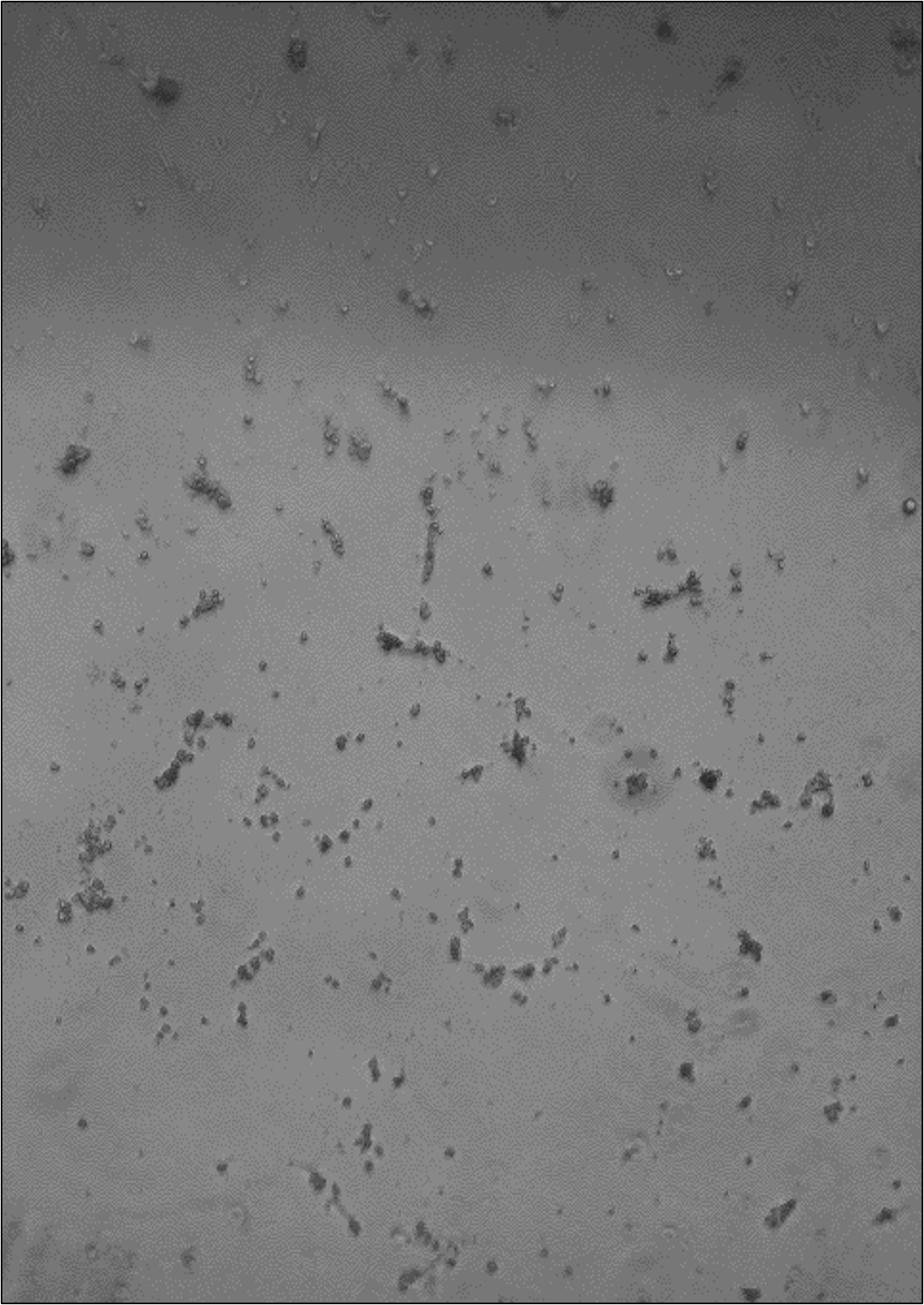

Supplement: Supplementary file 11 — Source data Fig. 5 [file 44318_2024_103_MOESM11_ESM.zip › Figure 5/5G/HDBEC control.tif]

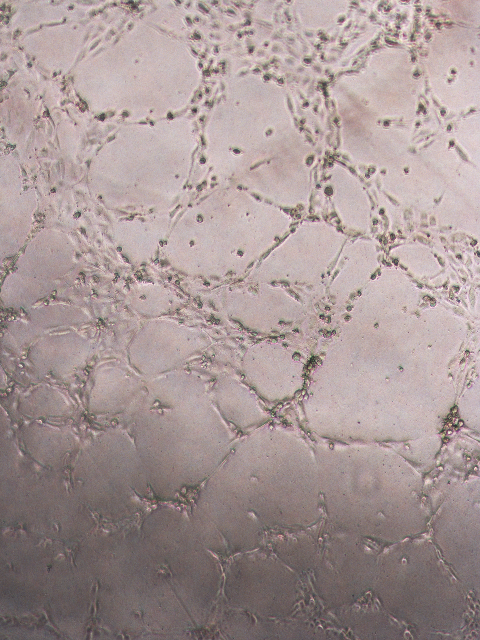

Supplement: Supplementary file 11 — Source data Fig. 5 [file 44318_2024_103_MOESM11_ESM.zip › Figure 5/5H/- Rapamycin.TIF]

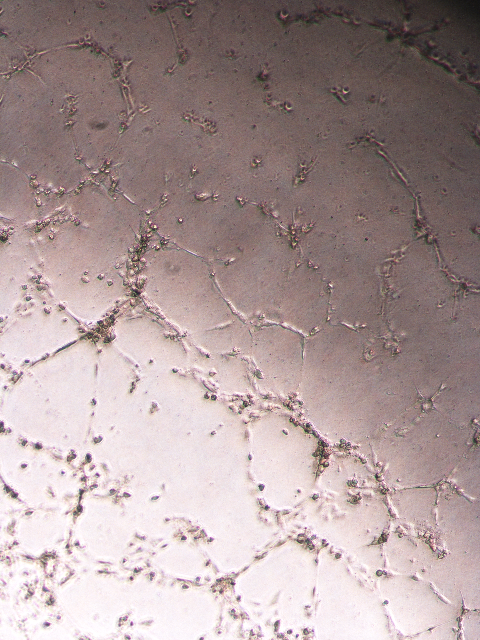

Supplement: Supplementary file 11 — Source data Fig. 5 [file 44318_2024_103_MOESM11_ESM.zip › Figure 5/5H/+ Rapamycin.TIF]

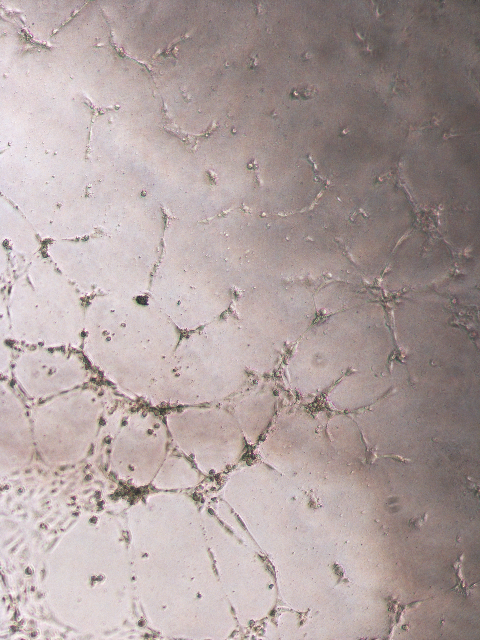

Supplement: Supplementary file 11 — Source data Fig. 5 [file 44318_2024_103_MOESM11_ESM.zip › Figure 5/5H/HDBEC control.TIF]

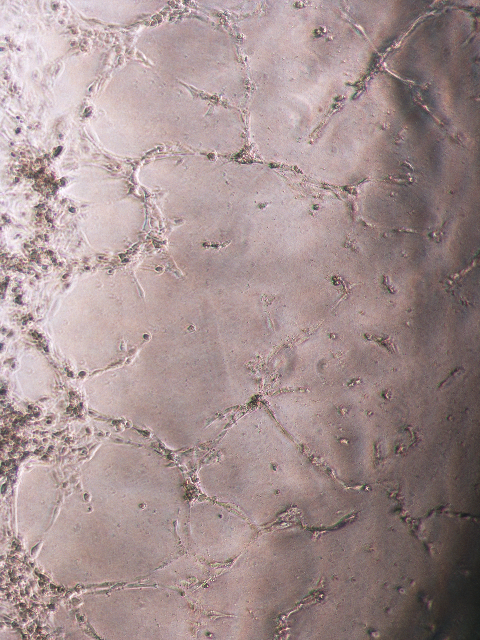

Supplement: Supplementary file 11 — Source data Fig. 5 [file 44318_2024_103_MOESM11_ESM.zip › Figure 5/5H/Naive.TIF]

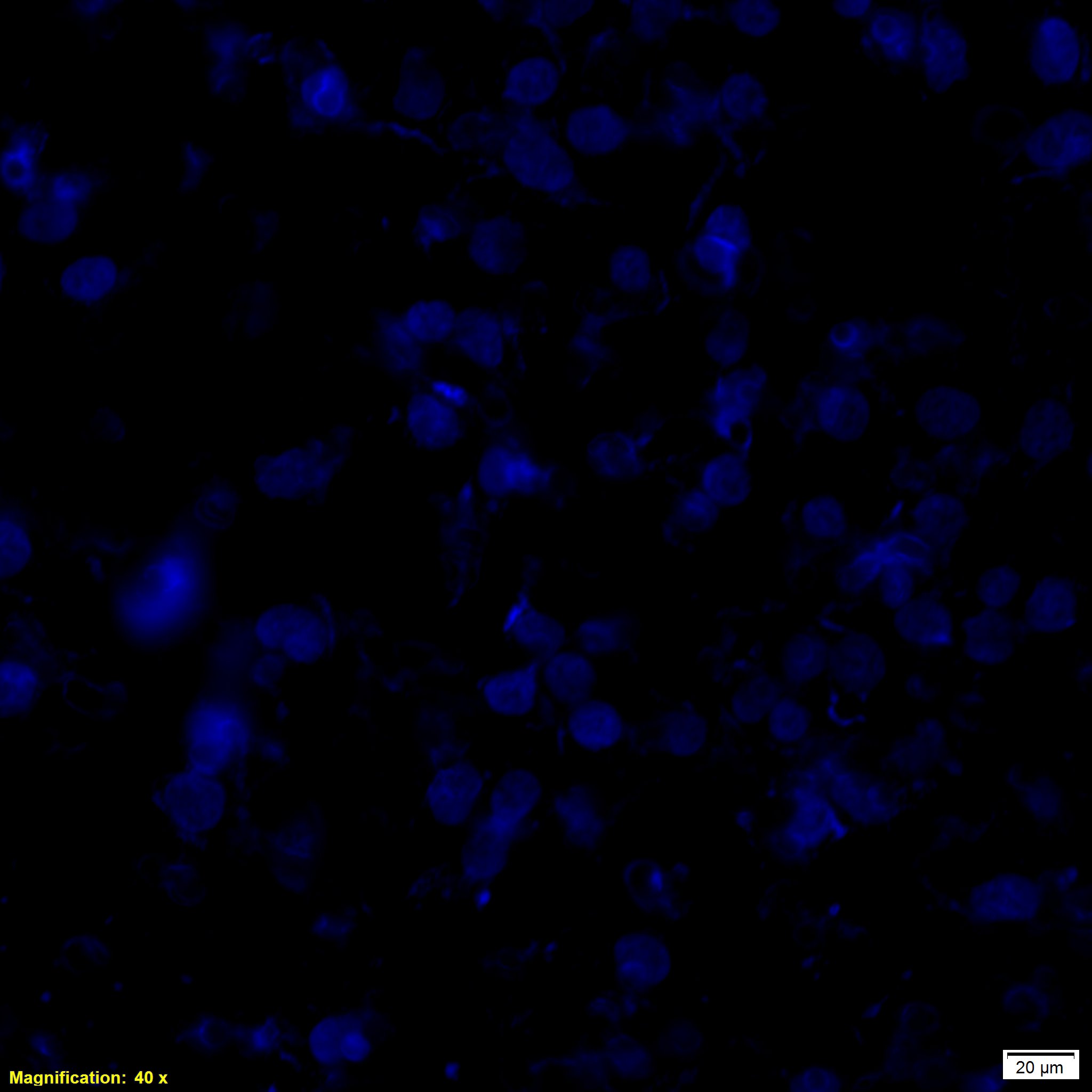

Supplement: Supplementary file 11 — Source data Fig. 5 [file 44318_2024_103_MOESM11_ESM.zip › Figure 5/5J/- AKTi.jpg]

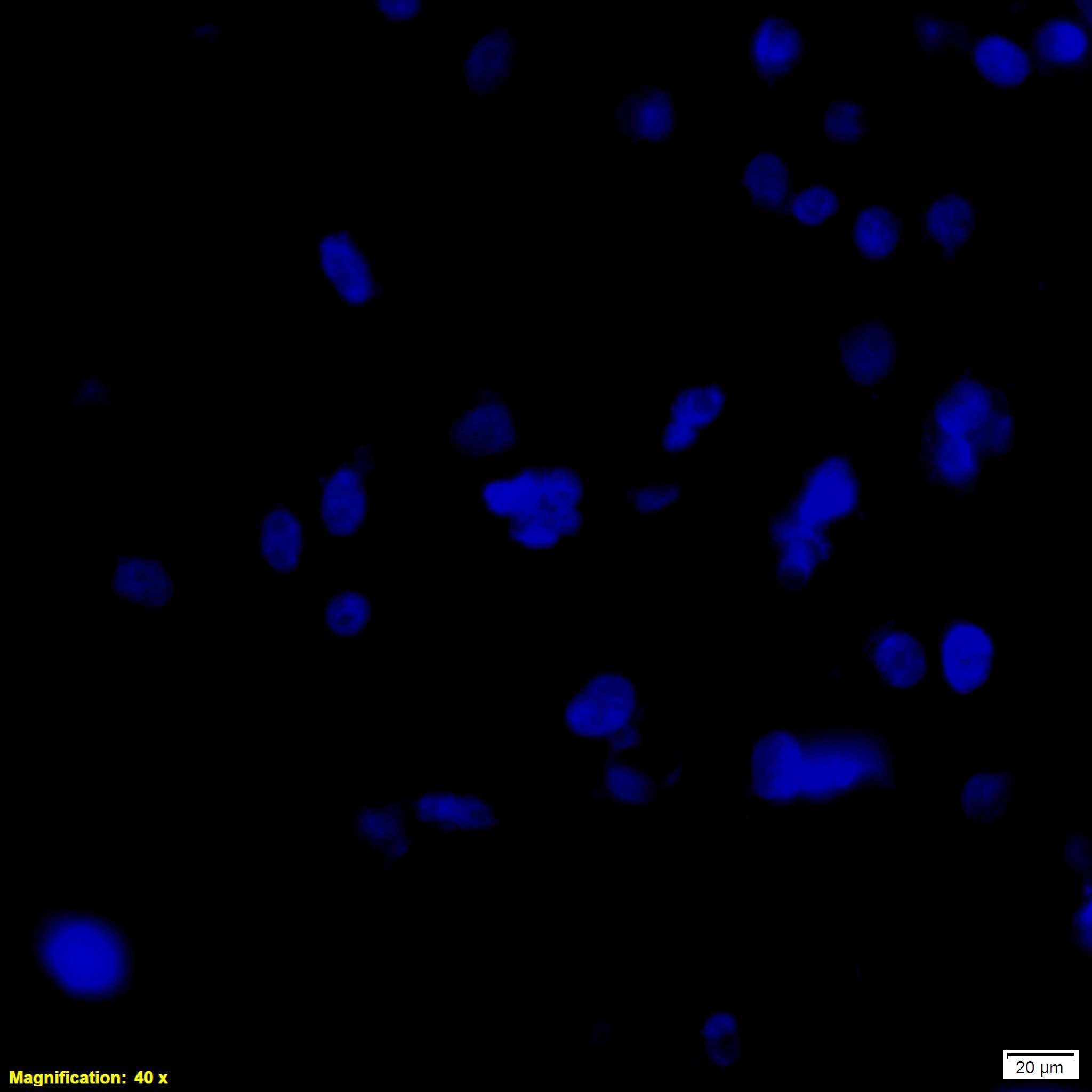

Supplement: Supplementary file 11 — Source data Fig. 5 [file 44318_2024_103_MOESM11_ESM.zip › Figure 5/5J/+ AKTi.jpg]
