# Supplementary figures and images for: Recycled melanoma-secreted melanosomes regulate tumor-associated macrophage diversification (part 2 of 2)
Source: EMBO J. 2024 May 8;43(17):3. doi: 10.1038/s44318-024-00103-7 (PMC11377571; doi:10.1038/s44318-024-00103-7)

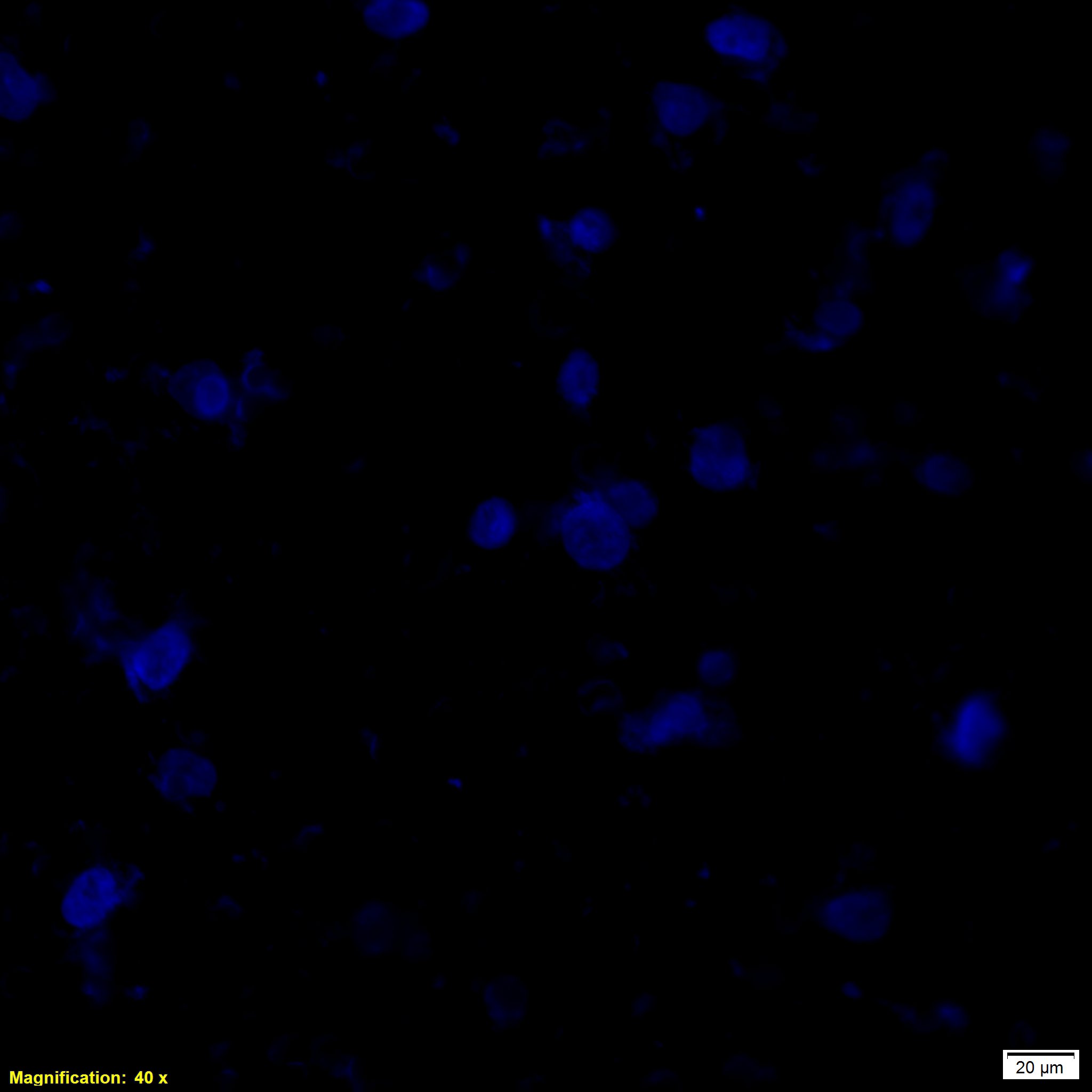

Supplement: Supplementary file 11 — Source data Fig. 5 [file 44318_2024_103_MOESM11_ESM.zip › Figure 5/5J/MNT1.jpg]

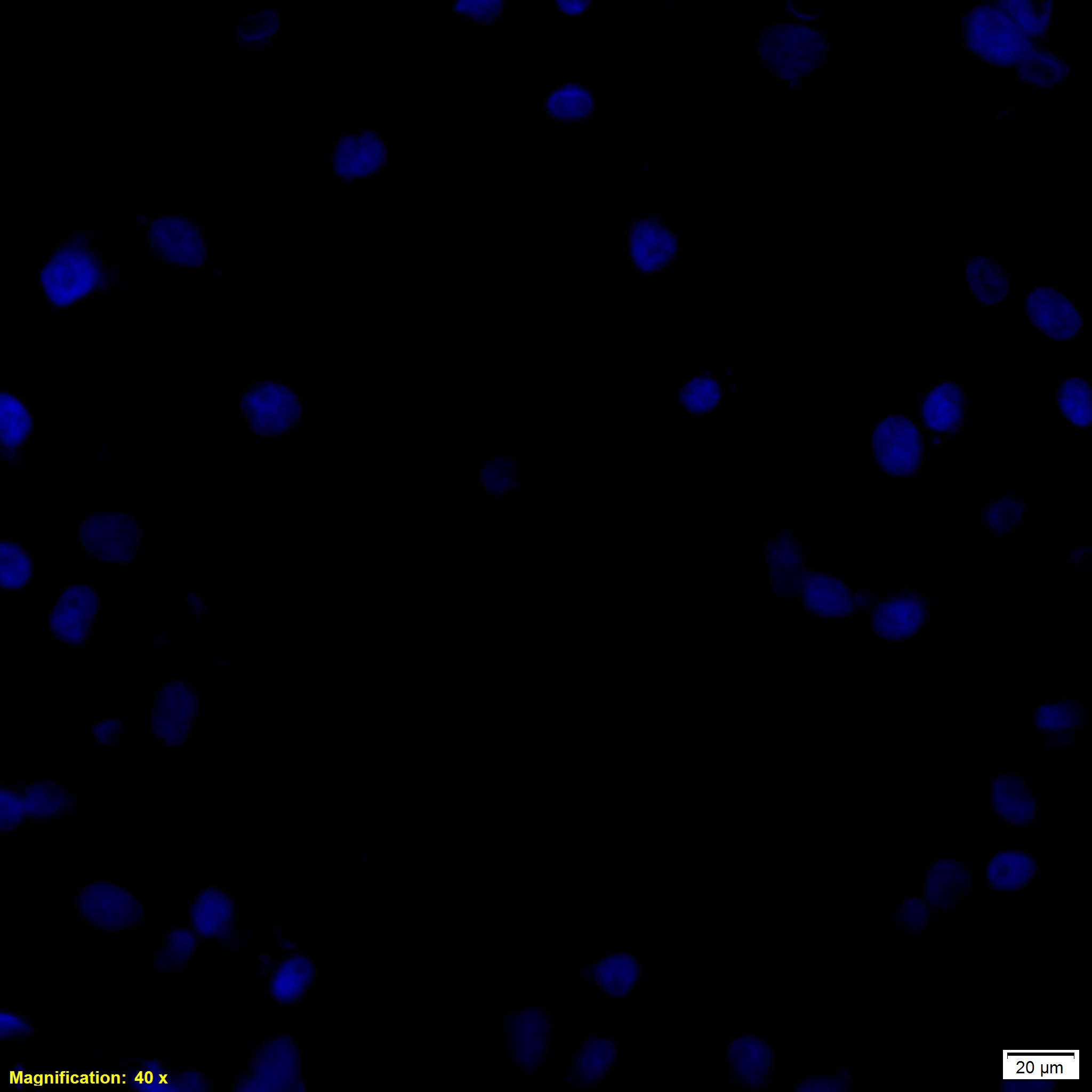

Supplement: Supplementary file 11 — Source data Fig. 5 [file 44318_2024_103_MOESM11_ESM.zip › Figure 5/5J/Naive.jpg]

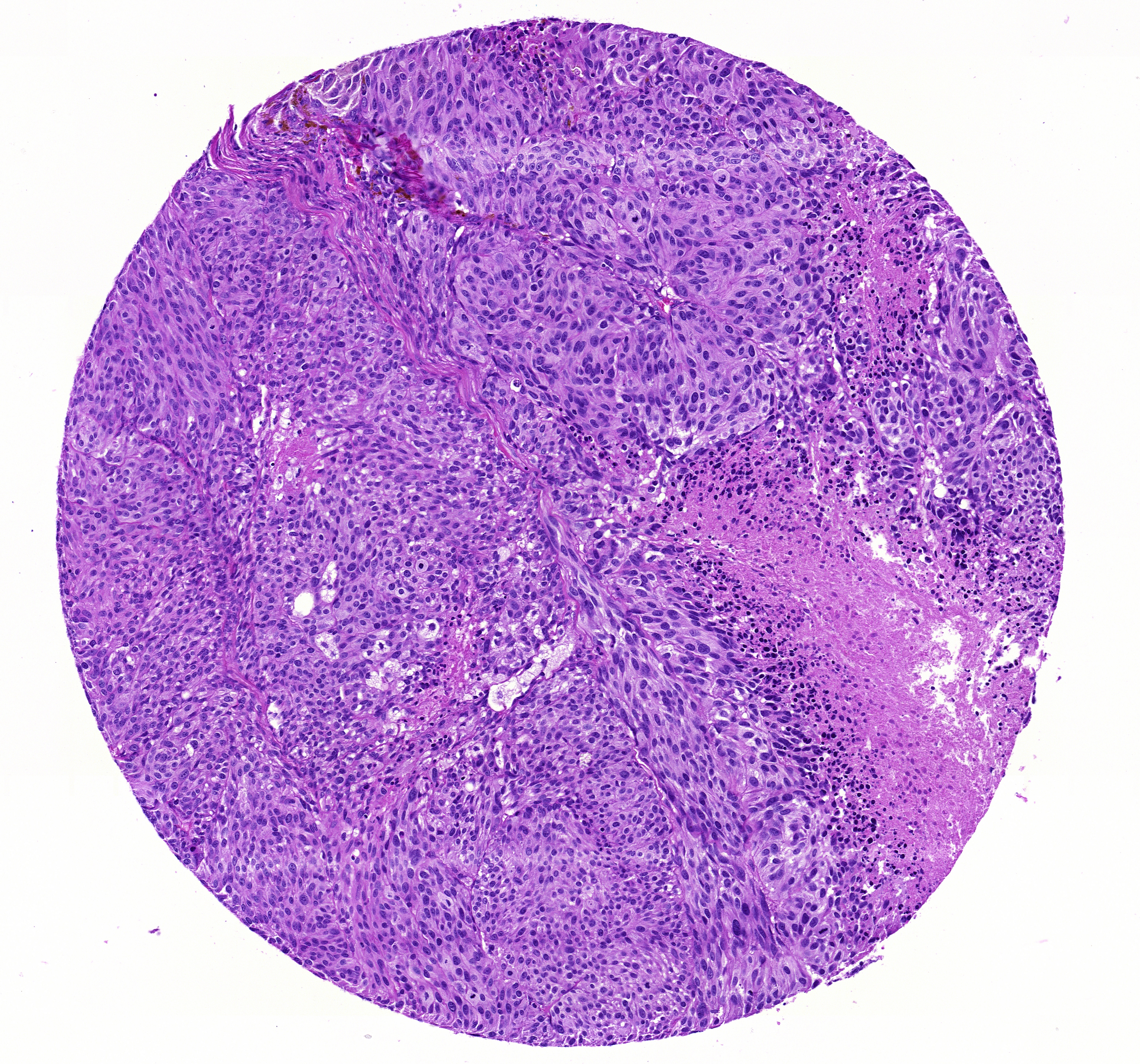

Supplement: Supplementary file 12 — Source data Fig. 6 [file 44318_2024_103_MOESM12_ESM.zip › Figure 6/6A/Non-responders_400um images/Image number 1.jpg]

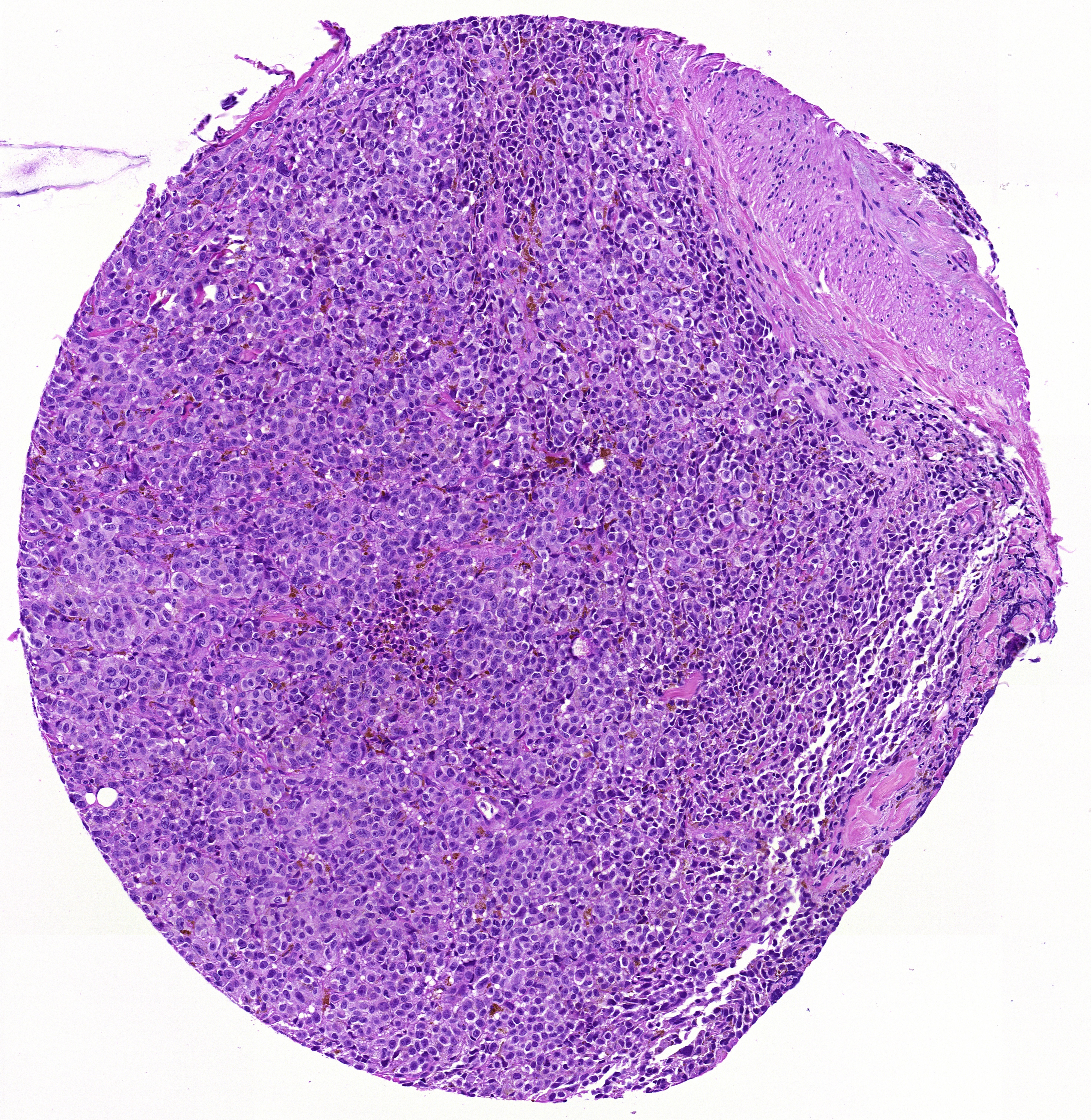

Supplement: Supplementary file 12 — Source data Fig. 6 [file 44318_2024_103_MOESM12_ESM.zip › Figure 6/6A/Non-responders_400um images/Image number 2.jpg]

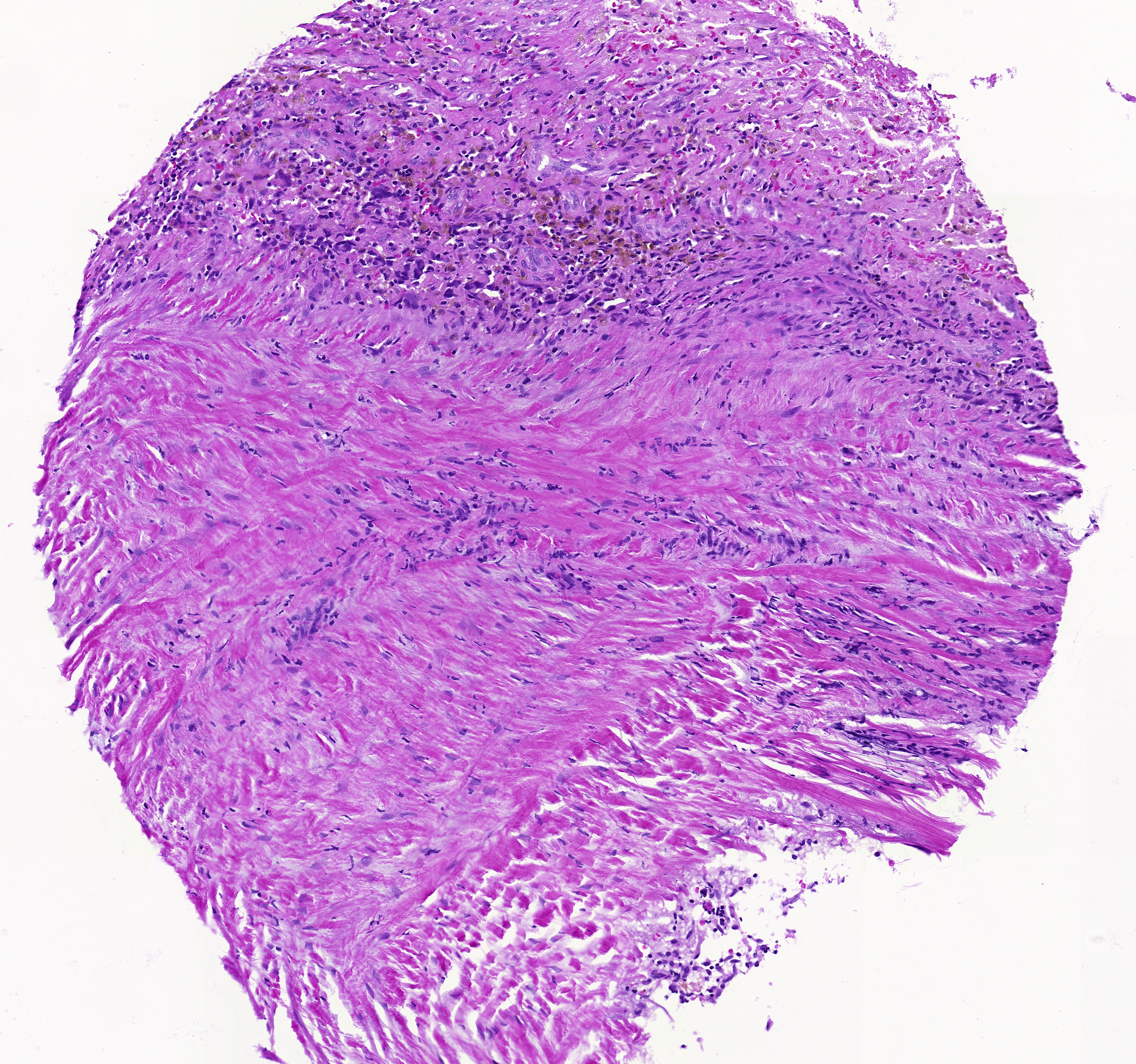

Supplement: Supplementary file 12 — Source data Fig. 6 [file 44318_2024_103_MOESM12_ESM.zip › Figure 6/6A/Non-responders_400um images/Image number 3.jpg]

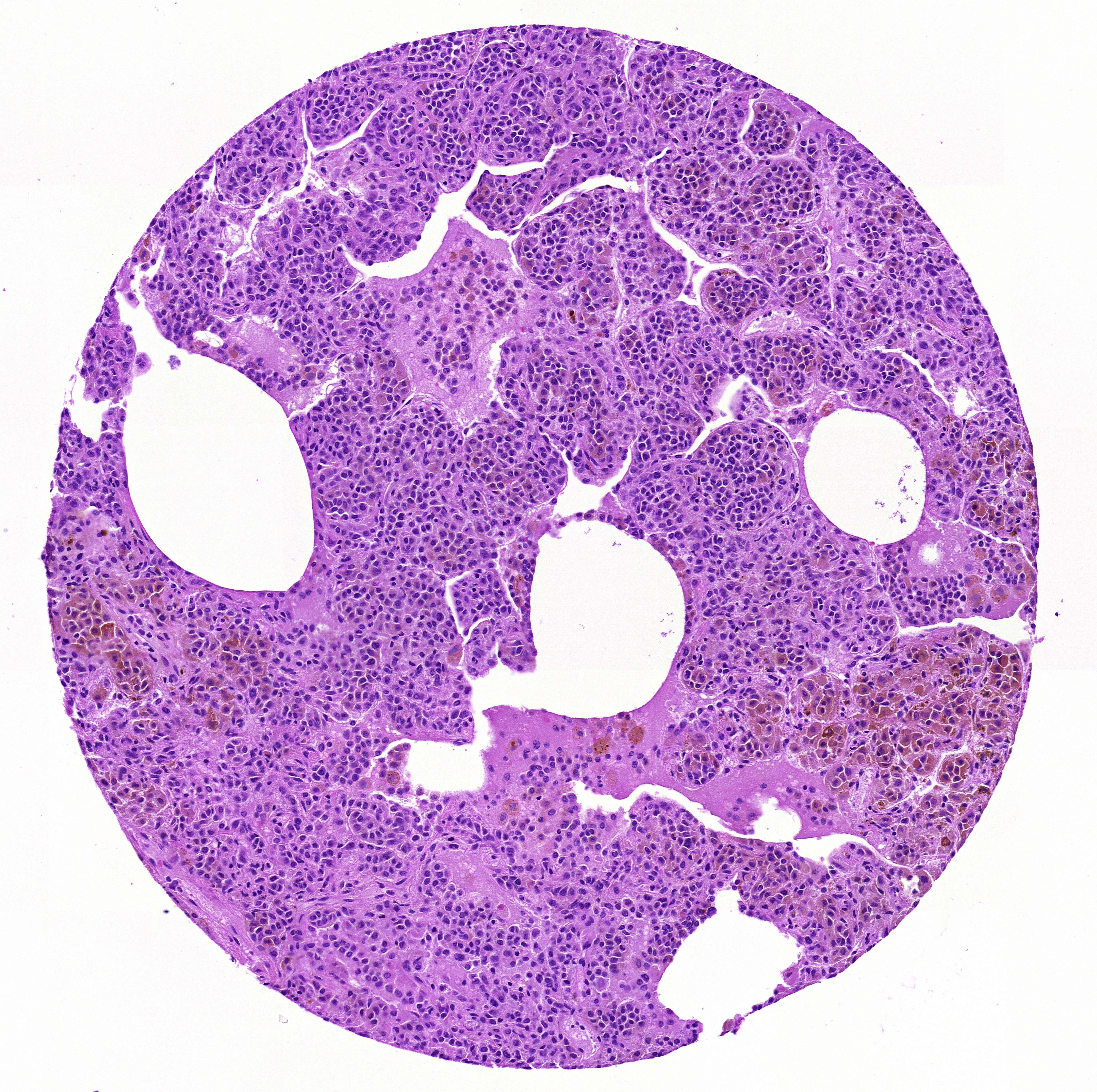

Supplement: Supplementary file 12 — Source data Fig. 6 [file 44318_2024_103_MOESM12_ESM.zip › Figure 6/6A/Non-responders_400um images/Image number 4.jpg]

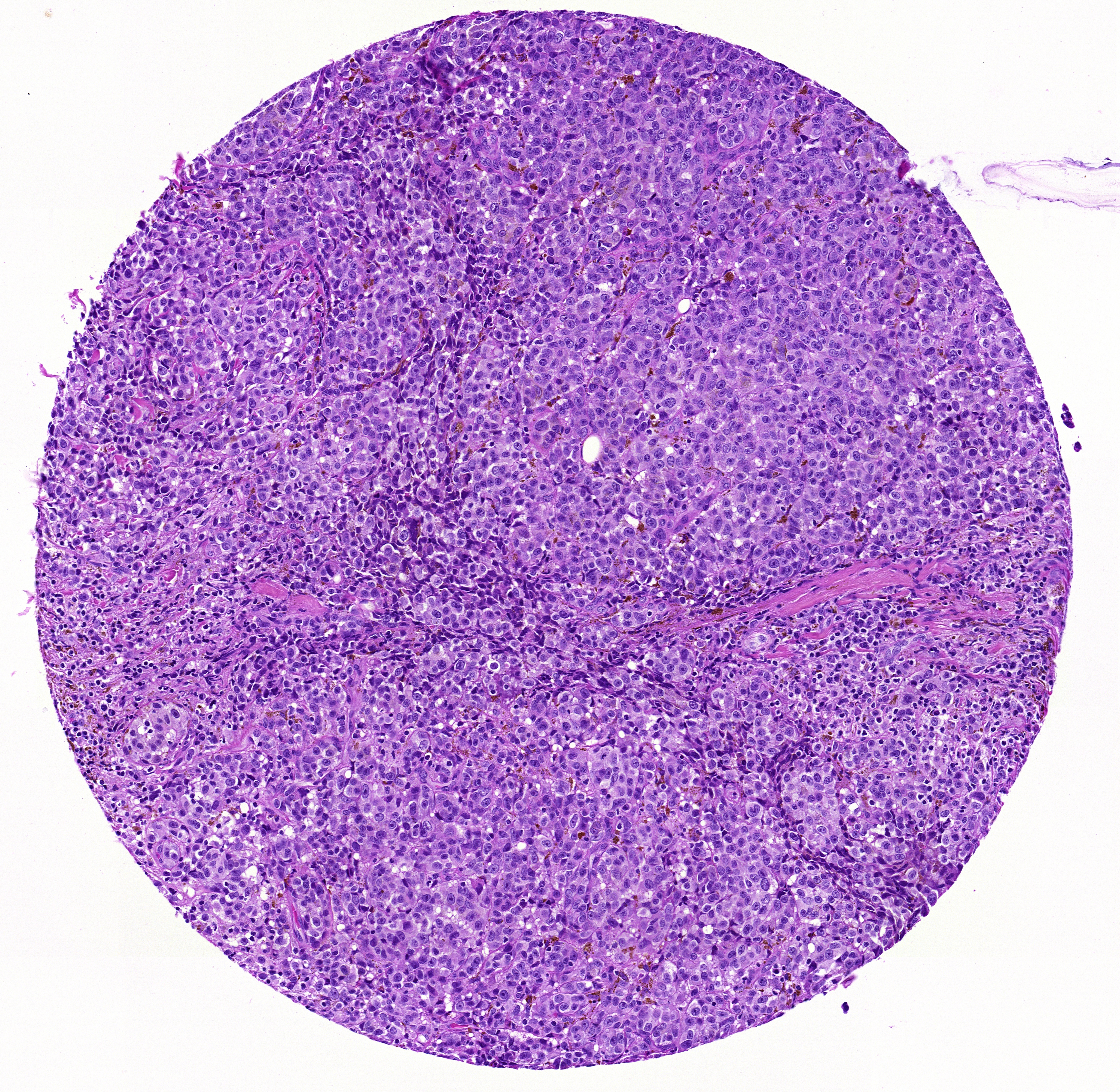

Supplement: Supplementary file 12 — Source data Fig. 6 [file 44318_2024_103_MOESM12_ESM.zip › Figure 6/6A/Non-responders_400um images/Image number 5.jpg]

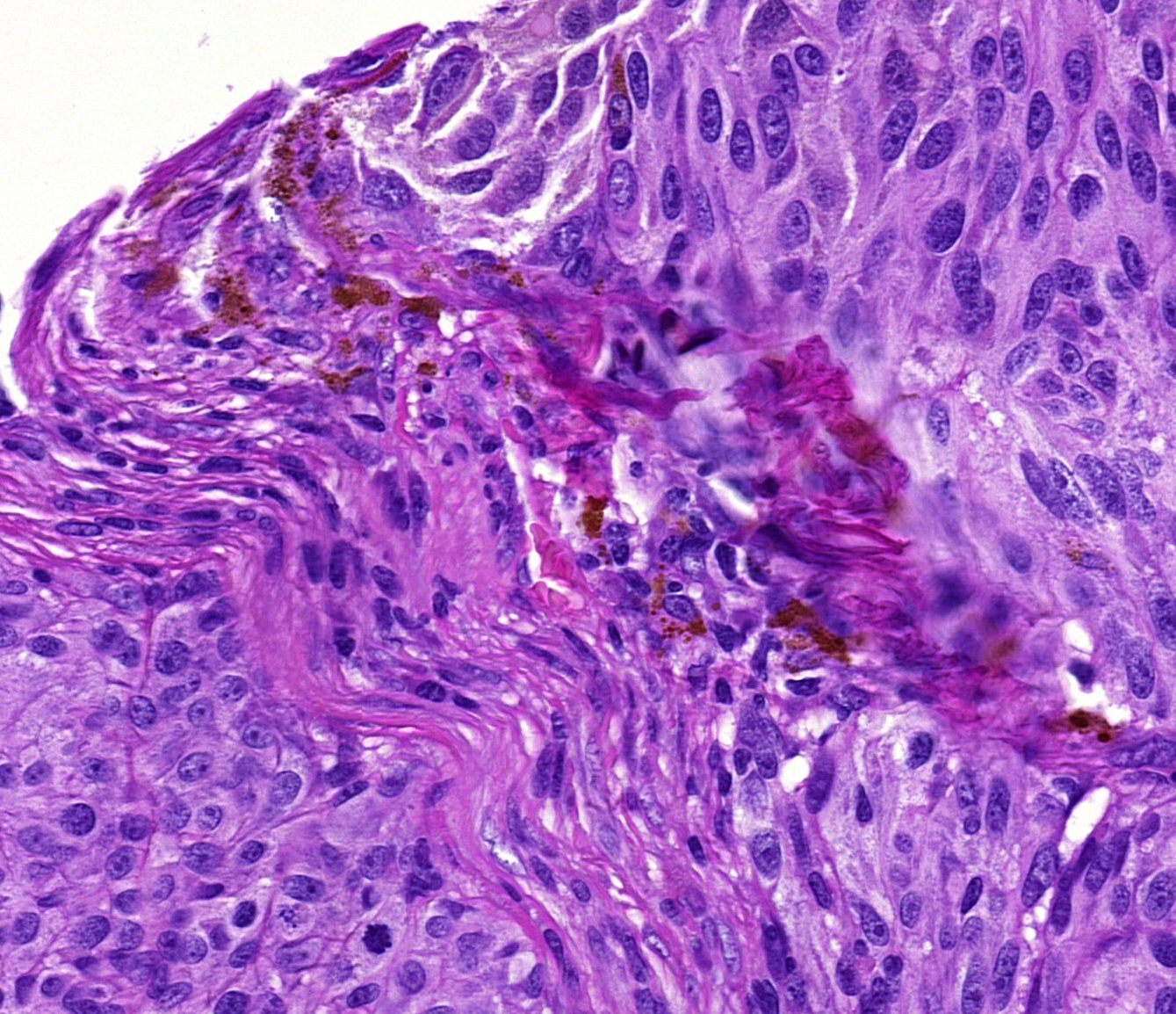

Supplement: Supplementary file 12 — Source data Fig. 6 [file 44318_2024_103_MOESM12_ESM.zip › Figure 6/6A/Non-responders_80um images/Image number 1.jpg]

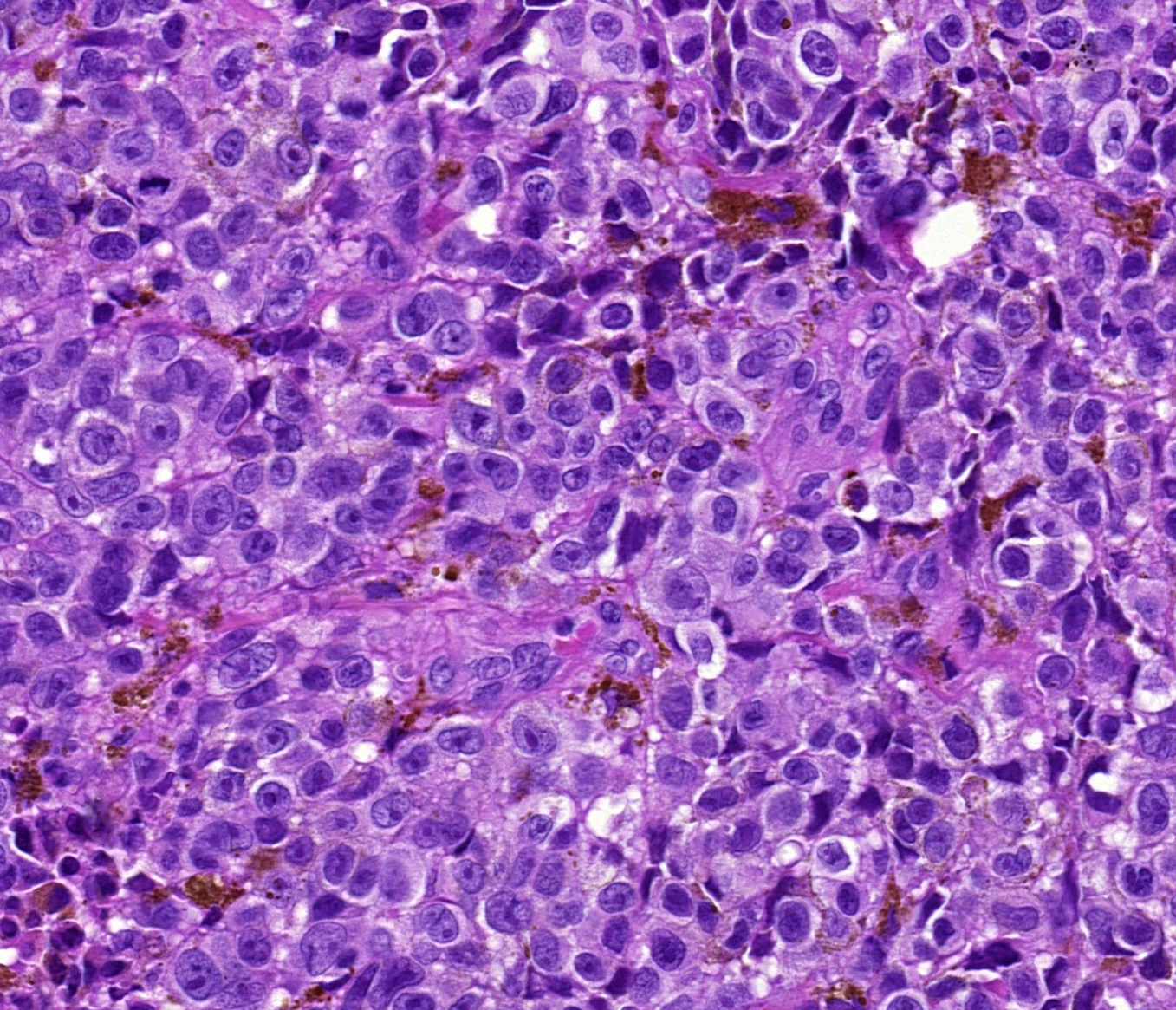

Supplement: Supplementary file 12 — Source data Fig. 6 [file 44318_2024_103_MOESM12_ESM.zip › Figure 6/6A/Non-responders_80um images/Image number 2.jpg]

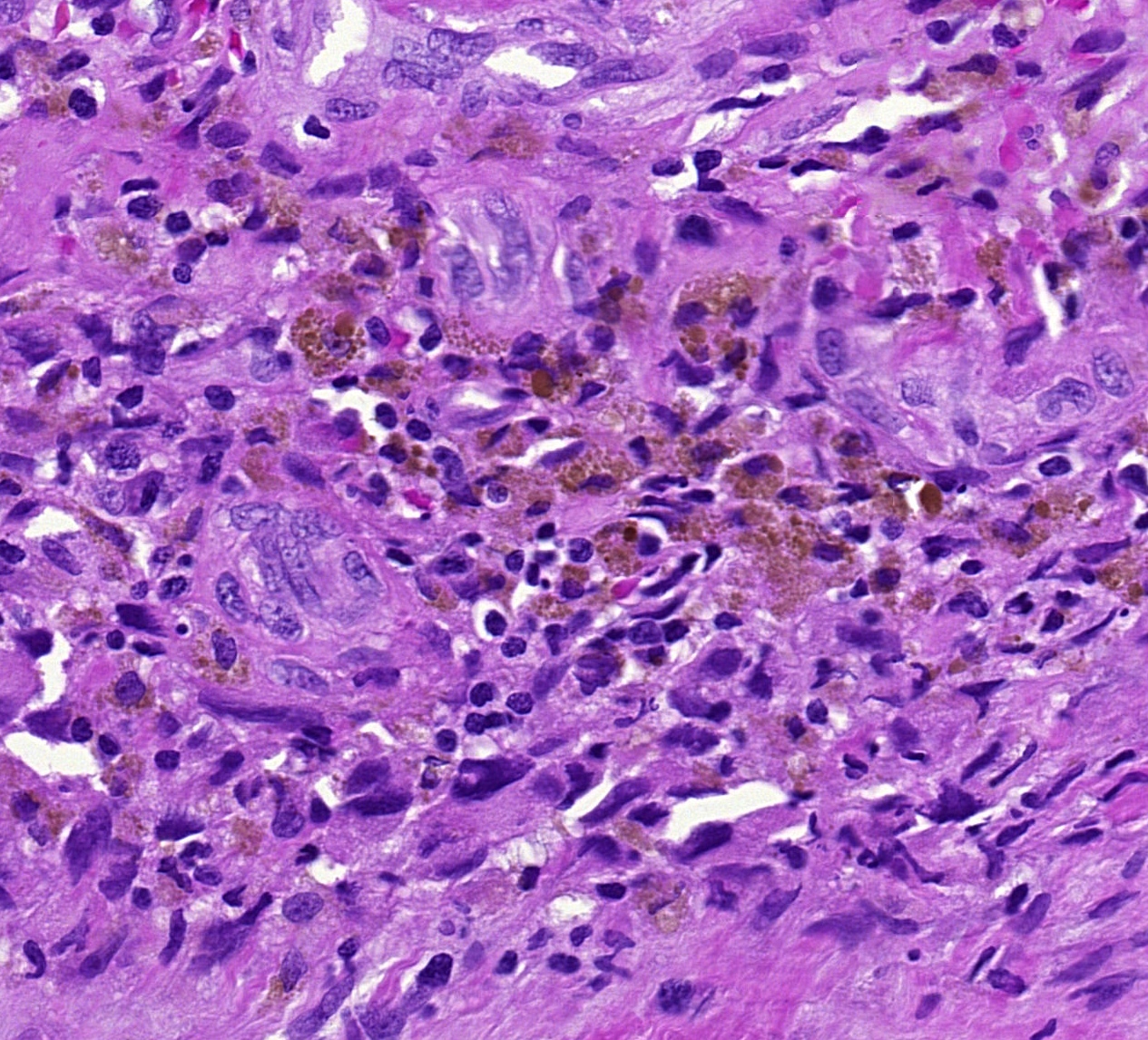

Supplement: Supplementary file 12 — Source data Fig. 6 [file 44318_2024_103_MOESM12_ESM.zip › Figure 6/6A/Non-responders_80um images/Image number 3.jpg]

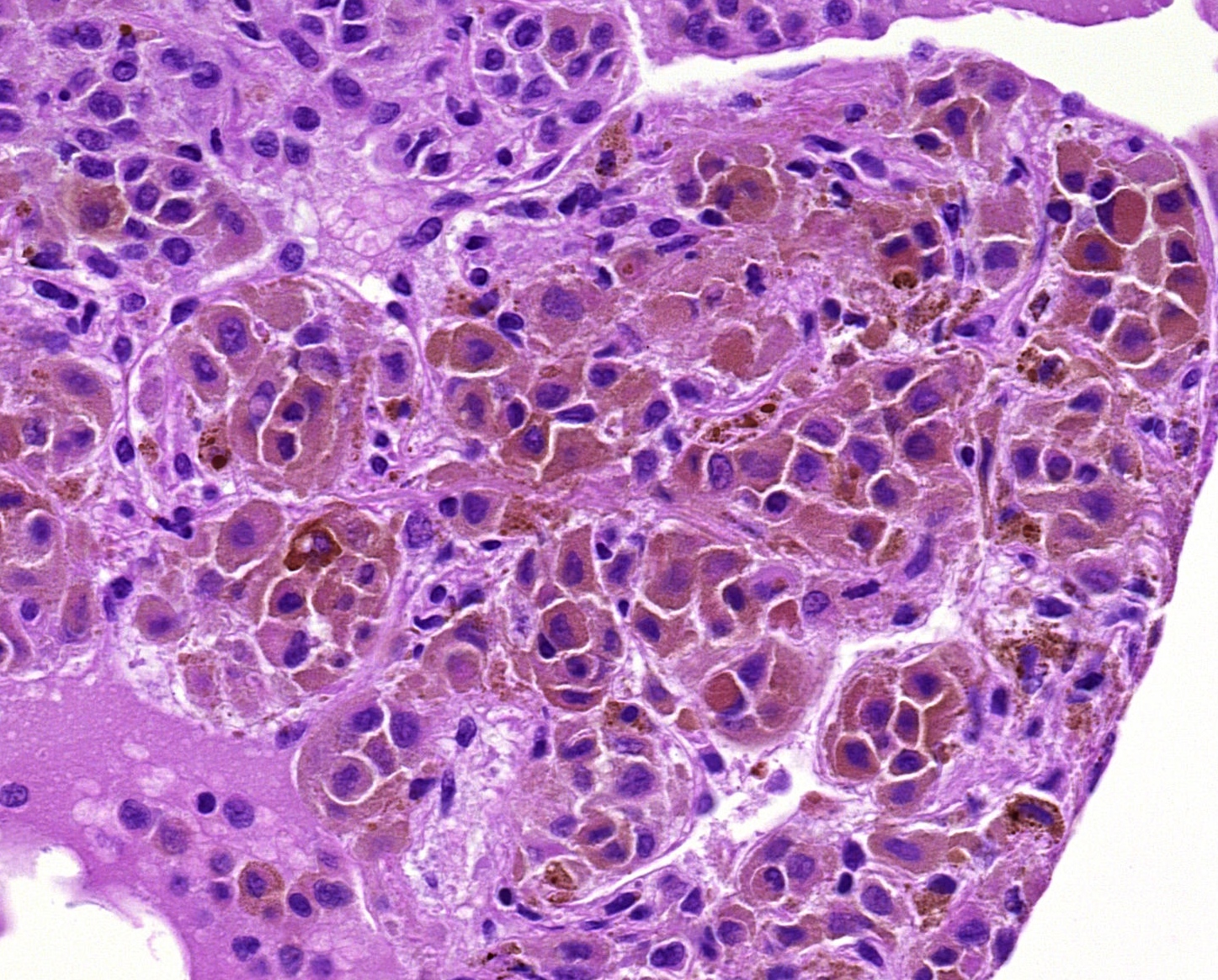

Supplement: Supplementary file 12 — Source data Fig. 6 [file 44318_2024_103_MOESM12_ESM.zip › Figure 6/6A/Non-responders_80um images/Image number 4.jpg]

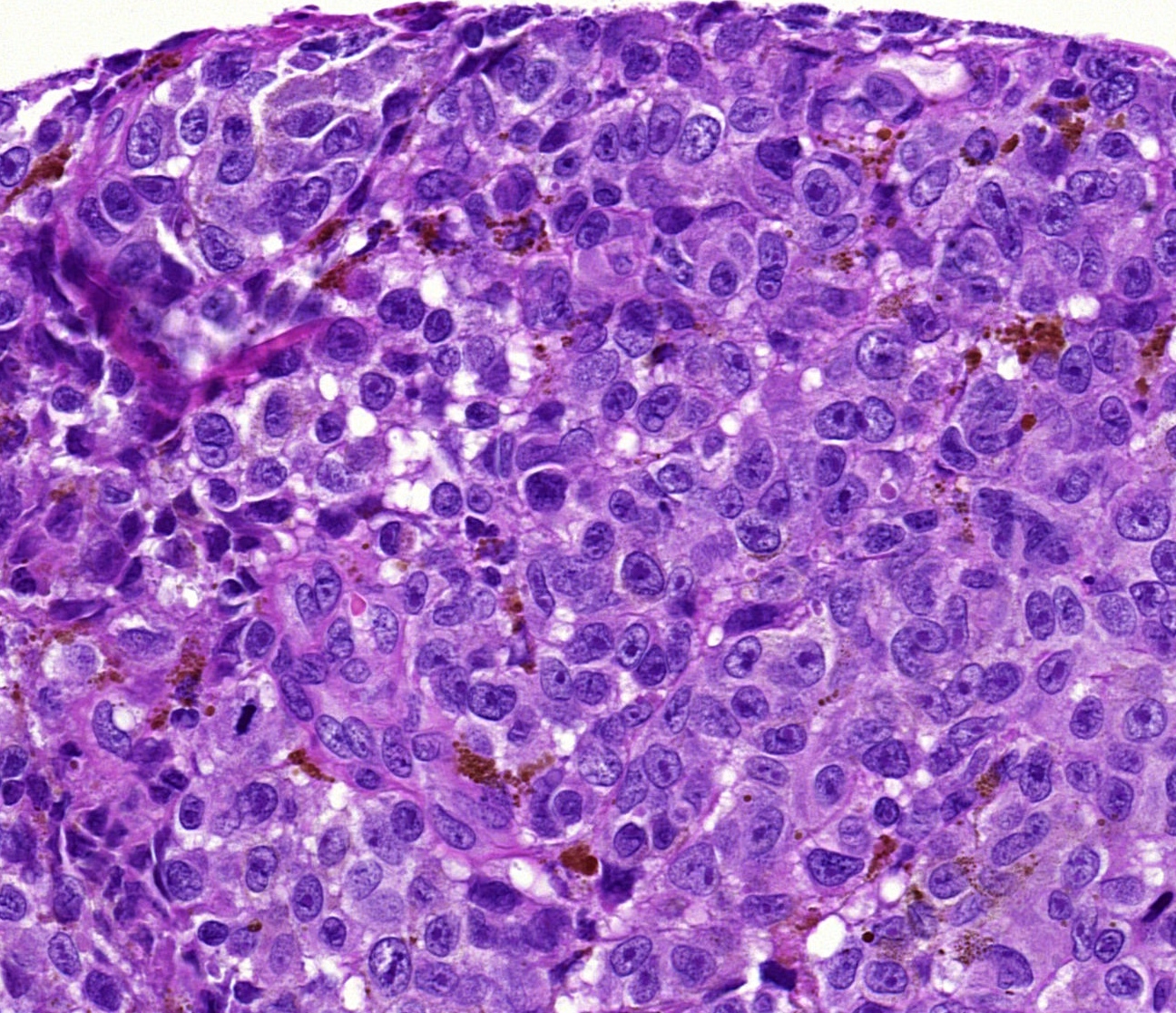

Supplement: Supplementary file 12 — Source data Fig. 6 [file 44318_2024_103_MOESM12_ESM.zip › Figure 6/6A/Non-responders_80um images/Image number 5.jpg]

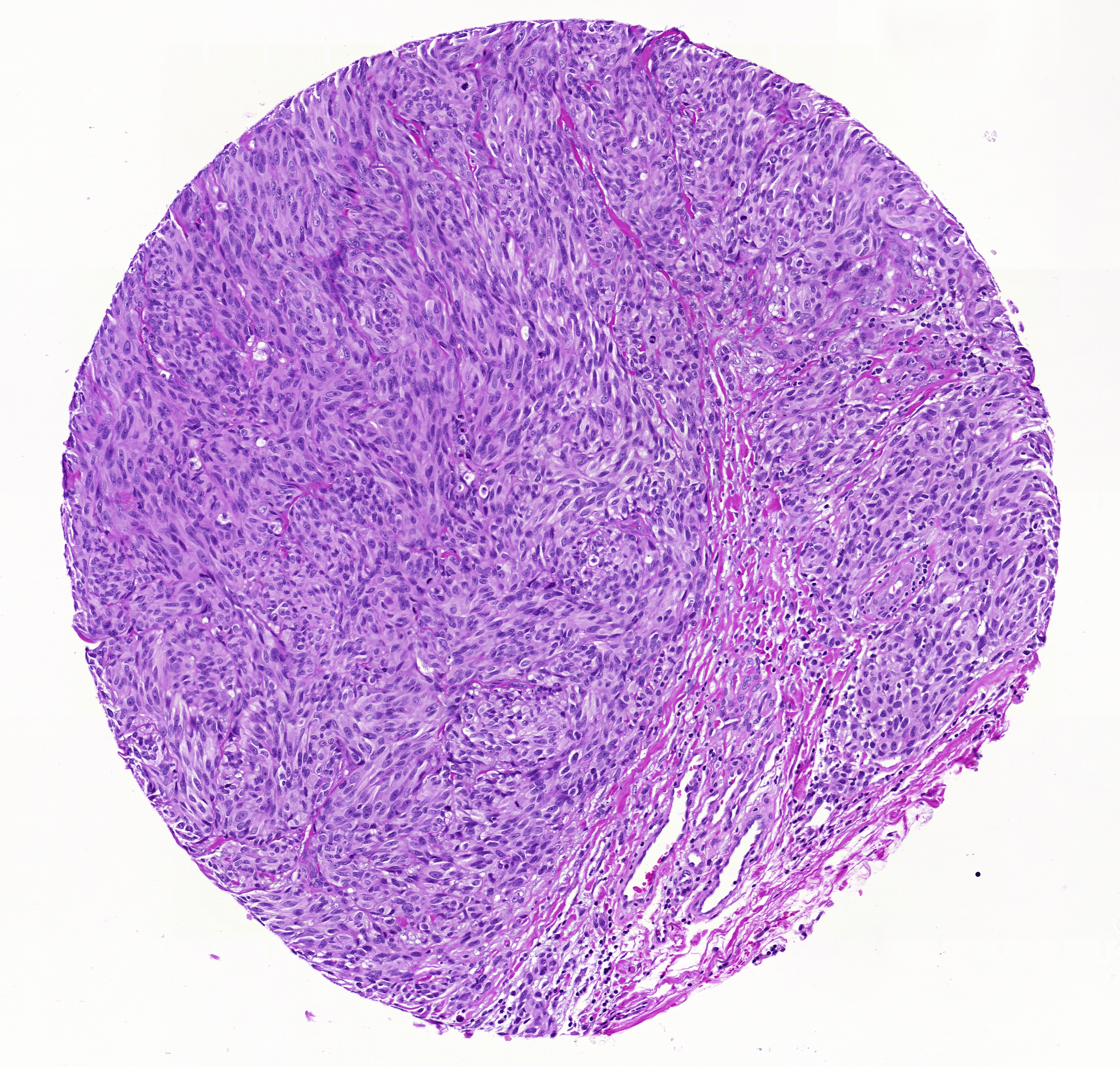

Supplement: Supplementary file 12 — Source data Fig. 6 [file 44318_2024_103_MOESM12_ESM.zip › Figure 6/6A/Reponders_400um images/Image number 1.jpg]

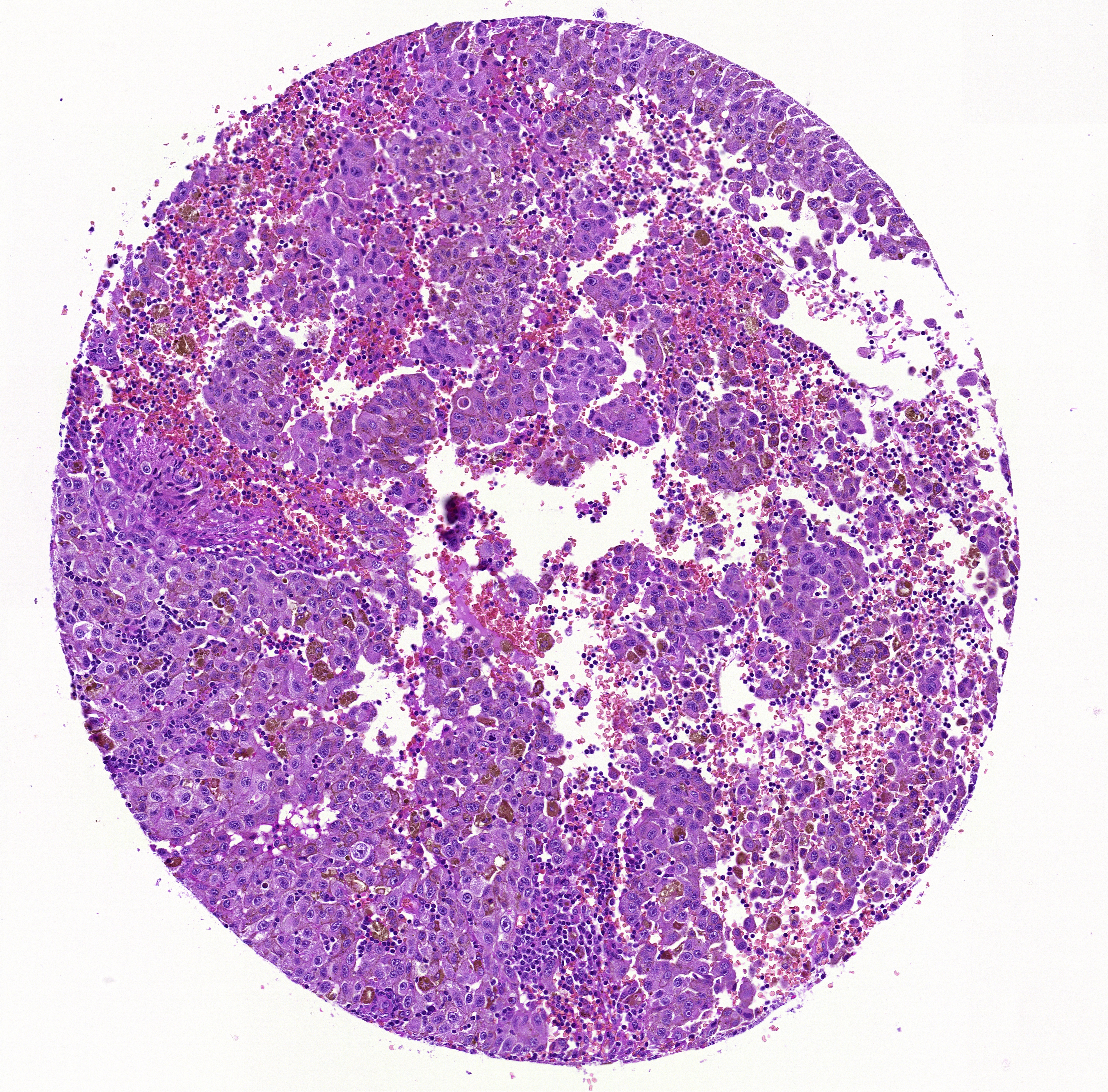

Supplement: Supplementary file 12 — Source data Fig. 6 [file 44318_2024_103_MOESM12_ESM.zip › Figure 6/6A/Reponders_400um images/Image number 2.jpg]

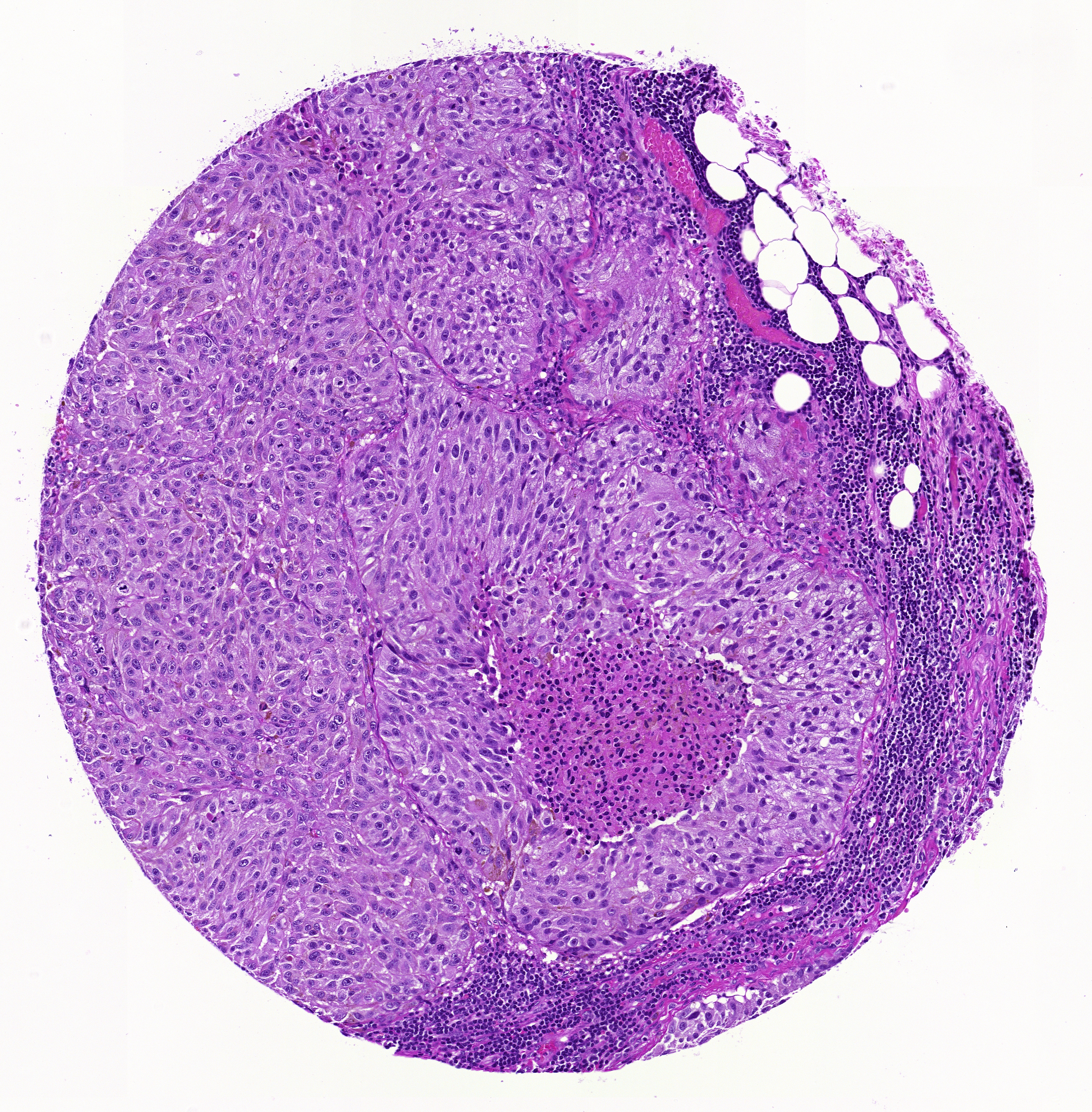

Supplement: Supplementary file 12 — Source data Fig. 6 [file 44318_2024_103_MOESM12_ESM.zip › Figure 6/6A/Reponders_400um images/Image number 3.jpg]

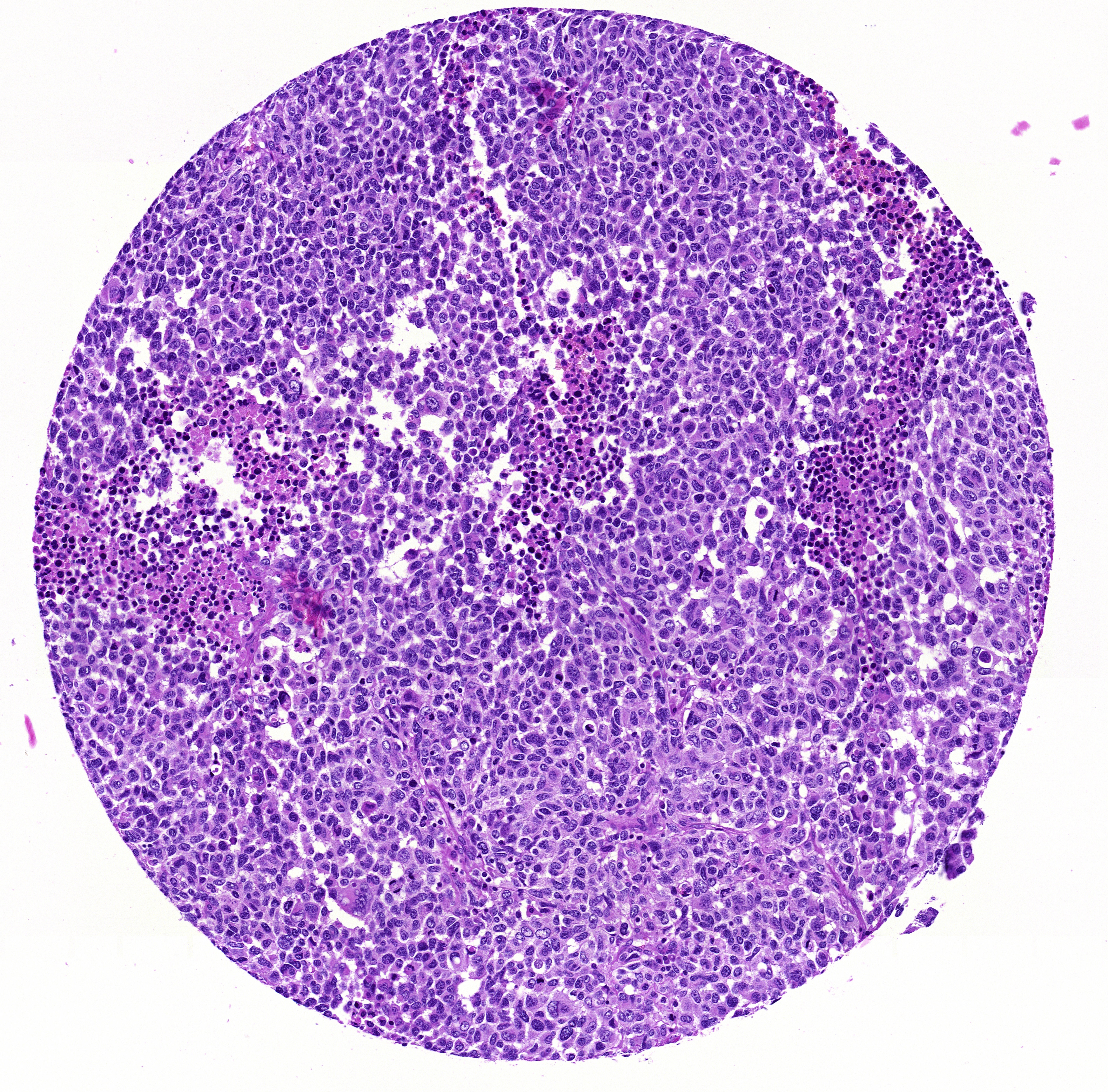

Supplement: Supplementary file 12 — Source data Fig. 6 [file 44318_2024_103_MOESM12_ESM.zip › Figure 6/6A/Reponders_400um images/Image number 4.jpg]

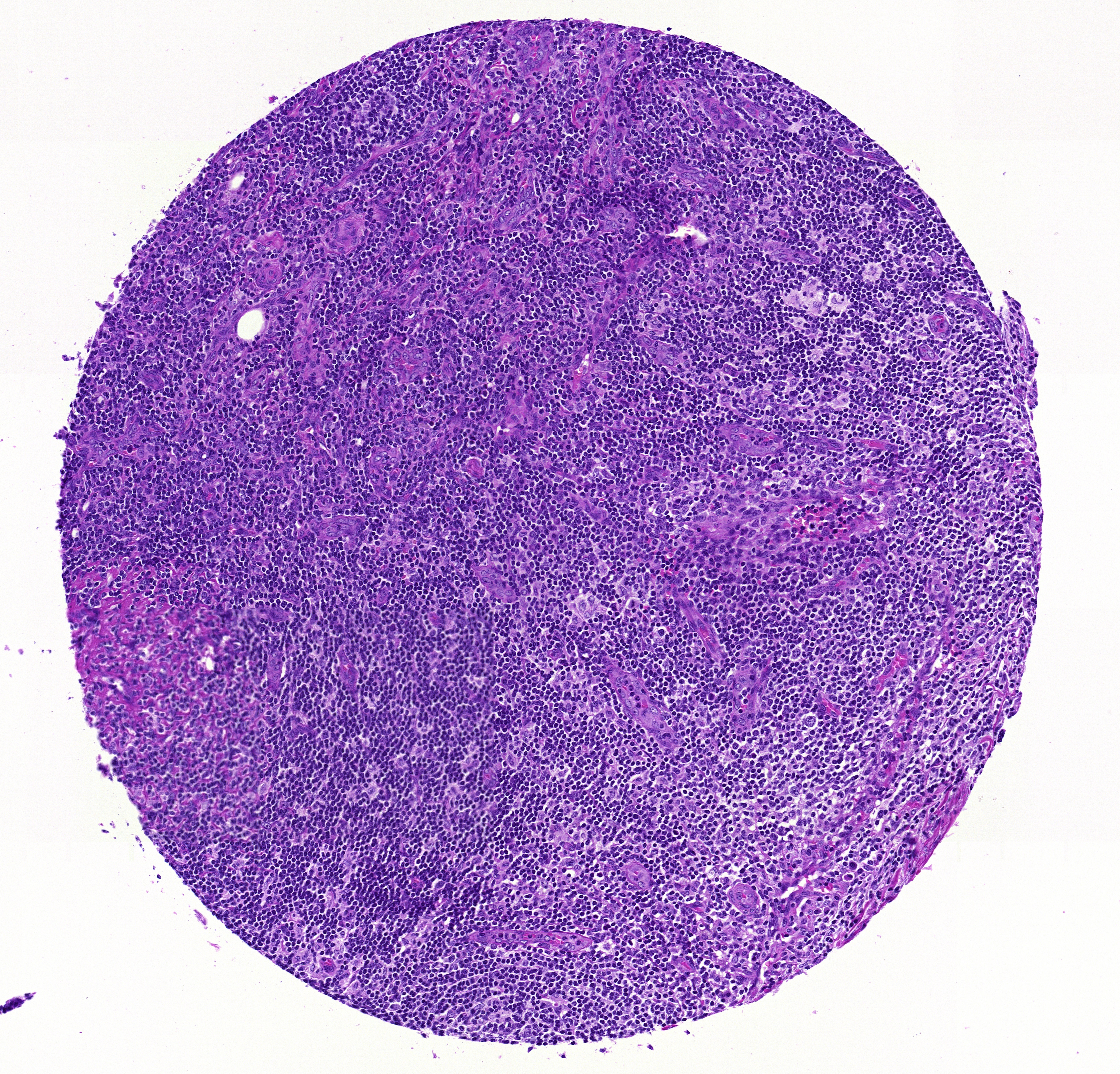

Supplement: Supplementary file 12 — Source data Fig. 6 [file 44318_2024_103_MOESM12_ESM.zip › Figure 6/6A/Reponders_400um images/Image number 5.jpg]

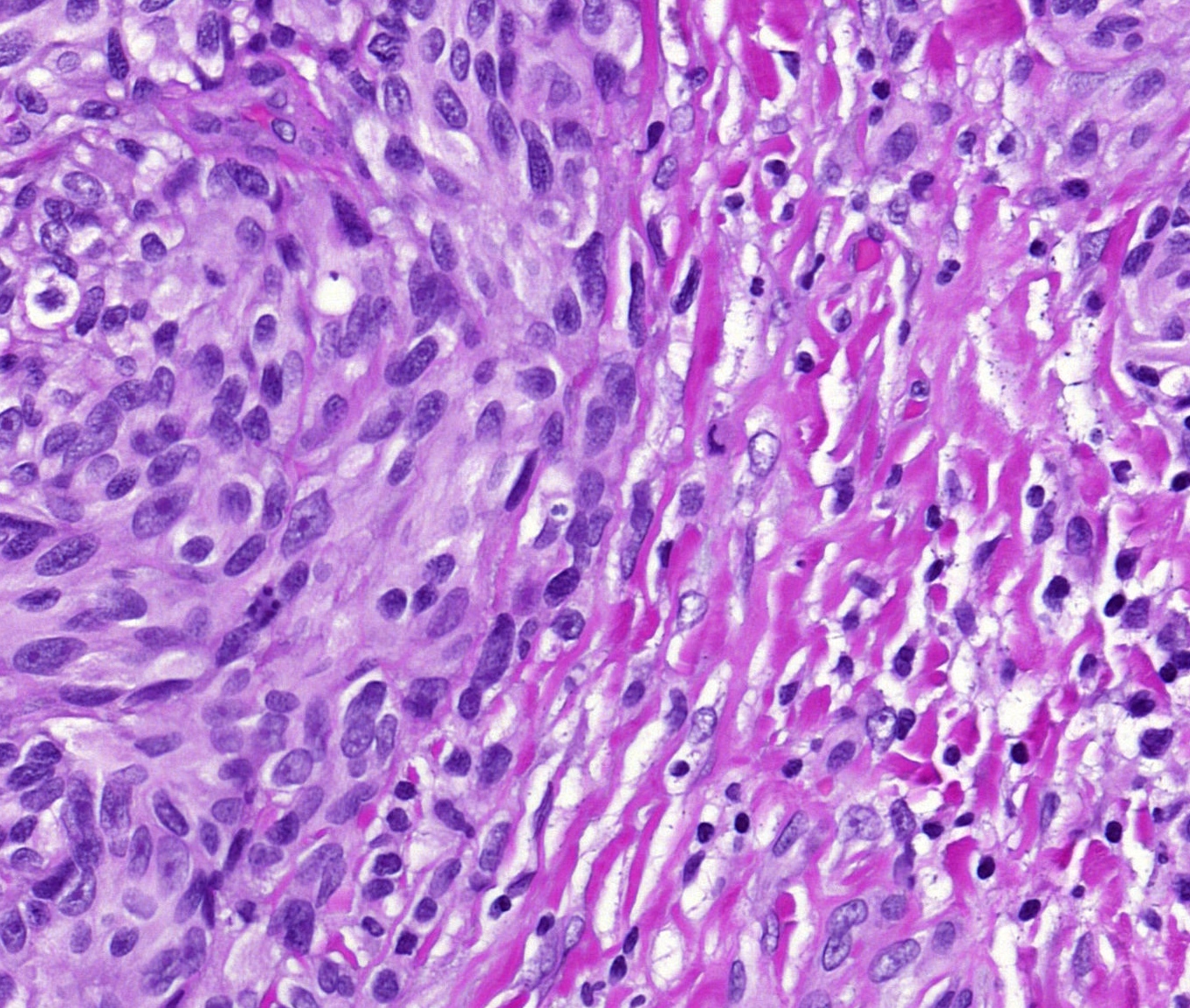

Supplement: Supplementary file 12 — Source data Fig. 6 [file 44318_2024_103_MOESM12_ESM.zip › Figure 6/6A/Reponders_80um images/Image number 1.jpg]

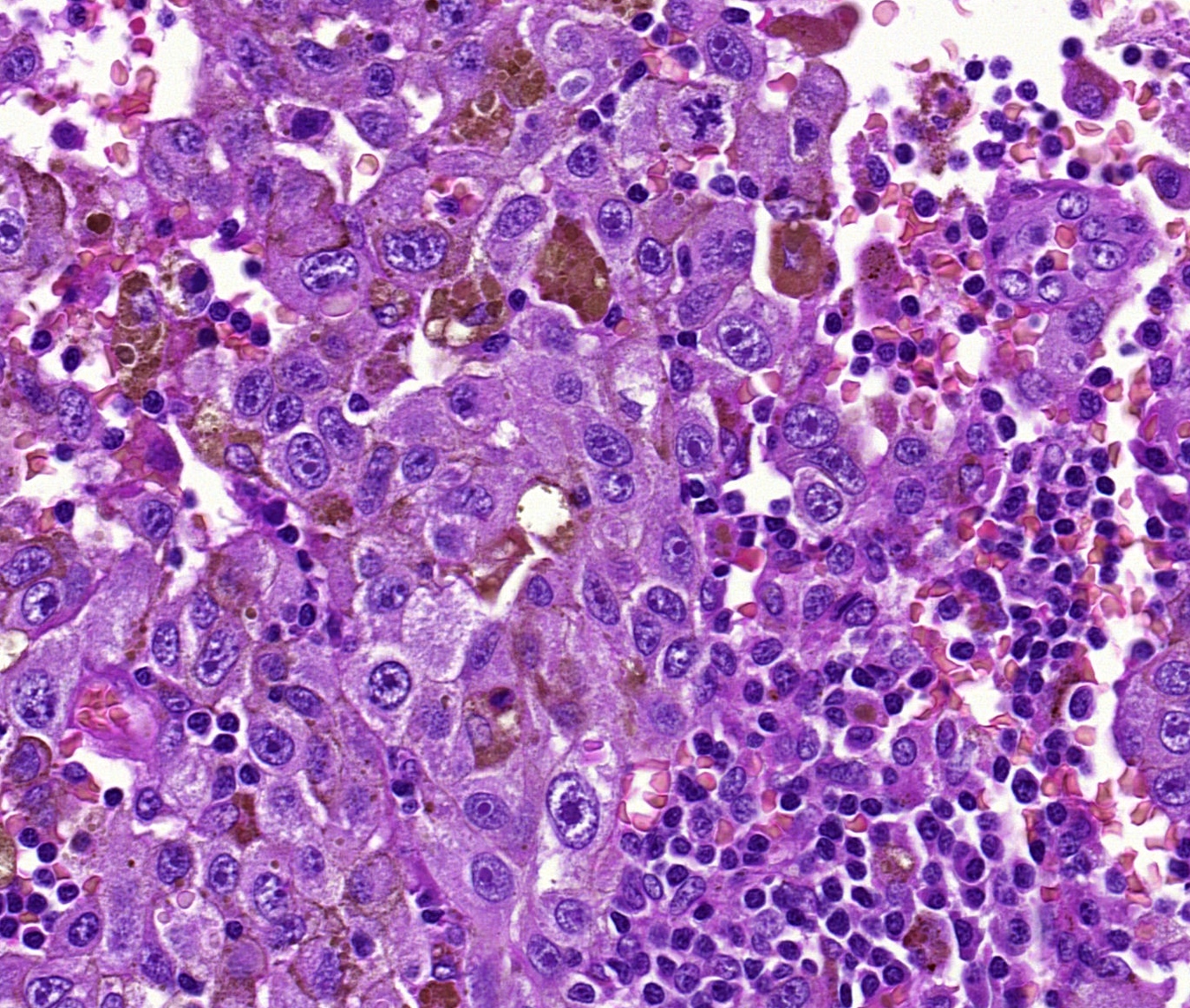

Supplement: Supplementary file 12 — Source data Fig. 6 [file 44318_2024_103_MOESM12_ESM.zip › Figure 6/6A/Reponders_80um images/Image number 2.jpg]

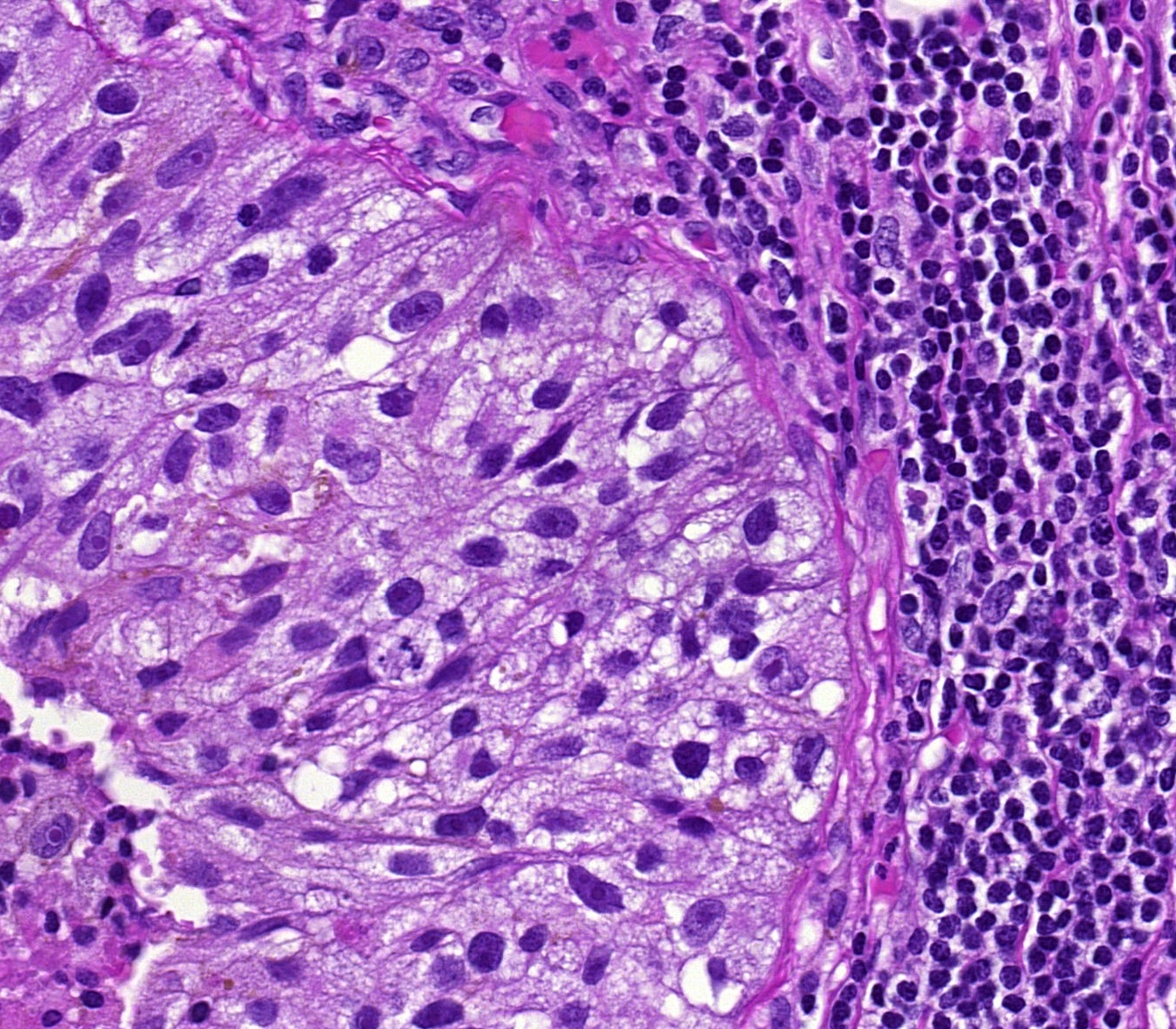

Supplement: Supplementary file 12 — Source data Fig. 6 [file 44318_2024_103_MOESM12_ESM.zip › Figure 6/6A/Reponders_80um images/Image number 3.jpg]

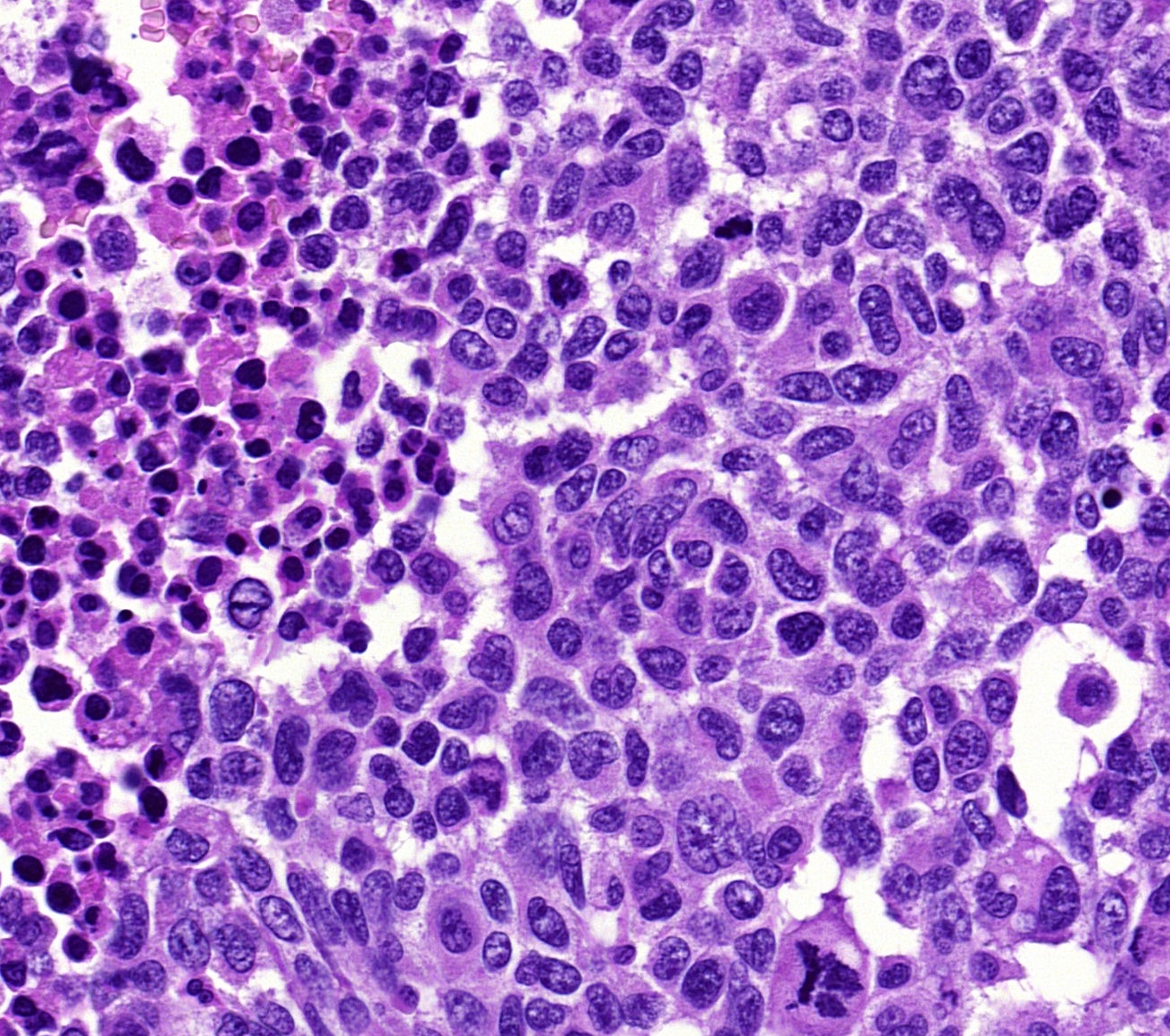

Supplement: Supplementary file 12 — Source data Fig. 6 [file 44318_2024_103_MOESM12_ESM.zip › Figure 6/6A/Reponders_80um images/Image number 4.jpg]

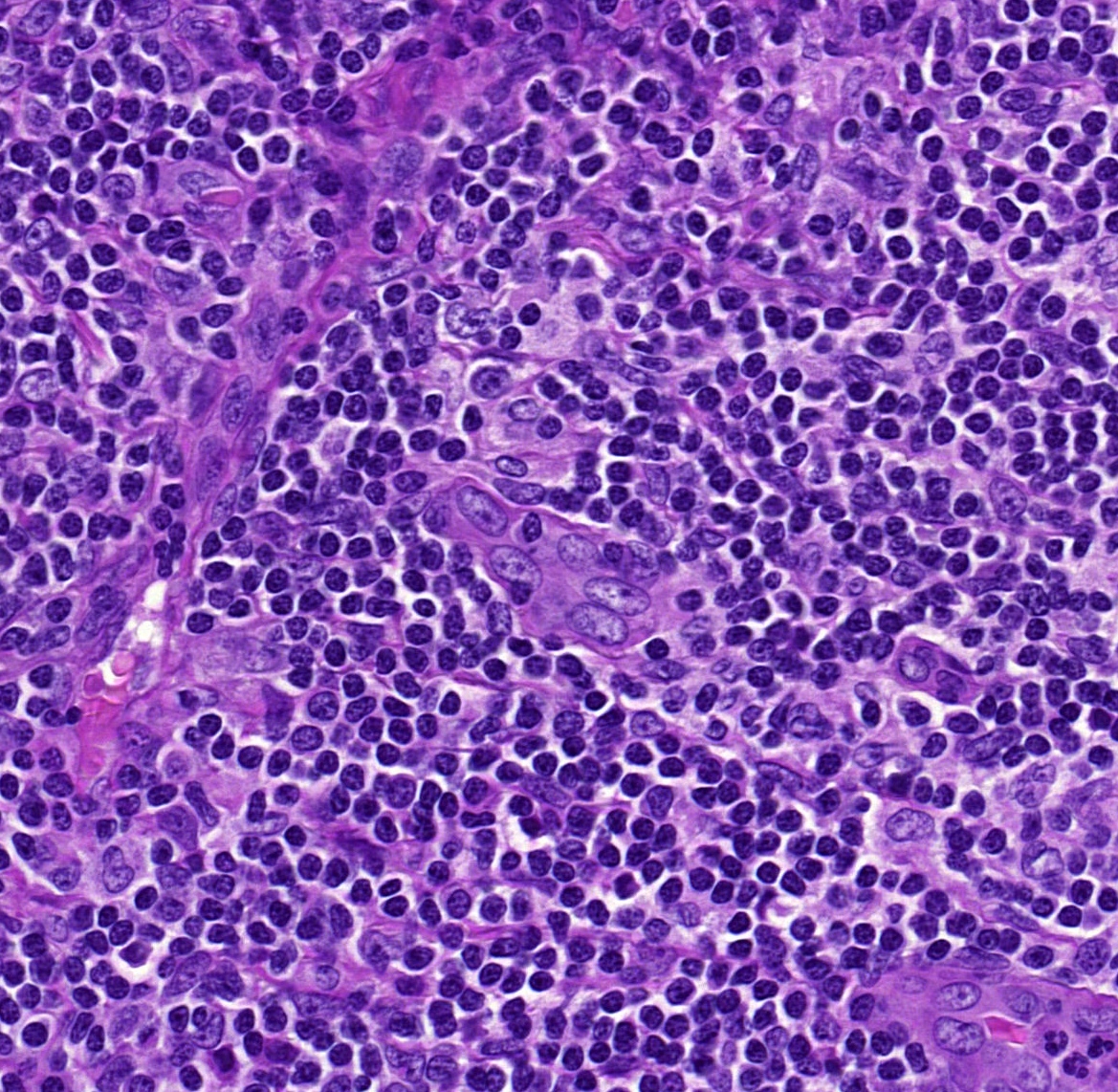

Supplement: Supplementary file 12 — Source data Fig. 6 [file 44318_2024_103_MOESM12_ESM.zip › Figure 6/6A/Reponders_80um images/Image number 5.jpg]

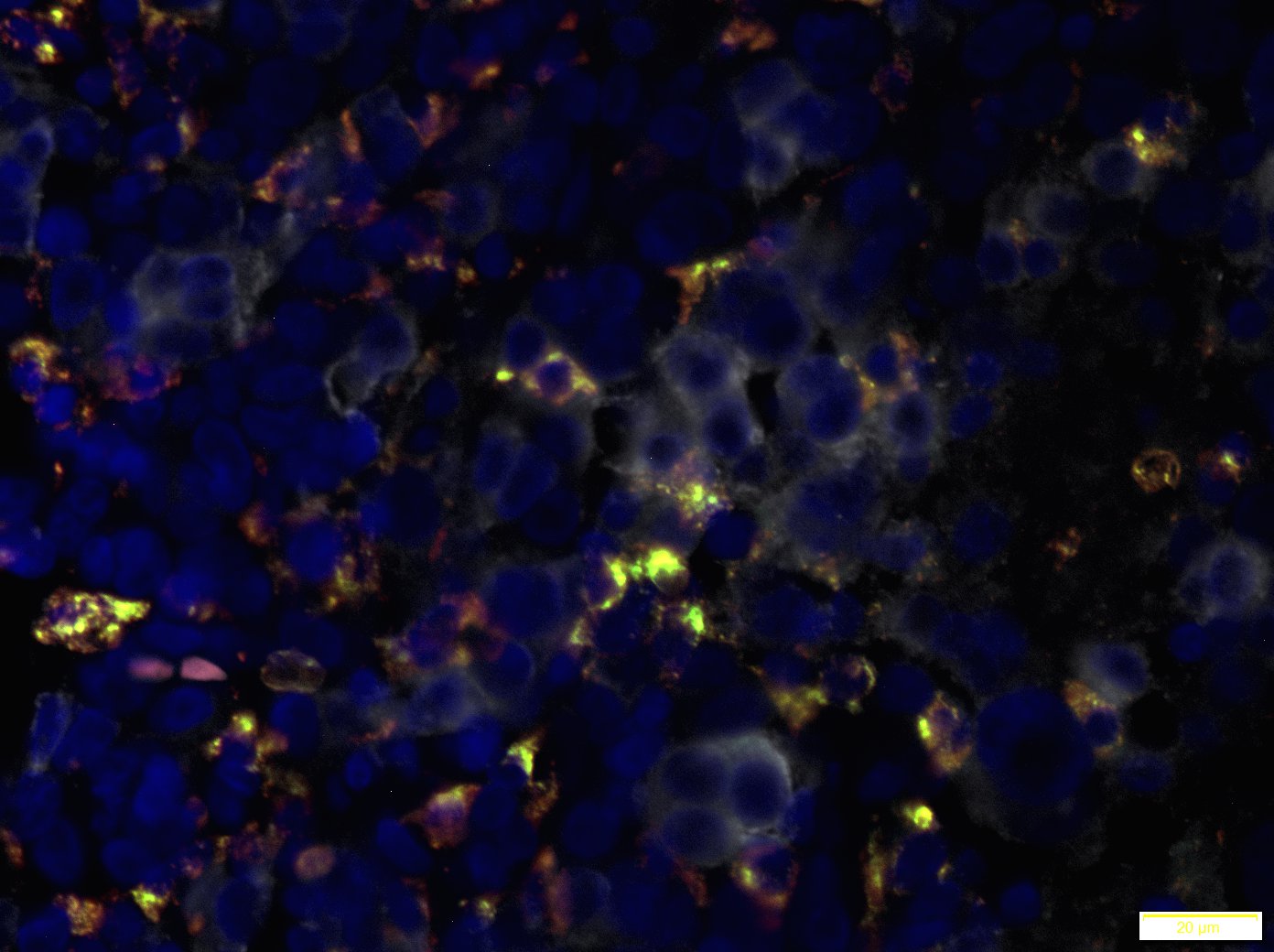

Supplement: Supplementary file 12 — Source data Fig. 6 [file 44318_2024_103_MOESM12_ESM.zip › Figure 6/6G/Tumor Lymph.jpg]

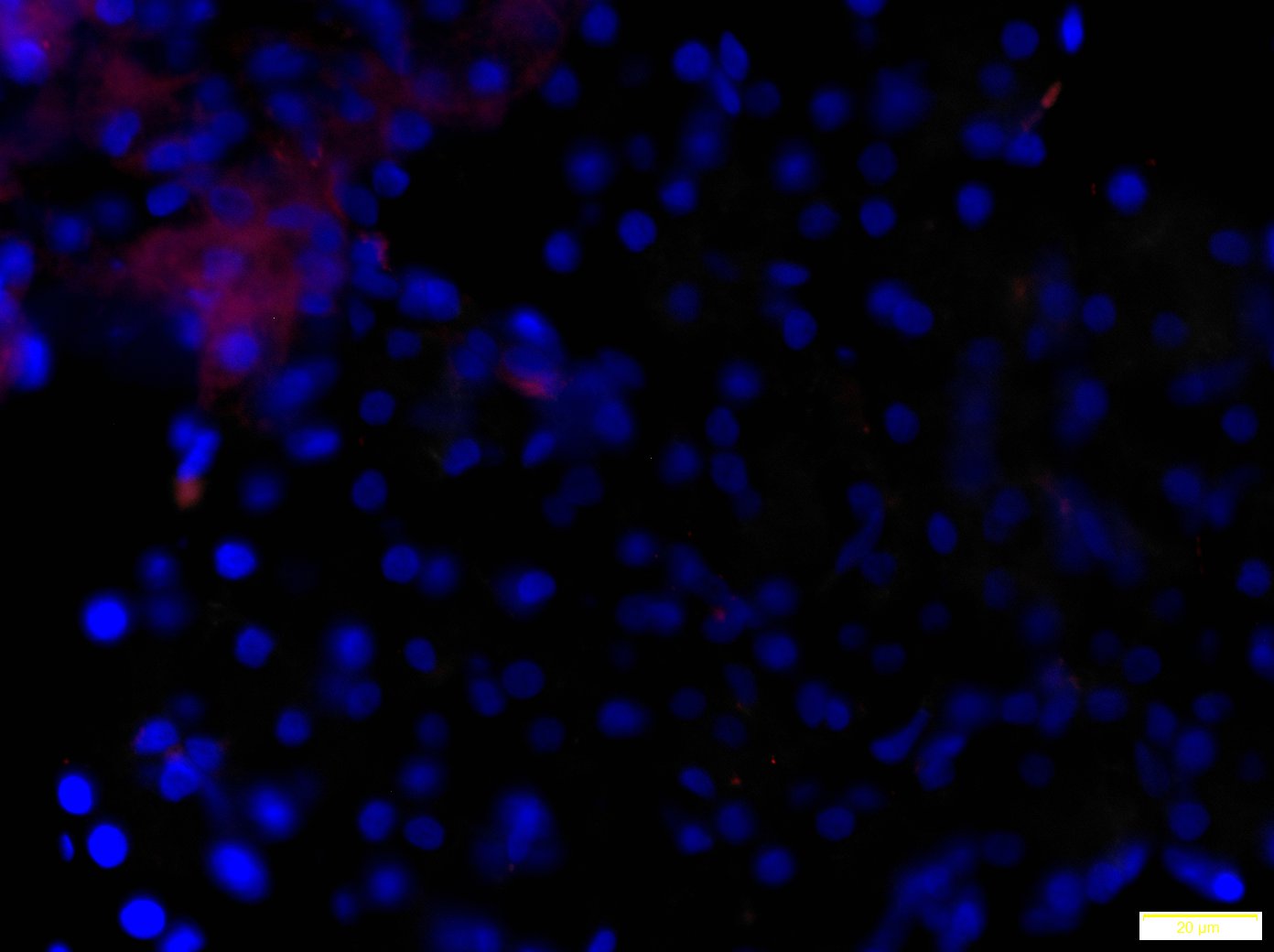

Supplement: Supplementary file 12 — Source data Fig. 6 [file 44318_2024_103_MOESM12_ESM.zip › Figure 6/6G/Tumor-Free Lymph.jpg]
