# Supplementary material for: Recycled melanoma-secreted melanosomes regulate tumor-associated macrophage diversification
Source: EMBO J. 2024 May 8;43(17):3. doi: 10.1038/s44318-024-00103-7 (PMC11377571; doi:10.1038/s44318-024-00103-7)
Supplement: Supplementary file 14 — Expanded View Figures [file 44318_2024_103_MOESM14_ESM.pdf]

Expanded View Figures

Figure EV1. Associated with Fig. 1: Melanosomes are detected in non-cancerous cells in the tumor microenvironment.

(**A**) Images of compound nevi, in situ and vertical melanoma and lymph metastasis samples from two patients. Upper panels: Images of H&E-stained sections of compound nevi, in situ, and vertical melanoma and lymph metastasis samples from two patients. Black dashed lines demarcate the epidermal and dermal borders. Scale bars, 200 µm. Inset images show macrophages containing pigmented vesicles. Scale bars, 50 µm. Lower panels: Images of consecutive specimens stained for HMB45 (white), melanosomal marker GPNMB (green), and macrophage marker CD68 (red) and with DAPI for nuclei (blue). White dashed lines demarcate the epidermal and dermal borders. Scale bars, 200 µm. Inset images show the co-localization of macrophages with melanosomes. Scale bars, 50 µm. (**B**) Left: Images of H&E-stained sections of in situ melanoma specimens from two patients. Black dashed lines demarcate the epidermal and dermal borders. Scale bars, 200 µm. Right: Immunofluorescence images of consecutive samples stained for CD68 (red) and GPNMB (green) or FSP1 (red) and GPNMB (green). White dashed lines demarcate the epidermal and dermal borders. Scale bars, 200 µm. Inset images show keratinocytes within the epidermis containing melanosomes marked with white arrowheads. Scale bars, 50 µm.

© The Author(s)

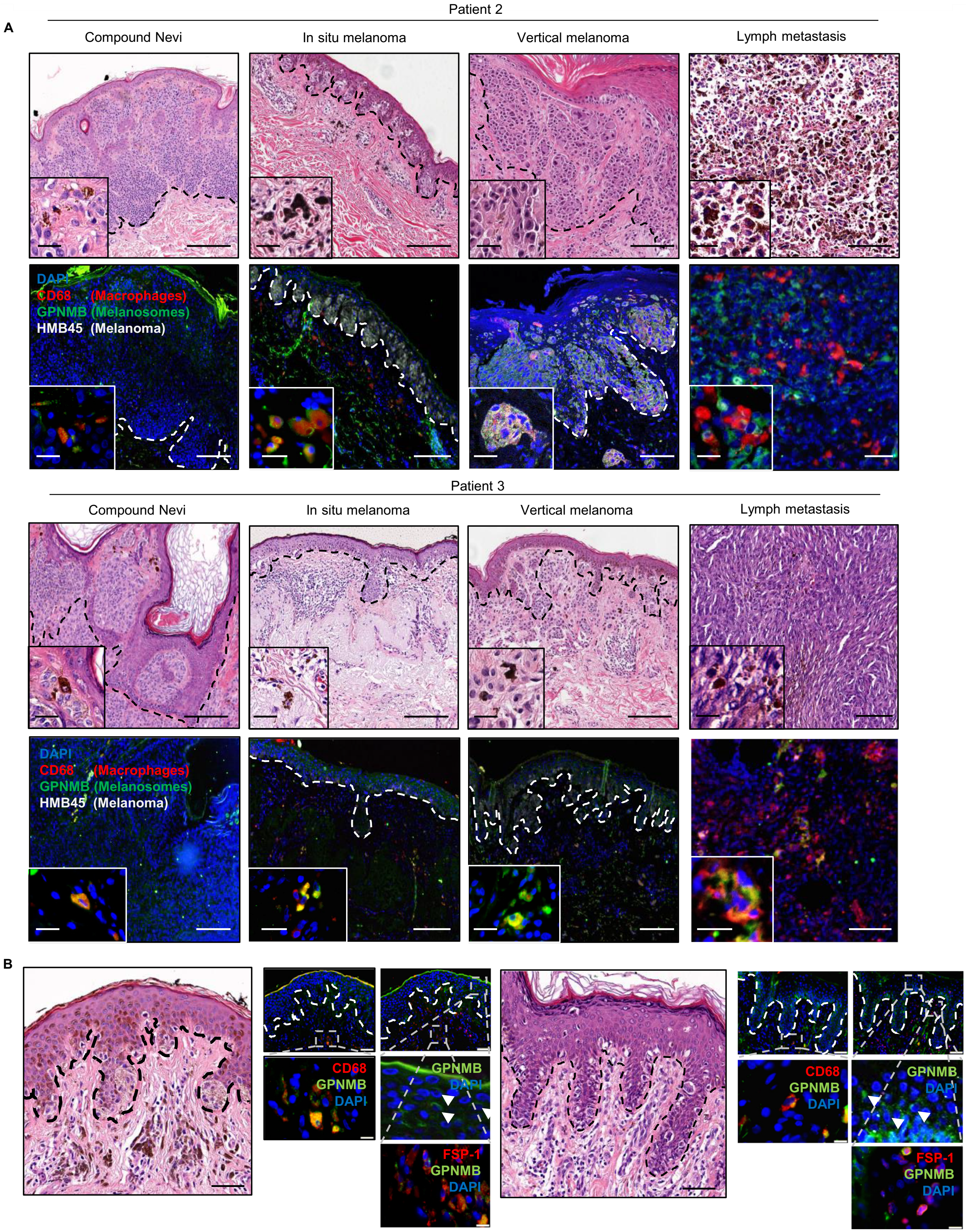
A
Patient 2
Compound Nevi
In situ melanoma
Vertical melanoma
Lymph metastasis
DAPI
CD68 (Macrophages)
GPNMB (Melanosomes)
HMB45 (Melanoma)
Patient 3
Compound Nevi
In situ melanoma
Vertical melanoma
Lymph metastasis
DAPI
CD68 (Macrophages)
GPNMB (Melanosomes)
HMB45 (Melanoma)
B
CD68
GPNMB
DAPI
GPNMB
DAPI
FSP-1
GPNMB
DAPI
CD68
GPNMB
DAPI
GPNMB
DAPI
FSP-1
GPNMB
DAPI

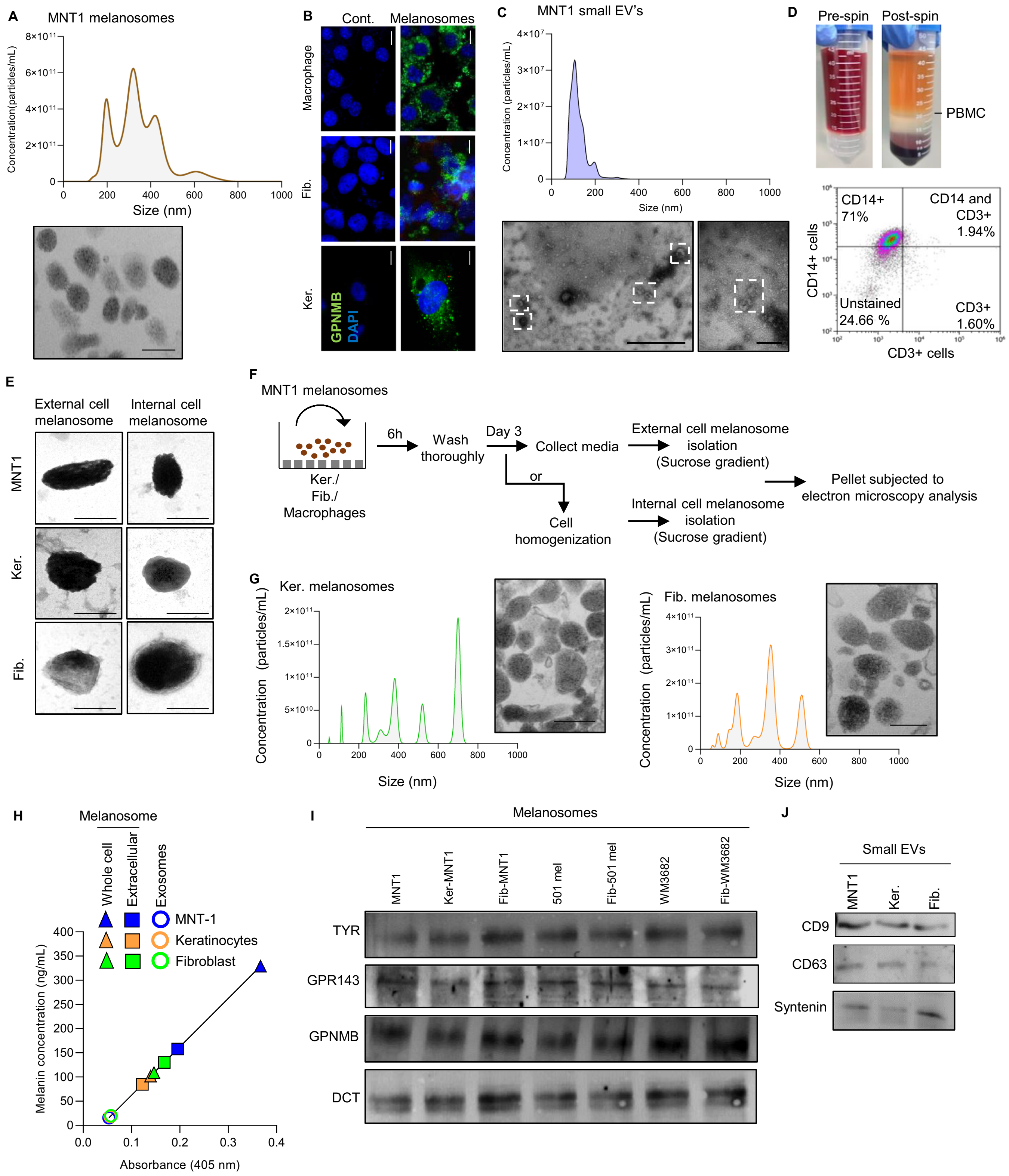
A
MNT1 melanosomes
Concentration(particles/mL)
Size (nm)
B
Cont.
Melanosomes
Macrophage
Fib.
Ker.
GPNMB
DAPI
C
MNT1 small EV's
Concentration (particles/mL)
Size (nm)
D
Pre-spin
Post-spin
PBMC
CD14+ 71%
CD14 and CD3+ 1.94%
Unstained 24.66 %
CD3+ 1.60%
CD14+ cells
CD3+ cells
E
External cell melanosome
Internal cell melanosome
MNT1
Ker.
Fib.
F
MNT1 melanosomes
Ker./ Fib./ Macrophages
6h
Wash thoroughly
Day 3
Collect media
or
Cell homogenization
External cell melanosome isolation (Sucrose gradient)
Internal cell melanosome isolation (Sucrose gradient)
Pellet subjected to electron microscopy analysis
G
Ker. melanosomes
Fib. melanosomes
Concentration (particles/mL)
Size (nm)
H
Melanosome
Whole cell
Extracellular
Exosomes
MNT-1
Keratinocytes
Fibroblast
Melanin concentration (ng/mL)
Absorbance (405 nm)
I
Melanosomes
MNT1
Ker-MNT1
Fib-MNT1
501 mel
Fib-501 mel
WM3682
Fib-WM3682
TYR
GPR143
GPNMB
DCT
J
Small EVs
MNT1
Ker.
Fib.
CD9
CD63
Syntenin

 © The Author(s)

Figure EV2. Associated with Fig. 2: Cell-to-cell transfer of melanoma cell-derived melanosomes occurs in the tumor microenvironment.

(**A**) NanoSight analysis (upper) and TEM analysis (lower) of melanosomes secreted from melanoma cells. Scale bars, 0.5 μm. (**B**) Immunofluorescence images of control keratinocytes, fibroblasts, and naïve macrophages and of cells cultured with melanosomes for 24 h. DAPI-stained nuclei appear blue. Scale bars, 10 μm. (**C**) NanoSight analysis (upper) and TEM analysis (lower) of small EVs secreted from melanoma cells. Scale bars, 0.5 μm left image and 0.2 μm for right image. Small EVs are highlighted with white dashed boxes. (**D**) Top: Photograph of peripheral blood mononuclear cell sample after Ficoll gradient centrifugation. Bottom: Flow cytometry analysis of monocytes isolated using a $CD14^+$ cell isolation kit; 71% of isolated cells were $CD14^+$. (**E**) TEM images of melanosomes from inside cells (right) and from conditioned media (left) of indicated samples. Scale bars, 0.2 μm. (**F**) Workflow of isolation of melanosomes from inside cells and from conditioned media after 3 days of culture. (**G**) NanoSight analysis (right) and TEM images (left) of melanosomes isolated from the keratinocyte and fibroblast cells cultured with MNT1 melanoma cell melanosomes. Scale bars, 0.5 μm. (**H**) Quantification of melanin concentration in samples isolated by differential centrifugation, gradient centrifugation, and ultra-centrifugation for secreted melanosomes, internalized melanosomes, and small EVs, respectively, from melanoma cells, keratinocytes, and fibroblasts; $n = 2$ independent experiments. (**I**) Western blot analysis for signature melanosomal proteins TYR, GPR143, GPNMB, and DCT in the melanosome fraction from indicated cells. (**J**) Western blot analysis for signature small EV proteins CD9, Syntenin, and CD63 in small EV fractions from indicated cells.

© The Author(s)

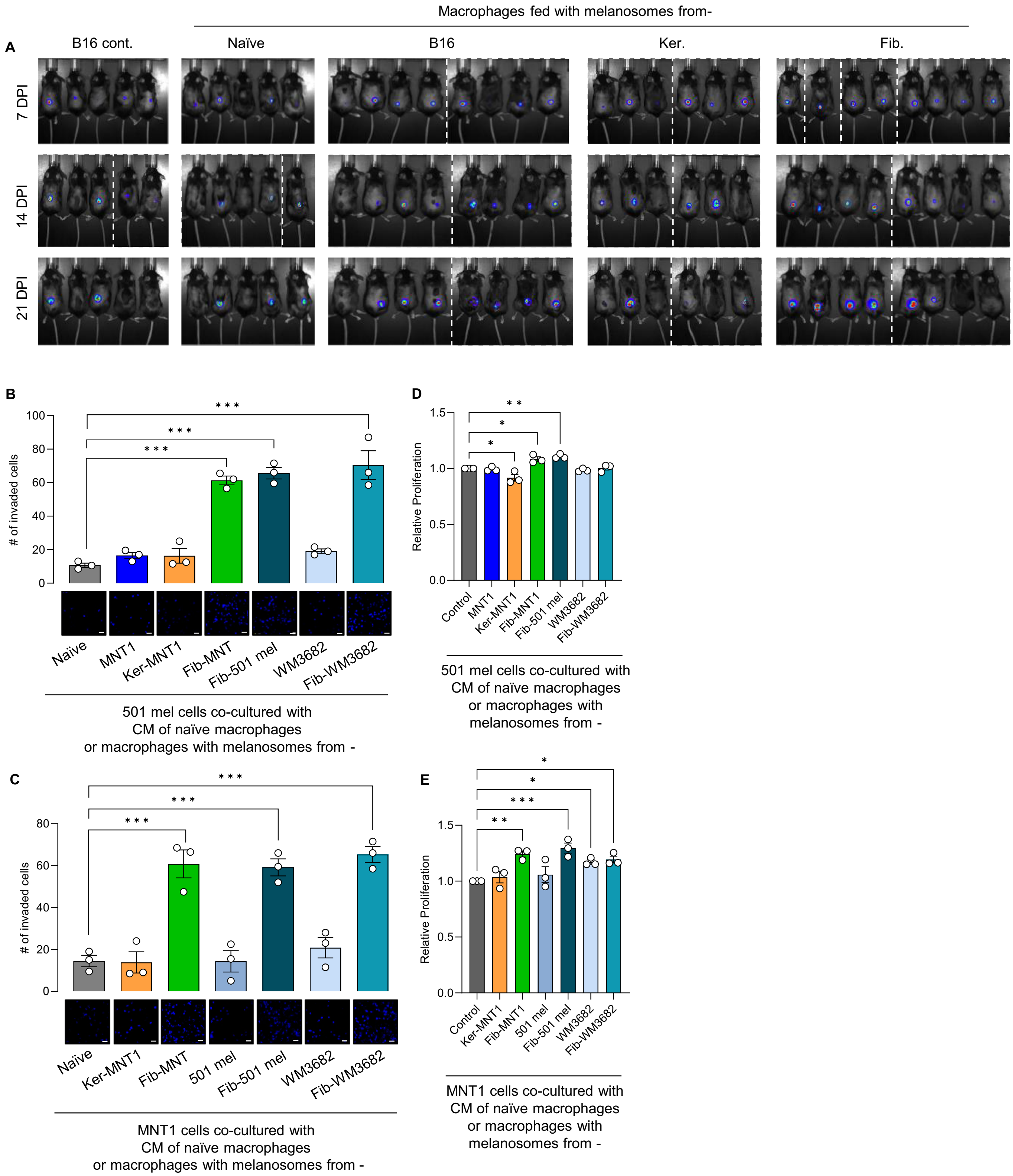
Macrophages fed with melanosomes from-
A
B16 cont.
Naïve
B16
Ker.
Fib.
7 DPI
14 DPI
21 DPI
B
# of invaded cells
Naïve
MNT1
Ker-MNT1
Fib-MNT
Fib-501 mel
WM3682
Fib-WM3682
501 mel cells co-cultured with
CM of naïve macrophages
or macrophages with melanosomes from -
C
# of invaded cells
Naïve
Ker-MNT1
Fib-MNT
501 mel
Fib-501 mel
WM3682
Fib-WM3682
MNT1 cells co-cultured with
CM of naïve macrophages
or macrophages with melanosomes from -
D
Relative Proliferation
Control
MNT1
Ker-MNT1
Fib-MNT1
Fib-501 mel
WM3682
Fib-WM3682
501 mel cells co-cultured with
CM of naïve macrophages
or macrophages with
melanosomes from -
E
Relative Proliferation
Control
Ker-MNT1
Fib-MNT1
501 mel
Fib-501 mel
WM3682
Fib-WM3682
MNT1 cells co-cultured with
CM of naïve macrophages
or macrophages with
melanosomes from -

 © The Author(s)

Figure EV3. Associated with Fig. 3: Macrophages cultured with melanosomes from different cell sources have heterogenous effects on cancer hallmarks.

(**A**) In vivo bioluminescent images of C57BL6 mice injected with B16-F10 mCherry cells expressing firefly luciferase reporter co-inoculated with BMDMs incubated with melanosomes derived from B16-F10 melanoma cells, fibroblasts, or primary keratinocytes. Images were taken on days 7, 14, and 21 post injection. Mice inoculated with B16-F10 cells and naïve macrophages were used as controls. Note: The representative images shown in Fig. 3B haven been taken as a representative of each group from the raw images shown here. (**B**) Upper: Numbers of invaded 501mel melanoma cells treated with conditioned media from macrophages cultured with melanosomes from indicated source cells for 24 h. $n = 3$ independent experiments. Lower: Representative images of invasion assay. Scale bar, 50 μm. (**C**) The proliferation of 501mel melanoma cells treated with the conditioned media of macrophages that had been cultured with melanosomes from indicated cells for 24 h relative to culture with conditioned media from naïve macrophages (control). $n = 3$ independent experiments. (**D**) Upper: Numbers of invaded MNT1 melanoma cells treated with conditioned media from macrophages cultured with melanosomes from indicated source cells for 24 h. $n = 3$ independent experiments. Lower: Representative images of invasion assay. Scale bar, 50 μm. (**E**) The proliferation of MNT1 melanoma cells treated with the conditioned media of macrophages that had been cultured with melanosomes from indicated cells for 24 h relative to culture with conditioned media from naïve macrophages (control). n = 3 independent experiments. Data information: In panel (**B–E**), error bars represent ±SEM and one-way ANOVA was performed for statistical analysis, $^*P \leq 0.05$, $^{**}P \leq 0.01$, $^{***}P \leq 0.001$ was considered significant.

© The Author(s)

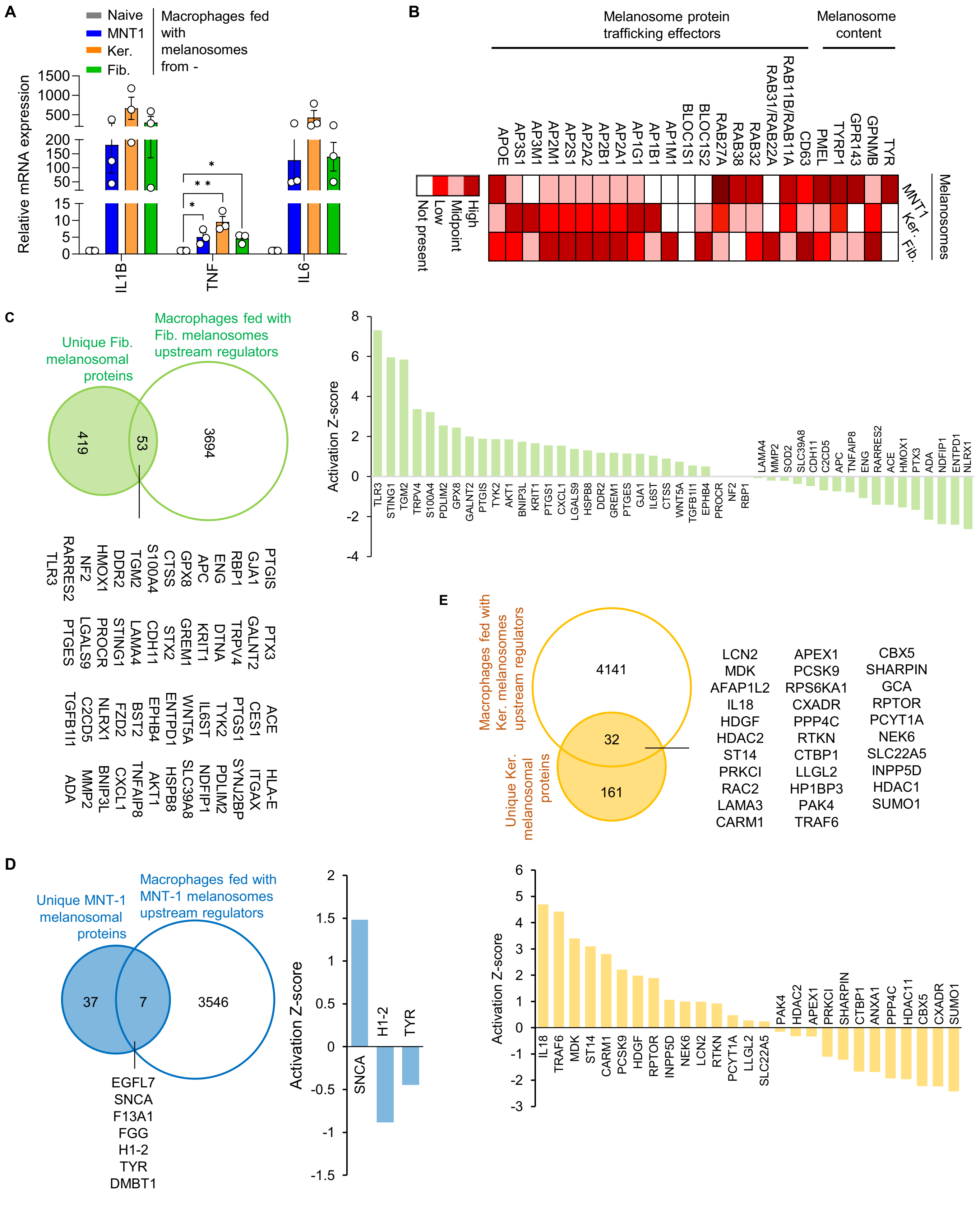
A
Naive
MNT1
Ker.
Fib.
Macrophages fed with melanosomes from -
Relative mRNA expression
IL1B
TNF
IL6
B
Melanosome protein trafficking effectors
Melanosome content
APOE AP3S1 AP3M1 AP2M1 AP2S1 AP2A2 AP2B1 AP2A1 AP1G1 AP1B1 AP1M1 BLOC1S1 BLOC1S2 RAB27A RAB38 RAB32 RAB31/RAB22A RAB11B/RAB11A CD63 PMEL TYRP1 GPR143 GPNMB TYR
Melanosomes
MNT1
Ker.
Fib.
High
Midpoint
Low
Not present
C
Unique Fib. melanosomal proteins
Macrophages fed with Fib. melanosomes upstream regulators
419
53
3694
Activation Z-score
D
Unique MNT-1 melanosomal proteins
Macrophages fed with MNT-1 melanosomes upstream regulators
37
7
3546
EGFL7
SNCA
F13A1
FGG
H1-2
TYR
DMBT1
Activation Z-score
E
Macrophages fed with Ker. melanosomes upstream regulators
Unique Ker. melanosomal proteins
4141
32
161
LCN2 MDK AFAP1L2 IL18 HDGF HDAC2 ST14 PRKCI RAC2 LAMA3 CARM1
APEX1 PCSK9 RPS6KA1 CXADR PPP4C RTKN CTBP1 LLGL2 HP1BP3 PAK4 TRAF6
CBX5 SHARPIN GCA RPTOR PCYT1A NEK6 SLC22A5 INPP5D HDAC1 SUMO1
Activation Z-score

© The Author(s)

Figure EV4. Associated with Fig. 4: Macrophages incubated with melanosomes from different cell types are phenotypically and functionally diverse.

(**A**) Relative mRNA expression of pro-inflammatory genes *IL1B*, *TNF*, and *IL6* in macrophages cultured with melanosomes from MNT1 melanoma cells, keratinocytes, and fibroblast cells normalized to expression in naïve macrophages; $n = 3$. (**B**) Heatmap of LFQ intensity of melanosomal proteins and trafficking effector proteins in melanosomes from indicated cell sources. $n = 3$ independent experiments. (**C**–**E**) Overlap between the upstream regulators predicted by IPA and unique melanosomal proteins and graphs depicting the activation Z-scores of the upstream regulators found in the overlap from (**C**) fibroblasts, (**D**) MNT1 melanoma cells, and (**E**) keratinocyte as identified in Fig. 4I,J. $n = 3$ individual donors for each melanophage and $n = 3$ independent experiments for melanosome isolation. Data information: In panel (**A**), error bars represent ±SEM. One-way ANOVA was performed for statistical analysis, $^*P \le 0.05$, $^{**}P \le 0.01$ was considered significant.

© The Author(s)

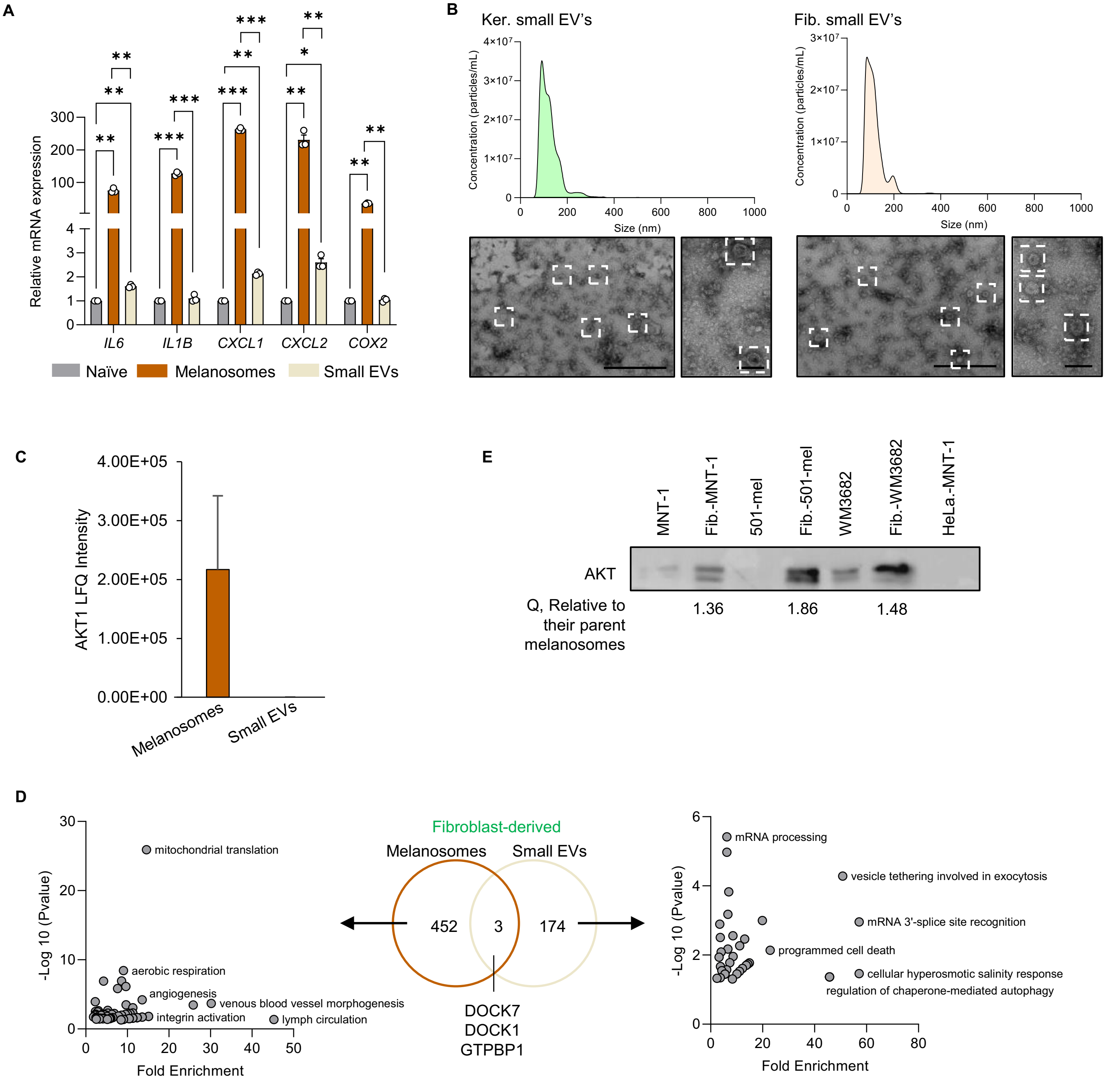

Figure EV5. Associated with Fig. 5: Fibroblasts melanosomes loaded with AKT1 induce angiogenesis in macrophages via the AKT1/mTOR pathway.

(**A**) mRNA levels of CAF-associated genes in dermal fibroblast cells upon culturing with small EVs or melanosomes. $n = 3$ independent experiments. (**B**) NanoSight analysis (upper) and TEM analysis (lower) of small EVs secreted from keratinocytes (Ker., left panel) and fibroblasts (Fib., right panel). Scale bars, 0.5 µm and 0.2 µm. Small EVs are highlighted with white dashed boxes. (**C**) LFQ intensity scores of the AKT1 protein levels in fibroblast-derived small EVs and melanosomes. $n = 2$ independent experiments of isolation of each EVs. (**D**) Center: Overlap between the uniquely expressed proteins in each fibroblast-derived small EVs or melanosomes compared to the MNT1 or keratinocyte-derived small EVs or melanosomes, by proteomics analysis. MNT1 and keratinocyte EVs were used as controls to identify specific proteins expressed by the EVs of the fibroblast cells. Left and right: Dot plots showing the significantly enriched biological processes of the genes further uniquely expressed by each EV. $n = 2$ independent experiments of isolation for each EVs. (**E**) Western blot analysis for AKT protein levels in melanosomal fractions isolated from the melanoma cells or fibroblast cells that were cultured with indicated melanoma melanosomes. Data information: In panel (**A**, **C**), error bars represent ± S.E.M. In panel (**A**), one-way ANOVA was performed for statistical analysis, $^*P \leq 0.05$, $^{**}P \leq 0.01$, $^{***}P \leq 0.001$ was considered significant.

 © The Author(s)
